# Selective Toluene Electrooxidation to Benzyl Alcohol

**DOI:** 10.1021/jacs.5c05986

**Published:** 2025-09-24

**Authors:** Madeleine K. Wilsey, Nathalia Cajiao, Aleksa Radovic, Michael L. Neidig, Yasemin Basdogan, Astrid M. Müller

**Affiliations:** † Materials Science Program, 6927 University of Rochester, Rochester, New York 14627, United States; ‡ Department of Chemistry, 6927 University of Rochester, Rochester, New York 14627, United States; § Department of Chemistry, University of Oxford, Inorganic Chemistry Laboratory, South Parks Road, Oxford OX1 3QR, United Kingdom; ∥ Department of Chemical Engineering, 6927 University of Rochester, Rochester, New York 14627, United States

## Abstract

We report a novel electrocatalytic approach that couples water oxidation with toluene oxidation in wet DMF or DMSO electrolyte. Electrocatalytic oxidation of water, a sustainable oxygen atom source, favored hydroxyl (^·̇^OH) radical formation over hydrogen peroxide and oxygen evolution because the organic solvent molecules suppressed O–O bond formation via hydrogen bonding. Water oxidation was catalyzed by laser-synthesized [NiFe]-(OH)_2_ nanosheets supported on hydrophilic carbon fiber paper anodes, enhancing water oxidation activity and toluene oxygenation efficiency. Under optimized conditions in wet DMF electrolyte, toluene oxidation achieved 100% selectivity for benzyl alcohol at an unprecedented conversion yield of 87%. In wet DMSO electrolyte, 100% selectivity for benzyl alcohol was obtained, albeit with significantly lower conversion yield. Combined experimental and computational results reveal a new mechanistic pathway, based on electrocatalytic water oxidation to ^·^OH radicals and protons. Protonation of DMF enabled the formation of H^+^-DMF–^·^OH radical complexes stabilized by hydrogen bonding among the radical, protonated and unprotonated DMF molecules, and water. This radical stabilization played a crucial role in promoting benzyl alcohol production. In contrast, DMSO consumed ^·^OH radicals to form methanesulfinic acid, limiting benzyl alcohol generation. The wet organic solvent environment additionally prevented overoxidation beyond the alcohol by stabilizing radical intermediates through hydrogen bonding networks, effectively arresting the reaction after the first oxygenation. Likewise, benzyl alcohol oxidation yielded benzaldehyde, with no overoxidation to benzoic acid. Our findings establish fundamental design principles for selective hydrocarbon oxidations by leveraging solvent-mediated interactions, with broad implications for sustainable chemical synthesis.

## Graphical abstract

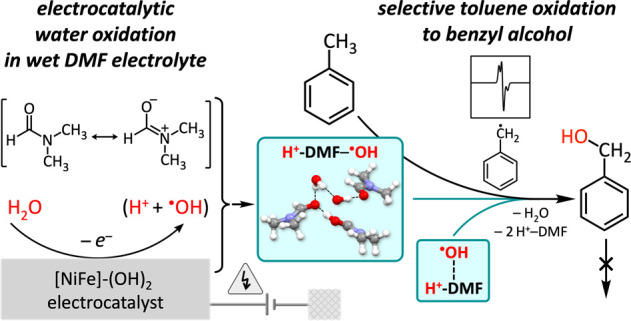

## Introduction

The selective oxidation of hydrocarbons to alcohols without overoxidation is a “holy grail” of chemistry with far-reaching applications in sustainable chemical manufacturing. The major challenges in hydrocarbon oxidations are overoxidation, selectivity issues, and the use of stoichiometric oxidants.^1–4^ Arresting the oxidation at the alcohol is particularly challenging because the first step possesses the highest kinetic barrier, while those of further oxidations are lower.^5,6^

Benzylic C–H activation functionalizes aromatic hydrocarbons to enable the production of value-added compounds, such as aryl alcohols.^7^ Previous studies on selective benzylic C–H oxidations to alcohols have been largely unsuccessful because of the high proclivity for further oxidation of alcohols to ketones.^8–13^ The resulting phenones, as well as benzylic monooxygenation products such as benzyloxy phthalimide^14^ or nitrooxylated^15^ derivatives, can be reduced to benzylic alcohols in a second step, albeit with efficiency issues.^16^ Previous methods for benzylic alcohol synthesis required expensive and/or toxic conditions, such as complex metal catalysts and stoichiometric or excess oxidants or mediators, which necessitate energy-intensive downstream separations,^17^ anhydrous solvents, and anaerobic conditions. Additionally, most of these methods led to complex product mixtures.^2,16,18–27^

Toluene is the simplest methylarene. Its monooxygenated product benzyl alcohol is an important commodity chemical with uses as a solvent in inks, paints, lacquers, resins, and paint strippers, in household cleaners, detergents, and personal care products, and as a food additive.^28^ Industrially, benzyl alcohol is produced by hydrolyzing benzyl chloride or hydrogenating benzaldehyde at high temperatures and pressures.^28^ Attempts have been made to lower the energy demand of benzyl alcohol production by alternative chemical oxidation routes, albeit with product selectivity issues.^5,19,20,22,24,27^

Unlike chemical oxidations, electrocatalysis eliminates the need for stoichiometric oxidants, enhancing atom economy.^29,30^ Furthermore, it operates under ambient to moderate pressures and temperatures and does not require the large-scale infrastructure of thermochemical processes, making it suitable for decentralized applications powered by renewable electricity.^29–31^ Electrochemical oxidation of toluene has been reported; however, achieving complete selectivity for a single product, particularly benzyl alcohol, remains challenging.^26,32–34^

Here, we report the selective electrooxidation of toluene to benzyl alcohol in wet LiClO_4_-supported *N*,*N*-dimethylformamide (DMF) or dimethyl sulfoxide (DMSO) electrolytes. Water serves as the oxygen atom source in these electrolytes. Traditional organic syntheses often use scarce, toxic oxidants, such as high-valent chromium, manganese, iron, cerium, or osmium oxo species.^35^ In contrast, water is an eco-friendly and scalable oxygen source. Water can be converted into oxidizing species by electrocatalytic water oxidation to facilitate advanced oxidation processes of organic molecules.^36^

We utilized laser-synthesized [NiFe]-(OH)_2_ nanosheets as water oxidation catalysts supported on hydrophilic carbon fiber paper.^37^ Laser-synthesized [NiFe]-(OH)_2_ nanoparticles are well understood and among the best nonprecious water oxidation electrocatalysts,^38–42^ producing oxygen (O_2_) at pH 14 and hydrogen peroxide (H_2_O_2_) at pH 7.^41^ Hydroxyl (^·^OH) radicals are also a possible product of water oxidation.^43^ However, in aqueous systems, ^·^OH radicals are rarely observed due to the high thermodynamic potential required for their formation. Instead, product speciation is directed toward the energetically favored generation of O_2_ and H_2_O_2_ in aqueous media.^43^ Hydrophilicity of the support enabled water wetting, which is essential for water oxidation at the [NiFe]-(OH)_2_ nanocatalyst. Another reason for rendering carbon fiber paper hydrophilic is the deposition of laser-synthesized [NiFe]-(OH)_2_ nanosheets from aqueous suspension, as to not introduce organic contaminants.

We show how electrolyte engineering and concomitant access to new mechanistic pathways leads to 100% selectivity for benzyl alcohol with an unprecedentedly high conversion yield of 87%. We performed density functional theory (DFT) calculations^44–50^ to elucidate the molecular electrooxidation mechanism of toluene and oxygenates and to reveal the reasons for arresting the transformation after the first oxygenation.

## Results and Discussion

### Anode

We employed [NiFe]-(OH)_2_ nanosheets as water oxidation electrocatalysts, synthesized via pulsed laser in liquid synthesis. This method eliminates the need for surfactants, allowing precise control over catalyst surface chemistry and microenvironments—an advantage not achievable with conventional chemical syntheses, which rely on surfactants for size control.^51^ The removal or exchange of surfactants is often incomplete and poorly reproducible due to entropic penalties, leading to organic contamination in complex electrocatalytic reaction networks. In contrast, laser-synthesized nanoparticles are inherently surfactant-free, making them more suitable for understanding electrocatalytic systems. Additionally, laser synthesis can be scaled up to industrially relevant outputs.^51^

We laser-synthesized [NiFe]-(OH)_2_ nanocatalysts analogous to a published protocol.^38^ The resulting material exhibited layered double hydroxide structure and nanosheet morphology, evident from X-ray diffraction (XRD) and scanning electron microscopy (SEM) data (Figure 1a,b). The XRD peaks showed significant broadening (Figure 1a), attributed to small crystallite size and stacking faults, such as turbostratic disorder within the hydrotalcite-like structure, consistent with prior reports on laser-synthesized [NiFe]-(OH)_2_ nanosheets.^40^ SEM and energy-dispersive X-ray spectroscopy (EDX) data revealed that laser synthesis resulted in [Ni_0.76_Fe_0.24_]-(OH)_2_ nanosheets (Supporting Information, Figure S1a).

**Figure 1:**
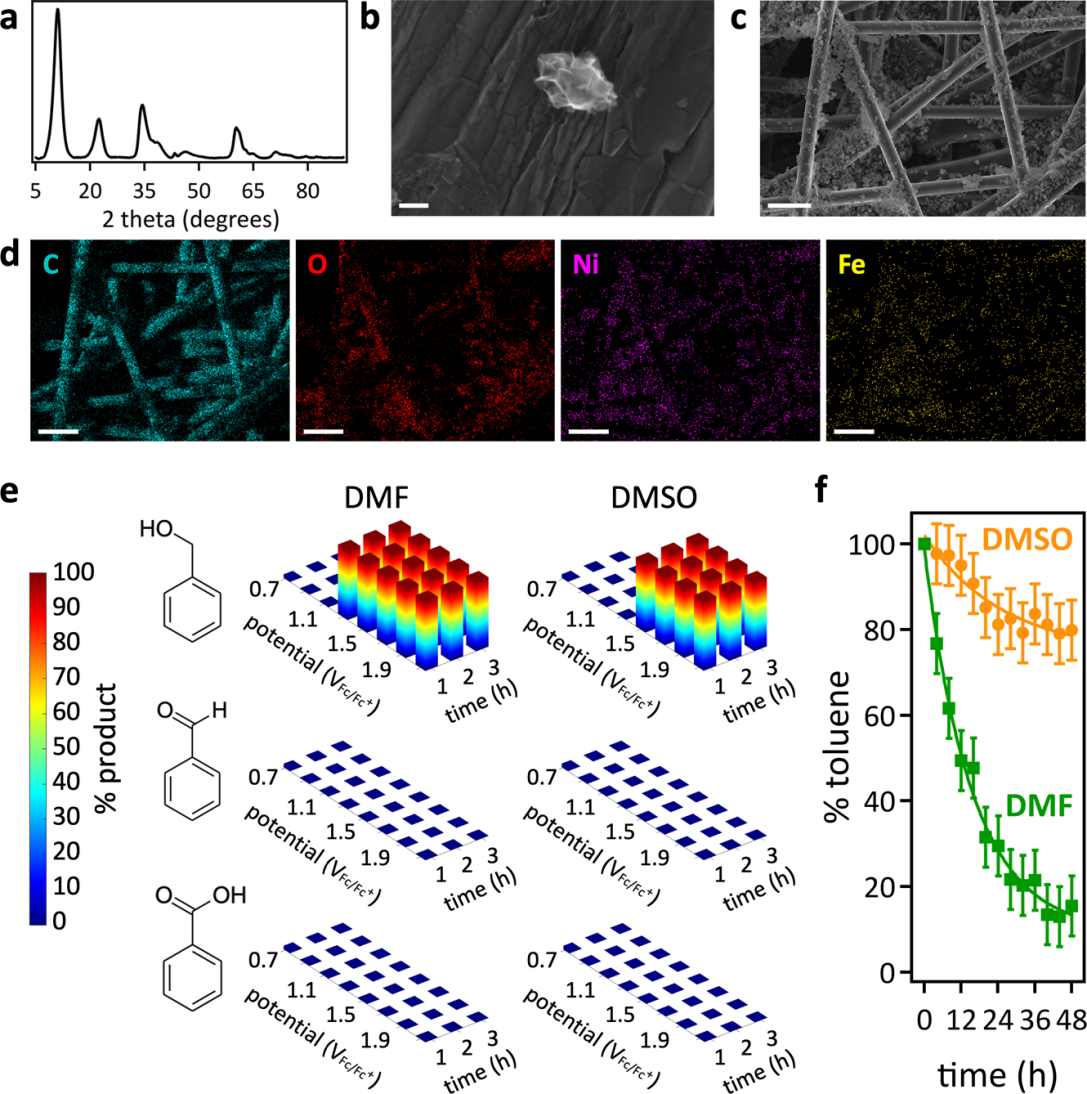
(a) XRD data of laser-synthesized [Ni_0.76_Fe_0.24_]-(OH)_2_ nanosheets. SEM images with a scalebar of (b) 100 nm and (c) 20 μm of laser-synthesized [Ni_0.76_Fe_0.24_]-(OH)_2_ nanosheets (light) on hydrophilic carbon fiber paper (dark). (d) Corresponding SEM-EDX maps with scalebars of 20 μm. (e) Product selectivity as a function of applied potential and reaction time, derived from NMR data shown in Figures S8–S13. Panel 1e presents a subset of a larger data set; complementary data collected in electrolytes with lower water content are shown in Figure S7. (f) Toluene conversion yields obtained from NMR data shown in Figure S17 of electrolyte aliquots collected at the specified reaction times during toluene electrooxidation at 2.1 V. Toluene electrooxidation was conducted in LiClO_4_-supported DMF or DMSO electrolytes with 7.0 vol % water, electrocatalyzed by laser-synthesized [Ni_0.76_Fe_0.24_]-(OH)_2_ nanosheets on hydrophilic carbon fiber paper.

The laser-synthesized [NiFe]-(OH)_2_ nanosheets were supported on hydrophilic carbon fiber paper,^37^ which provides a high surface area of 468 cm^2^ per geometric cm^2^, as previously reported.^52^ This high surface area increased the alkaline water oxidation mass activity of the nanocatalyst by 60-fold compared to flat electrodes at 1 V overpotential.^37^ The enhanced anodic oxidation improves the identification and quantification of reaction intermediates and products. The integrated anodes on hydrophilic carbon fiber paper demonstrated stability (Figure S1b) and incorporated [Ni_0.76_Fe_0.24_]-(OH)_2_ nanosheets within the three-dimensional carbon fiber network, fully utilizing the high internal surface area of the carbon architecture (Figure 1c,d).

### Electrooxidation of Toluene

Electrooxidation of toluene, catalyzed by laser-synthesized [Ni_0.76_Fe_0.24_]-(OH)_2_ nanocatalysts on hydrophilic carbon fiber paper, was performed in LiClO_4_-supported DMF, DMSO or acetonitrile electrolytes containing 2.0, 4.5, or 7.0 vol % water. Water and liquid substrate concentrations are given in vol % instead of molarity due to inherent volume changes upon mixing. These changes arise from disruptions in the liquid structure, caused by alterations in intermolecular forces and the resulting increase in entropy.^53,54^ Since molarity depends on the final solution volume, vol % more accurately represents concentration by reflecting initial liquid volumes, avoiding complications from mixing-induced volume changes. Maintaining a constant water-to-organic solvent ratio across different substrates and concentrations is essential for distinguishing the mechanistic contributions of water oxidation from those of subsequent radical reactions.

Lithium perchlorate is commonly used in nonaqueous and mixed solvent systems due to its high electrochemical stability^55–59^ and good solubility in both DMF^60,61^ and DMSO.^62^ Perchlorate is widely regarded as a redox-inactive and stable supporting electrolyte anion.^63^ NMR data show that addition of 0.1 M LiClO_4_ to wet DMF or DMSO (with 7.0 vol % water) did not change the spectra, confirming that LiClO_4_ is compatible with these solvents (Figure S2a). Alternative lithium salts present challenges in our system. Lithium fluoride, while containing redox-stable fluoride, is impractical due to its low solubility in water (∼1 g L^–1^) and DMF (∼0.1 mM).^64,65^ Lithium chloride is highly soluble in both solvents, but chloride is not redox stable under our conditions and undergoes anodic oxidation to produce toxic chlorine,^66–68^ consuming current in this undesired side reaction. LiPF_6_, another common salt in battery applications, hydrolyzes in the presence of water to form PF_5_^–^ and HF,^55,69,70^ making it incompatible with wet organic electrolytes.

We also evaluated lithium bis­(trifluoromethanesulfonyl)­imide (LiTFSI), a redox-inactive salt widely used in the lithium-ion battery field.^71^ However, battery electrolytes are typically dry and do not contain DMF or DMSO.^72,73^ In our system, LiTFSI exhibited incomplete solubility in wet DMF. In wet DMSO, LiTFSI reacted upon mixing, as evidenced by NMR spectra showing a decrease in the water signal relative to wet DMSO without LiTFSI (Figure S2b), suggesting undesired side reactions. These results confirm that lithium perchlorate is the most appropriate supporting electrolyte for the present system.

We conducted all electrocatalysis experiments in an undivided cell (photograph of setup shown in Figure S3a) because the wet DMF and DMSO electrolytes are incompatible with ion exchange membranes that would separate the anodic and cathodic compartments.^74–77^ Nevertheless, we attempted to use a Nafion membrane in wet DMF electrolyte and observed that the Nafion immediately swelled upon contact with the electrolyte, as expected (photographs shown in Figure S3b). While a divided cell would offer the advantage of distinguishing oxidative from reductive processes, including possible two-electron oxygen reduction to H_2_O_2_,^78^ we were unable to employ a membrane-divided configuration. Although porous frits, often made of glass, are used in electrochemical systems such as reference electrodes, they function by restricting mass transport based on size.^79^ If organic solvent molecules are able to pass through, the frits cannot effectively separate species involved in the water/oxygen system. Therefore, we conducted all experiments in an undivided cell. To assess whether oxygen reduction could contribute to H_2_O_2_-mediated pathways, we conducted control experiments with the electrolyte continuously purged with argon to eliminate O_2_. No significant differences in product formation were observed between Ar-purged and ambient conditions (Figure S4). Moreover, the addition of H_2_O_2_ to the reaction mixture resulted in diminished benzyl alcohol formation relative to that observed in the wet DMF and DMSO electrolytes (Figure S5). The use of undivided cells is common in oxidative electrosynthesis.^32,80–84^

Products were identified and quantified using nuclear magnetic resonance (NMR) spectroscopy with an internal standard and headspace gas chromatography (GC). Radical intermediates were detected by electron paramagnetic resonance (EPR) spectroscopy, with EPR simulation parameters provided in Tables S1–S3.

We used applied potentials of 0.7–2.1 V because we observed the onset of product generation within this potential range. All potentials are reported relative to the ferrocene/ferrocenium (Fc/Fc^+^) redox couple, a standard reference for calibrating electrodes in organic electrolytes.^85^ Cyclic voltammograms of toluene in wet DMF or DMSO with 7.0 vol % water are shown in Figure S6. We do not report overpotentials because they are not defined in wet organic electrolytes; the standard potential for water oxidation is unknown in these media and is only established in aqueous systems.^39,86^ The overpotential represents the potential difference between a half-reaction’s standard potential and the experimentally observed potential at which a specific current density is achieved, determined by the redox reaction rate.^63^ A quantity related to overpotential is the organic-solvent-associated redox potential of H_2_O/O_2_ in organic media, which strongly depends on solvent identity and water content.^87^ However, this has not been established for water oxidation in the wet DMF and DMSO electrolytes used in this study. The thermodynamic potentials could, in principle, be approximated by estimating the free energies of reaction from the standard free energies of formation and the free energies of solvation of each reactant and product in neat solvents. This would provide a useful approximation, although it would not yield the true free energy of reaction because it does not account for the water content and its effect on reaction thermodynamics. While the standard free energies of formation are solvent-independent and known,^88^ we were only able to find solvation free energy values for solvents other than DMF and DMSO.^53,89^ Therefore, the thermodynamic potential cannot be estimated this way for our system.

Three types of overpotential—activation, concentration, and ohmic—impact electrochemical energy efficiency.^86^ Catalysts are designed to lower the activation overpotential, which is related to the kinetic energy barrier that must be overcome for a reaction to proceed.^39^ The concentration overpotential arises from reactant depletion at the electrode surface due to mass transport limitations, which can be mitigated by convection;^63^ thus, we stirred the electrolyte in this study. Ohmic overpotential, or *iR* drop, results from internal resistance within the electrochemical cell, including ionic resistance of the electrolyte and electronic resistance of cell components. It is influenced by cell geometry and degree of *iR* compensation,^90^ which can be performed by two methods: (1) real-time correction using the potentiostat’s built-in positive feedback or current interruption function, or (2) postacquisition correction using an uncompensated resistance value obtained either from single-frequency impedance measurements (ZIR),^90^ or by fitting the high-frequency region of electrochemical impedance spectroscopy (EIS) data with an appropriate equivalent circuit.^91^ The assumption behind using the uncompensated resistance value derived from impedance spectroscopy is that the potential drop between the working and reference electrodes arises solely from the electrochemical double layer and the solution resistance. This may be valid for well-defined, structurally uniform electrodes such as platinum plates, where the electrode surface itself is catalytic.^91^ However, for electrodes that are structurally and chemically more complex, particularly layered structures such as the nickel–iron layered double hydroxide nanocatalysts on hydrophilic carbon fiber paper used in this work, the meaning of the uncompensated resistance can differ, and applying the full high-frequency resistance is likely to result in overcompensation.^91^ This may explain why some studies apply only partial compensation.^91^

The choice of *iR* compensation percentage is highly empirical and without theoretical support.^91^ In general, the potentiostat uses the energy needed for *iR* compensation, so that *iR* compensation primarily lowers reported potential values rather than actual energy consumption. However, *iR* compensation significantly affects benchmarking metrics and complicates comparisons across the literature.^91^ Consequently, all potential values in this study are reported without *iR* compensation. For reference, a 100% *iR* compensation has been reported for electrochemical propylene epoxidation via water activation,^92^ without a rationale for choice of this percentage. If 100% *iR* compensation were applied on-the-fly during electrocatalysis in our system, the maximum applied potential of 2.1 V in wet (7.0 vol % water) DMF and DMSO electrolytes would decrease to 1.3 V.

At the applied potentials where product formation occurred, we observed only benzylic C–H activation products—benzyl alcohol, benzaldehyde, and benzoic acid—without any aromatic ring oxygenates, evident from NMR data (Figures 1e and S7–S17). Control experiments at open circuit potential did not generate any products (Figure S18). Headspace GC analysis showed no carbon dioxide formation (Figure S19), ruling out benzoic acid oxidation via the Kolbe reaction.^93^ Additionally, no Kolbe reaction products were detected (Figures S8–S17). Unlike the toluene electrooxidation in wet DMF and DMSO electrolytes of this work, previously reported chemical oxidations of toluene also occurred at the aromatic ring, alongside the benzylic carbon, leading to undesirable product mixtures.^24^ Acetonitrile, a commonly used organic electrolyte,^63^ has been utilized in previous studies of toluene electrooxidation.^33,94^ Electrooxidation in wet acetonitrile electrolyte failed to produce a single, selective product (Figures S7 and S14–S16), suggesting that wet DMF and wet DMSO enable new mechanistic pathways for selective oxygenation.

Toluene electrooxidation in DMF electrolyte with 7.0 vol % water selectively produced benzyl alcohol, with an onset potential of 1.3 V (Figure 1e). At an applied potential of 2.1 V, this system achieved 100% selectivity for benzyl alcohol with an exceptionally high conversion yield of 87% (Figures 1e,f and S17), with a faradaic efficiency of 31%. The absence of detectable side products (Figures S8–S17) indicates that the remaining current was consumed by hydroxyl (^·^OH) radical recombination to regenerate water. Conversion yields reported here correspond to values obtained after 48 h of reaction time, unless otherwise noted. Starving this system of toluene produced a mixture of benzyl alcohol and a DMF–OH species (Figure S20). In DMSO electrolyte with 7.0 vol % water, toluene electrooxidation selectively yielded benzyl alcohol, albeit with a higher onset potential of 1.5 V and a lower toluene conversion yield of only 21% (Figures 1e,f and S17), with a faradaic efficiency of 19%. Remarkably, selectivity for benzyl alcohol as the first oxygenation product was maintained at all reaction times in both wet (7.0 vol % water) DMF and DMSO electrolytes.

Water was necessary for toluene electrooxidation in DMSO and DMF electrolytes, despite the high polarity of these organic solvents.^95,96^ In the absence of added water in the organic electrolyte, toluene conversion was negligible at 2.1 V, and lithium dendrites formed at the counter electrode (Figure S3c–f), indicating that Li^+^ from LiClO_4_ was the most easily reducible species in solution. This suggests that in electrolytes where water was added, cathodic proton reduction balanced the overall redox reaction. Higher water concentrations in DMSO and DMF electrolytes increased both product selectivity for benzyl alcohol and toluene conversion yields (Figures S7 and S21).

Water activation has previously been utilized in electrochemical propylene epoxidation at an oxidized palladium–platinum alloy catalyst, where epoxidation proceeds via electrophilic attack by metal-bound peroxo intermediates.^92^ However, our data reveal a novel mechanistic pathway for toluene oxidation via ^·^OH radicals, *in situ* generated by water oxidation at the nonprecious [NiFe]-(OH)_2_ electrocatalyst in wet DMF and DMSO electrolytes, leveraging solvent-mediated interactions. A fundamental distinction of our approach is that water serves as the source of ^·^OH radicals while simultaneously generating a nearby proton for each ^·^OH radical. Our work builds on extensive literature exploring the role of ^·^OH radicals in organic oxidations but differs in its mechanism and electrolyte environment.

In aqueous media, ^·^OH radical formation is thermodynamically disfavored.^43^ However, in wet DMF and DMSO electrolytes, organic solvent molecules surround water molecules and form extensive hydrogen bonding networks.^39,41,43^ Here, we exploit these water–organic solvent interactions that prevent O–O bond formation during water oxidation to suppress H_2_O_2_ and O_2_ evolution, thereby enabling ^·^OH radical generation via water oxidation in wet DMF and DMSO electrolytes.

In wet DMF electrolytes with ample water (≥4.5 vol %), we observed complete selectivity for benzyl alcohol. DMF can act as both an electrophile and a nucleophile.^97^ This neutral ability results from the amide resonance,^98,99^ which can induce a captodative effect that stabilizes carbon-based radical intermediates.^100^ Based on data shown below, we propose a novel mechanistic pathway here, where DMF, along with electrocatalytic water oxidation, *in situ* forms a mediator for ^·^OH radicals. This proposed mechanism explains the exceptionally high toluene conversion yield of 87% while maintaining 100% selectivity for benzyl alcohol.

### EPR Evidence of Radicals

Theoretical predictions suggest that toluene electrooxidation proceeds via a benzyl radical intermediate,^101^ in contrast to the established mechanism of *p*-xylene oxidation, which begins with electron transfer from the aromatic ring to form π-radical cations.^102^ We detected organic radicals in *ex situ* EPR spectra of electrolytes post electrocatalysis in wet (7.0 vol % water) LiClO_4_-supported DMF or DMSO. To disentangle the complex reaction network, we used electrolytes with toluene, with benzene (instead of toluene) and without substrate. The short lifetimes of oxygen- and carbon-based radicals that were generated in this work dictate that these radicals reside within the anode microenvironment and are unable to diffuse into the bulk electrolyte. For instance, benzyl radicals have a lifetime *t* of 1.4 μs in organic solvents.^103,104^ To estimate the diffusion distance *d* away from the electrode, we used the equation $d=\sqrt{2Dt}$ ,^63^ with *D* the diffusion coefficient of toluene. We were unable to identify literature values of *D* for toluene in the specific electrolytes used in this study. A reported value for toluene self-diffusion (*D* = 2.9 × 10^–3^ cm^2^ s^–1^) was found,^105^ but it is not applicable to the solvent systems employed here. Therefore, we experimentally determined the relevant diffusion coefficients using ^1^H-DOSY (diffusion ordered spectroscopy) NMR measurements (Table S4). In wet DMF and wet DMSO electrolytes, we obtained *D* values of (1.11 ± 0.02) × 10^–5^ and (6.00 ± 0.04) × 10^–6^ cm^2^ s^–1^, respectively. Based on these values, we estimate the benzyl radical diffusion distance to be 0.6 μm in wet DMF and 0.05 μm in wet DMSO electrolyte. Consequently, ^·^OH and organic radical reactions and spin trapping of radicals must occur within the anode microenvironment, leading to low concentrations of EPR-active species in the bulk solution. Therefore, the detection of these radicals is not trivial.

The room temperature EPR spectrum of benzyl radicals spin trapped with 2-methyl-2-nitrosoporpane (MNP) is known from radiolysis of neat benzyl alcohol with 3 MeV electrons.^106^ Here, we collected room-temperature EPR data of wet (7.0 vol % water) DMF and DMSO electrolytes post-toluene electrooxidation at 2.1 V, using MNP or N-*tert*-butyl α-phenyl nitrone (PBN) as spin traps (Figure S22). Associated simulation values are listed in Tables S1 and S2. EPR data of MNP spin trapped DMSO electrolyte did not show a discernible EPR signal. Therefore, we used PBN, a widely used spin trap,^106^ which revealed the presence of peroxo species, consistent with reported PBN EPR spectra.^107,108^ However, this signal obscured any potential benzyl radical detection. Analogous experiments with DMF electrolyte revealed no discernible EPR signal for PBN spin trapping. Conversely, MNP spin trapping of DMF electrolyte post toluene electrooxidation resulted in the formation of the MNP–MP adduct, which can form at room temperature,^109,110^ masking any potential benzyl radical signal.

Low-temperature EPR data of wet DMF and DMSO electrolytes post-toluene electrooxidation at 2.1 V, spin-trapped with MNP, collected at 10 K, revealed a signal at 3330 G, indicative of organic radicals. Associated simulation values are listed in Table S3.^111^ This supports our hypothesis that radical-mediated reactions played a role in toluene electrooxidation to benzyl alcohol. In all cases, irrespective of the presence and chemical identity of the substrate, the signal shape remained virtually identical, while the signal intensity varied (Figure 2a). Postelectrocatalysis EPR spectra of wet DMF electrolyte containing toluene, benzene, or no substrate showed intensities of the signal in the order of toluene > benzene > no substrate, indicating decreasing radical concentrations and the presence of DMF-based radicals (Figure 2a). The EPR signal of wet DMF electrolyte without substrate confirmed the formation of DMF-based radicals (Figure 2a).

**Figure 2:**
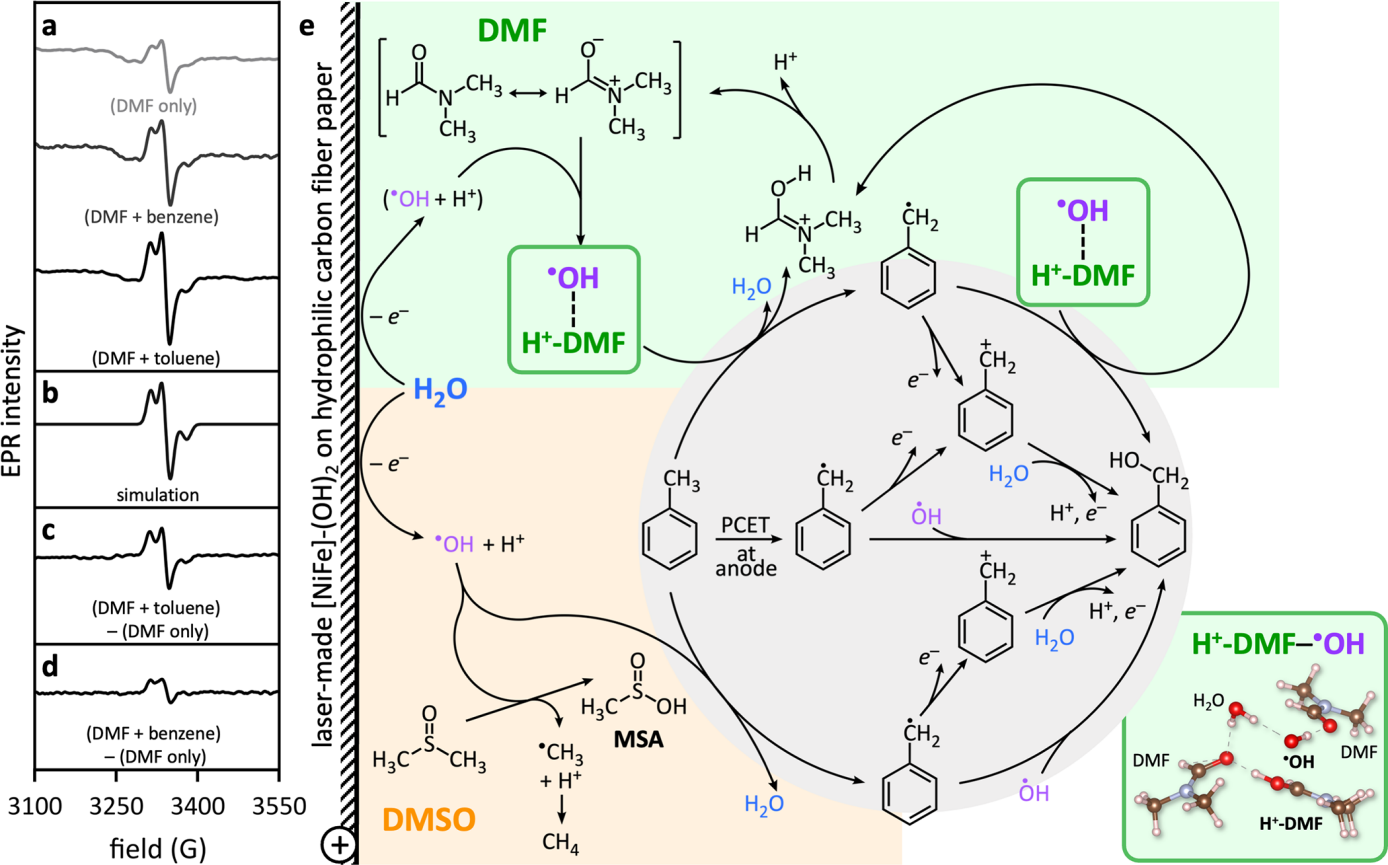
(a) Post electrooxidation EPR data of wet DMF electrolyte without substrate (top), with benzene (middle), and with toluene (bottom). (b) Simulation of the bottom EPR spectrum of panel (a) with *g* values *g*_1_ = 2.008, *g*_2_ = 2.006, and *g*_3_ = 2.0014, and hyperfine constants of *A* = [0 0 91.4] MHz. (c) Difference EPR signal after subtracting data of the DMF electrolyte without substrate from those with toluene. (d) Difference EPR signal after subtracting data of the DMF electrolyte without substrate from those with benzene. All EPR spectra were spin trapped by MNP, collected at 10 K, and are plotted with identical *y*-axis scaling. EPR spectra were collected after 24 h of electrocatalysis at 2.1 V with 7.0 vol % water in LiClO_4_-supported DMF electrolyte, using the same EPR tube. (e) Proposed toluene electrooxidation mechanism in wet DMF (highlighted in green) or DMSO electrolyte (highlighted in orange), showing anodic water oxidation and reactions of ^·^OH radicals. MSA, methanesulfinic acid. The DFT-calculated structure of the protonated DMF–^·^OH radical complex (framed green) is shown on the bottom right. Element colors: H, white; C, gray; N, blue; O, red. Dashed lines indicate hydrogen bonding interactions. Highlighted in gray are possible toluene to benzyl alcohol pathways via a benzyl radical intermediate.

In contrast, the EPR data for the wet DMSO electrolyte after electrooxidation showed a clear signal only in the reaction mixture containing toluene, while systems without a substrate or with benzene exhibited no discernible signals (Figure S23a), consistent with the absence of a DMSO-based radical. The observed EPR signals overall indicate that the toluene oxidation pathway is radical-mediated. Furthermore, the differences in signal strength suggest that wet DMF plays a supportive role in radical formation and stabilization, which is absent in the wet DMSO electrolyte. In order to understand the origin of these radicals, EPR spectra were collected in the same EPR tube, ensuring that signal strength variations were not influenced by path length differences.

Subtraction of EPR data of wet DMF electrolyte post electrocatalysis at 2.1 V without substrate from those with toluene resulted in the difference spectrum shown in Figure 2c. An analogous difference spectrum obtained from subtracting EPR data of wet DMF electrolyte without substrate from those with benzene shows the presence of a weaker signal (Figure 2d), attributable to organic radicals.

In wet DMSO electrolyte after electrocatalysis at 2.1 V, the difference spectrum obtained from subtracting data collected without substrate from those with toluene demonstrated a clear signal (Figure S23b), indicative of an organic radical, likely the benzyl radical, as computationally predicted.^101^ The difference spectrum resulting from subtracting data collected without substrate from those with benzene in DMSO did not show an EPR signal (Figure S23c), ruling out the formation of an aromatic ring-based radical, corroborated by the absence of aromatic ring oxygenate products in toluene electrooxidation (Figures S11–S13).

### Toluene Oxidation Mechanism

The formation of a benzyl radical from toluene can occur through three possible mechanistic pathways: (i) H atom abstraction by an ^·^OH radical, (ii) direct proton-coupled electron transfer (PCET) at the anode, or (iii) H atom abstraction by an organic solvent based radical (Figure 2e, highlighted in gray). Postelectrocatalysis EPR data of wet DMSO electrolyte without substrate showed no discernible signal (Figure S23a, light gray spectrum), ruling out a mechanistic pathway involving a DMSO-based radical in the C–H activation of toluene. Our observation that higher water concentrations in DMSO electrolytes increased benzyl alcohol selectivity suggests that benzylic H atom abstraction by an ^·^OH radical is the most likely pathway for benzyl radical generation in wet DMSO electrolyte with 7.0 vol % water.

In DMSO electrolyte with 2.0 or 4.5 vol % water, all three benzylic toluene oxidation products were observed at all applied potentials (Figure S7). DMSO is a known nucleophilic oxidant under anhydrous conditions,^112^ consistent with the limited selectivity for benzyl alcohol in DMSO electrolyte with ≤4.5 vol % water. In the absence of sufficient water, nucleophilic attack of DMSO at the toluene methyl group can directly oxidize toluene to benzaldehyde via a single oxygen transfer step.^112^ The ^·^OH radicals generated from water oxidation can react with DMSO molecules,^113,114^ creating methyl radicals,^115^ which are highly unstable.

These methyl radicals can either abstract a hydrogen atom from toluene, generating benzyl radicals and ultimately producing methane,^116^ or participate in an addition reaction with toluene to form xylenes.^117,118^ However, NMR data showed no evidence of xylene formation (Figures S11–S13), ruling out this widely accepted pathway. While headspace GC analysis did not detect methane (Figure S19), this does not definitively exclude the methane-generating pathway due to the high solubility of methane in DMSO,^119^ which can hinder gas-phase detection. Although a characteristic NMR signal at 0.23 ppm has been reported for 1 atm of methane dissolved in deuterated DMSO,^120^ we did not detect a corresponding signal in our NMR data.

Instead, our findings suggest an alternative mechanistic route for toluene electrooxidation in wet DMSO electrolyte, involving the formation of methanesulfinic acid (MSA) through the reaction of DMSO with ^·^OH radicals (Figure 2e, highlighted in orange). MSA is the principal product formed when DMSO reacts with ^·^OH radicals.^121^ MSA was detected in our NMR data (Figures S11–S13, S24a, S5c, S25b, and S26b). MSA formation consumes ^·^OH radicals, explaining the lower toluene conversion observed in DMSO compared to DMF, despite both electrolytes achieving complete selectivity for benzyl alcohol.

In wet DMF electrolyte with 7.0 vol % water, our data support a novel mechanistic pathway for electrochemical oxygenation of toluene, in which DMF, along with electrocatalytic water oxidation, *in situ* forms a hydrogen bonding stabilized mediator for ^·^OH radicals (Figure 2e, highlighted in green). Our EPR data of wet DMF electrolyte after electrooxidation revealed the presence of DMF-based radicals (see above), which we attribute to protonated DMF–hydroxyl (H^+^-DMF–^·^OH) radical complexes. Water oxidation forms *in situ* a nearby proton for every generated ^·^OH radical. These protons can react with DMF molecules to produce O-protonated DMF (Figure 2e, highlighted in green),^122^ evident from NMR data (Figure S20) and further corroborated by DMF acidification experiments (Figure S27). O-Protonation of amides has been reported in H_2_SO_4_.^122^ While N-protonation of amides is also possible, it is less likely in unstrained amides.^123^ With a proton affinity of 887.5 kJ mol^–1^,^124^ DMF can undergo O-protonation by the protons released by water oxidation.

Our DFT calculations indicate that wet protonated DMF stabilizes ^·^OH radicals through hydrogen bonding by 22.4 kcal mol^–1^, compared to 32.8 kcal mol^–1^ for nonprotonated DMF (Figure S28). In the wet protonated DMF cluster, a coordinated H_2_O molecule is also present and plays a key role in stabilizing the ^·^OH radical in conjunction with H^+^-DMF (Figure S28). In the absence of H_2_O, the ^·^OH radical was not stable and spontaneously formed H_2_O, indicating the importance of explicit water in preserving the ^·^OH radical. To confirm that the formation of a water-oxidation-derived ^·^OH radical is possible in wet DMF, we computed a standard potential of 3.65 V for the oxidation of water to hydroxyl radical in the DMF cluster, which is consistent with the well-established fact that generating ^·^OH radicals in nonaqueous media requires significantly more energy than in water (2.73 V).^125,126^

To evaluate whether these protonated solvation structures are thermodynamically favorable compared to unprotonated clusters, we computed the free energy change for the protonated cluster formation. The resulting Δ*G* = −11.4 kcal mol^–1^ confirms that protonated clusters are energetically favored, validating their relevance under localized acidic conditions. The resulting H^+^-DMF–^·^OH radical complexes (calculated structure shown on the bottom right of Figure 2e) significantly enhance benzyl alcohol production, with toluene conversion rates in DMF more than four times higher than in DMSO (Figure 1f). DMSO cannot be protonated under the electrocatalysis conditions used here (Figure 2e, highlighted in orange), as corroborated by NMR data (Figures S11–S13 and S27). Only superacidic media can protonate DMSO at the S=O moiety.^127^ Because of this lack of protonation, MSA does not stabilize ^·^OH radicals like the H^+^-DMF–^·^OH radical complex.

The concentration of the generated H^+^-DMF–^·^OH radical complexes depended on the presence of aromatic substrate molecules. In the absence of a substrate, the EPR signal was the weakest (light gray spectrum in Figure 2a), yet still detectable. Notably, radical formation from DMF in the presence of ^·^OH has only been observed under highly energetic conditions, such as pulsed laser photolysis or MeV radiolysis. For example, DMF radicals were generated upon intense 248 nm KrF laser irradiation in smog-chamber photo-oxidation experiments.^128^ Similarly, 266 nm laser flash photolysis yielded DMF radicals, attributed to hydrogen abstraction from DMF by cumyloxyl and benzyloxyl radicals.^129^ Given that DMF absorbs in the ultraviolet spectral region, including at 248 and 266 nm,^130^ DMF radical formation via direct DMF photolysis, which has been reported,^131^ cannot be ruled out. Additionally, transient absorption spectra have indicated carbon-centered radicals following 2.3 MeV radiolysis of aqueous DMF solutions.^132^ However, radical formation has also been observed in neat DMF under MeV electron beam radiolysis.^131^ Together, these findings suggest that reactions between ^·^OH and DMF as well as direct DMF radical formation are unlikely under the mild conditions employed in our study, supporting our assignment of the EPR signal to H^+^-DMF–^·^OH radical complexes.

Adding benzene as a substrate increased the EPR signal, likely due to the stabilization of radicals by the π-bond system. Benzene can stabilize radicals through the π-bond network of its aromatic ring.^133^ We note that we did not detect products from aromatic ring C–H activation (Figures S8–S13), ruling out the involvement of a ring radical reactive intermediate. This, along with the observed EPR signal of wet DMF electrolyte without substrate postelectrocatalysis, suggests that the detected radicals correspond to H^+^-DMF–^·^OH radical complexes. Finally, EPR data of wet DMF electrolyte with toluene showed the strongest signal, indicating the formation of organic—possibly benzyl—radicals in addition to the presence of H^+^-DMF–^·^OH radical complexes (Figure 2a).

The yield of benzyl alcohol formation correlated with the amount of detected radicals in wet DMF electrolyte (Figure S29), suggesting a contribution of benzyl radicals. Taken together, our EPR data and the exclusive formation of benzylic electrooxidation products support the hypothesis that electrocatalytic oxidation of toluene to benzyl alcohol in wet DMF or DMSO electrolytes likely proceeds via a benzyl radical intermediate.

### Anode Microenvironment Processes

Benzyl alcohol can form from a benzyl radical intermediate through two possible mechanistic pathways: (i) by an addition reaction of a ^·^OH radical, created by water oxidation at the anode, or (ii) via nucleophilic trapping of the benzyl radical by a water molecule through PCET (Figure 2e, highlighted in gray). Rebound of the benzyl radical onto the catalyst surface, as observed for metal oxides,^134–136^ does not appear to be a viable pathway for C–O bond formation in our system, because it would not account for the observed 100% selectivity for benzyl alcohol in wet DMF and DMSO, but lack of selectivity in wet acetonitrile (Figures 1, S7, and S14–S16).

Simple carbon radicals are inert to water,^137^ requiring oxidation of the benzyl radicals to carbocations for rapid reaction with water.^138^ In bulk solution, the probability of two low-concentration radicals reacting with each other is low, making water the more likely reactant for the benzyl radical to benzyl alcohol reaction (via the carbocation) in solution. This mechanism via the carbocation would require benzyl radicals to be oxidized at the anode. However, since ^·^OH radicals are also generated at the anode, the probability of benzyl radicals reacting with ^·^OH radicals within the anode microenvironment is significantly higher than in solution. As a result, toluene electrooxidation in this solid electrocatalyst system fundamentally differs from radical reactions in solution.

To confirm that toluene electrooxidation to benzyl alcohol occurs within the anode microenvironment, we conducted quenching experiments targeting surface adsorbed ^·^OH radicals. For this purpose, we added allyl alcohol to the toluene-containing wet organic electrolytes, as it selectively quenches surface adsorbed ^·^OH radicals.^139–141^ In DMSO, the presence of allyl alcohol completely suppressed benzyl alcohol production (Figure S24a), confirming that the ^·^OH radical pathway was active (Figure 2e, highlighted in orange), and that the reaction occurred within the anode microenvironment, where ^·^OH radicals were generated and readily reacted with allyl alcohol. Since electrocatalytic water oxidation inherently produces ^·^OH radicals and protons at the anode surface—and given the short lifetimes of ^·^OH radicals,^142^ which restrict their diffusion to only a few nanometers—the toluene electrooxidation process remains confined to the anode microenvironment. The reaction via addition of an ^·^OH radical to toluene, followed by addition of an ^·^OH radical to the generated benzyl radical intermediate, is a 2-electron–2-proton process, which is kinetically more favorable than the 3-electron–3-proton carbocation pathway (Figure 2e). Therefore, and because of and the spatial confinement of radical reactions to the anode microenvironment, the direct reaction of benzyl radicals with ^·^OH radicals near the anode surface appears plausible.

Unlike toluene electrooxidation in wet DMSO electrolyte, where allyl alcohol completely suppressed the reaction, its addition to wet DMF electrolyte reduced the toluene-to-benzyl alcohol conversion rate by a factor of 2 but did not fully inhibit the reaction (Figure S24b). This suggests that both surface and solution-phase reactions occurred. In wet DMF electrolyte, where *in situ* generated H^+^-DMF–^·^OH radical complexes facilitate toluene oxygenation, a direct reaction with benzyl radicals is also possible due to the higher concentration of H^+^-DMF–^·^OH radical complexes compared to the shorter-lived free ^·^OH radicals. Additionally, the kinetic advantage of the 2-electron–2-proton pathway over the 3-electron–3-proton carbocation pathway further supports the feasibility of benzyl radicals reacting directly with H^+^-DMF–^·^OH radical complexes.

### Why Is the Electrooxidation Arrested at Benzyl Alcohol?

Toluene electrooxidation in wet (7.0 vol % water) DMSO or DMF electrolyte achieved 100% selectivity for benzyl alcohol, even at an exceptionally high conversion yield of 87% (Figure 1e,f). This performance exceeds previously reported studies on toluene electrooxidation, where selectivity for benzyl alcohol or benzaldehyde declined as toluene conversion increased.^19,143–145^ Computations rationalize why our electrooxidation of toluene in wet DMSO or DMF electrolyte was arrested after the first oxygenation (Figure 3). All calculated energies are in Table S5. We note that the columns in Figure 3 correspond to three distinct reactions. The first, second, and third columns represent reactions starting from toluene (Figure 3a,d), benzyl alcohol (Figure 3b,e), and benzaldehyde (Figure 3c,f), respectively, in pristine wet DMF (green) or wet DMSO (orange). While the reaction products are benzyl alcohol in panels a and d, benzaldehyde in panels b and e, and benzoic acid in panels c and f, the organic solvents also undergo chemical changes during each reaction: DMF becomes protonated, and DMSO converts into MSA. Therefore, in the DFT calculations, the reactants were placed in pristine wet solvents, while species en route to the products were modeled in wet DMF or wet DMSO containing one H^+^-DMF or one MSA molecule, respectively.

**Figure 3:**
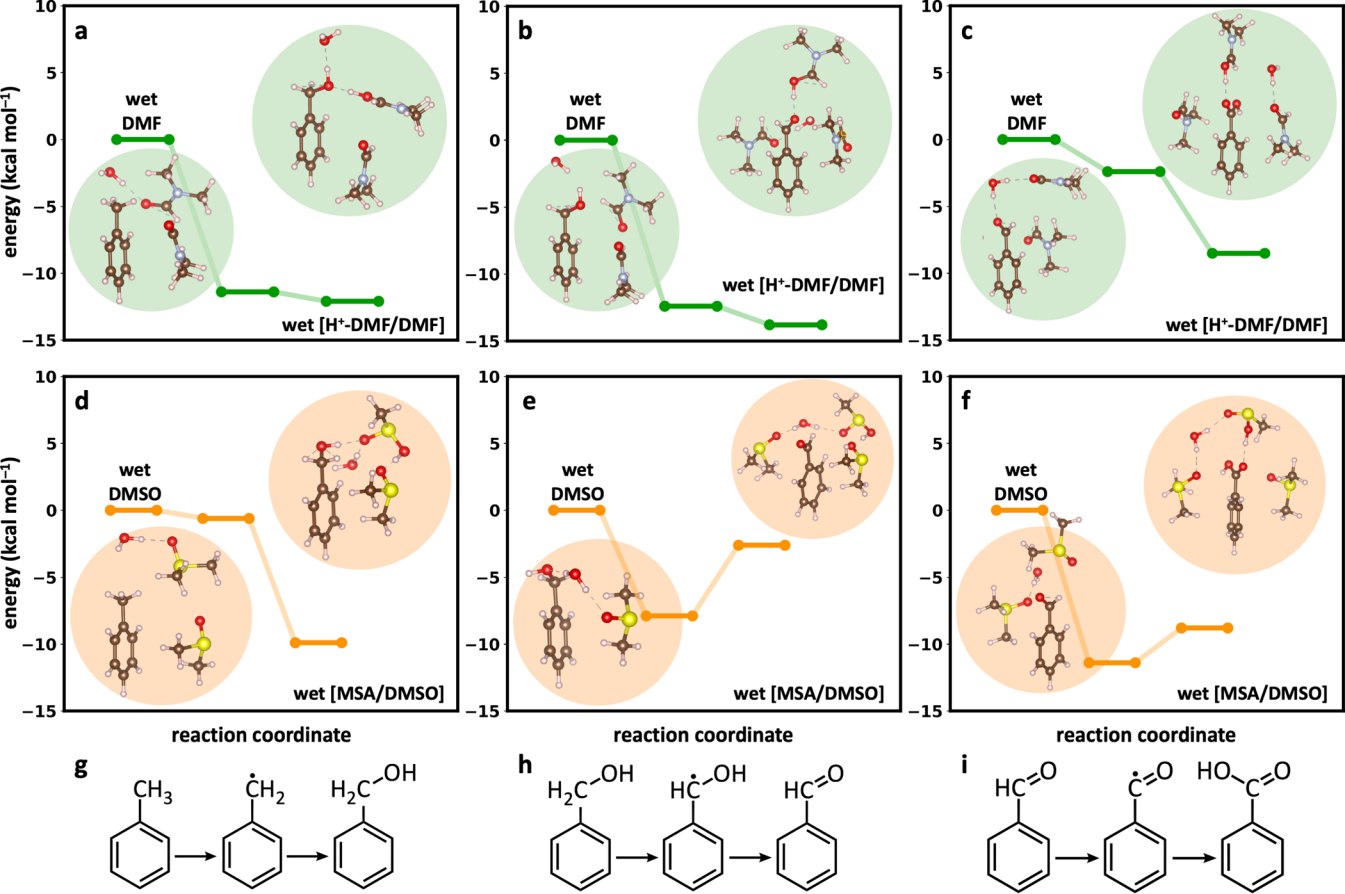
Calculated energies that have been normalized with respect to the reactant step in each solvent, (a–c) wet DMF, (d–f) wet DMSO. Each reaction intermediate is modeled with three explicit solvent molecules and one explicit water molecule together with the conductor-like polarizable continuum model (CPCM). In some clusters, we only show two explicit solvents molecules for visualization purposes. Full geometries are presented in Figures S31–S33. (g–i) Molecular representation of the respective one-step oxygenations. Element colors: H, white; C, gray; N, blue; O, red; S, yellow. Dashed lines indicate hydrogen bonding interactions.

We conducted DFT calculations to evaluate the stability of each reaction intermediate in DMF and DMSO solvents, using a mixed implicit/explicit solvation model to investigate solvent cluster formation.^44–50^ To realistically capture localized protonation effects in the solvation environment, our cluster models include one H^+^-DMF molecule per solvation shell, with all remaining DMF molecules treated as neutral. This configuration ensures a net cluster charge of +1, which accurately reflects the localized acidic conditions expected near the electrode interface under electrochemical oxidation. Similarly, the cluster model for the reaction in DMSO includes one methanesulfonic acid (MSA) molecule in the solvation shell, while the remaining solvent molecules consist of DMSO. The Cartesian coordinates of all optimized clusters are provided in the SI.

With 7.0 vol % water in DMF or DMSO, our system experimentally consists of one water molecule per three organic solvent molecules. Since we found that the presence of a coordinated H_2_O molecule is essential for stabilizing the ^·^OH radical in DMF (see above and Figure S28), we also included an explicit water molecule in the modeling of toluene oxidation. The DFT calculations show that inclusion of this explicit water molecule is crucial to achieve convergence of all structures and to accurately reflect how water stabilizes radical intermediates in wet organic solvents (Figure S30), a conclusion supported by our experimental data. Explicit solvent environments consisting of one H^+^-DMF molecule, two neutral DMF molecules, and one water molecule are denoted wet [H^+^-DMF/DMF] solvent, and solvent clusters composed of one MSA molecule, two DMSO molecules, and one water molecule are denoted wet [MSA/DMSO] solvent in the context of DFT calculations of methyl aryl oxidations. Strikingly, the explicit water molecule particularly stabilizes the benzyl radical intermediate en route from toluene to benzyl alcohol in wet [H^+^-DMF/DMF] solvent (Figure S30), where we experimentally observed 100% benzyl alcohol selectivity and 87% conversion yield (Figure 1). In addition, all calculated reactant energies in DMF or DMSO are higher with explicit water than without, presumably because the added water molecule disrupts the arrangement of aprotic solvent molecules around the methyl aryl species.

Initially, we modeled toluene in DMF, which resulted in the formation of a stabilized benzyl radical intermediate in wet [H^+^-DMF/DMF] solvent, followed by the formation of benzyl alcohol (Figure 3a). Benzyl alcohol in wet [H^+^-DMF/DMF] solvent establishes a stable hydrogen-bonding network with an overall clustering energy of 18.6 kcal mol^–1^. Electrooxidation halts at the benzyl alcohol stage because further oxidation in wet [H^+^-DMF/DMF] solvent, which would involve the formation of a benzaldehyde radical, requires 3.9 kcal mol^–1^ of energy to disrupt the hydrogen-bonding network of benzyl alcohol in the wet [H^+^-DMF/DMF] solvent. This demonstrates selective oxygenation of toluene in DMF to form benzyl alcohol.

To directly assess whether protonated solvent clusters preferentially stabilize benzylic alcohol relative to other species along the oxidation pathway, we computed the free energy change associated with adding a proton to solute–solvent clusters of key intermediates (Table S6). Our results show that protonation is energetically favorable for toluene (Δ*G* = −13.8 kcal mol^–1^), benzyl alcohol (Δ*G* = −17.3 kcal mol^–1^) and benzaldehyde (−8.7 kcal mol^–1^), indicating that protonated environments can stabilize oxidized intermediates. This stabilization may contribute to product accumulation (e.g., benzyl alcohol) under electrochemical conditions.

In wet [MSA/DMSO] solvent, where our experiments demonstrate the formation of MSA from the reaction of DMSO with ^·^OH radicals (Figure 2e, highlighted in orange), toluene generates a mildly stabilized benzyl alcohol radical that subsequently converts to benzyl alcohol. Benzyl alcohol in wet [MSA/DMSO] solvent forms a stable solvent cluster, supported by a hydrogen-bonding network with a clustering energy of 21.7 kcal mol^–1^, as illustrated in Figure 3d. Electrooxidation selectively terminates at the benzyl alcohol stage because the oxidation of benzyl alcohol to the benzyl alcohol radical is 6.0 kcal mol^–1^ uphill in wet [MSA/DMSO] solvent. The molecular representation of the single oxygenation of toluene to benzyl alcohol is shown in Figure 3g. Full geometries are presented in Figures S31–S33.

While solvent cluster formation energies provide valuable insights into solute–solvent interactions, they do not account for the intrinsic thermodynamics of bond cleavage. To address this, we also computed the Gibbs free energies of homolytic C–H bond dissociation for toluene, benzyl alcohol, and benzaldehyde. The computed bond dissociation free energies are 81.7 kcal mol^–1^ (toluene), 67.4 kcal mol^–1^ (benzyl alcohol), and 89.3 kcal mol^–1^ (benzaldehyde), in good agreement with literature values for benzylic C–H bonds.^146^ The computed values are included in Table S7. The relatively high C–H bond strength of benzaldehyde further supports the resistance of benzyl alcohol to overoxidation.

### Increased Selectivity at Higher Potential

Our results demonstrate that achieving complete selectivity for benzyl alcohol in wet DMF or DMSO electrolyte requires both a high water concentration and a relatively high applied potential, with the added benefit of high toluene conversion. This contrasts with many electrochemical reactions where increasing the applied potential does not lead to the desired upgraded chemical.^147–151^ Our data, proposed mechanism and computations rationalize this unexpected finding of higher selectivity for a single nonoveroxidized product at higher applied potential. In wet DMF electrolyte, toluene oxidation critically depends on the availability of *in situ* produced protons and ^·^OH radicals to form the H^+^-DMF–^·^OH radical complexes that oxidize toluene (Figure 2e, highlighted in green). Additionally, wet [H^+^-DMF/DMF] solvent helps arrest the oxygenation after the first step (Figure 3a). Both protons and ^·^OH radicals originate from electrocatalytic water oxidation, which becomes more efficient as the water concentration in the electrolyte increases. Typically, in water oxidation electrocatalysis, increasing the applied potential shifts selectivity toward H_2_O_2_ and O_2_ evolution, as the kinetic barriers of multielectron processes are more easily overcome.^41,43^ However, in our system, increasing the applied potential from 1.3 to 2.1 V in DMF electrolyte with 7.0 vol % water resulted in a more than 23-fold increase in organic radical yield (Figure S29), while maintaining 100% selectivity for benzyl alcohol, which exhibited an onset potential of 1.3 V (Figure 1e).

### Water Oxidation to ^·^OH Radicals in Wet DMF or DMSO Electrolytes

With 7.0 vol % water in the organic electrolytes, the molar ratio of DMF or DMSO to water is 3:1, at which DMF and DMSO form extensive hydrogen bonding networks between water molecules and the organic oxygen and hydrogen atoms.^152,153^ These strong water–solvent interactions preclude the typical O–O bond formation during water oxidation at the [Ni_0.76_Fe_0.24_]-(OH)_2_ nanocatalyst, even at high applied potential, thereby uniquely favoring ^·^OH radical over O_2_ and H_2_O_2_ production. Headspace GC data confirm the absence of O_2_ generation (Figure S19).

The ^·^OH radicals could also originate from advanced oxidation processes via electro-Fenton^36,154^ or Haber-Weiss mechanisms. In general, Fenton or Haber–Weiss reactions generate ^·^OH radicals *in situ* from H_2_O_2_ in the presence of a transition metal such as iron.^36,154–156^ Consequently, the iron-containing [Ni_0.76_Fe_0.24_]-(OH)_2_ nanocatalyst could, in principle, act as an initiator for these propagation reactions, leading to oxygen radical species, if H_2_O_2_ was present.

To rule out the involvement of these pathways, we conducted experiments in which 7.0 vol % of 30% aqueous H_2_O_2_ was used instead of water in DMF or DMSO electrolyte for toluene electrooxidation at 2.1 V. When 30% aqueous H_2_O_2_ was used instead of water in the DMF electrolyte, benzyl alcohol formation exhibited a time lag of 1 h (Figure S5a), and the water peak in NMR data grew from 0.5 to 4 h reaction time (Figure S5b). Similarly, replacing water with 30% aqueous H_2_O_2_ in DMSO electrolyte completely inhibited benzyl alcohol formation over a reaction period of 4 h (Figure S5a, c).

Our finding that H_2_O_2_ is detrimental to toluene oxidation, combined with the high toluene conversion yield and exclusive selectivity for benzyl alcohol, suggests that H_2_O_2_ was not generated at the anode during electrocatalytic water oxidation in the wet organic electrolytes. Furthermore, unlike water oxidation, Fenton and Haber–Weiss reactions do not generate a nearby proton for each ^·^OH radical. The local acidification resulting from water oxidation enhances the thermodynamic driving force of ^·^OH radicals, effectively accelerating their reactions with other molecules. For example, the thermodynamic potential for the reaction ^·^OH → H_2_O is 2.72 V at pH 0 and 2.31 V at pH 7.^36,157^ Additionally, *in situ* protonation of DMF is essential for stabilizing complexes between DMF molecules and ^·^OH radicals. However, a time lag was also observed in wet DMSO electrolyte, which cannot form complexes with ^·^OH radicals. This suggests that, at initially high H_2_O_2_ concentrations, competing reactions occurred between ^·^OH radicals and H_2_O_2_ as well as between ^·^OH radicals and toluene, leading to the observed time lag in both DMF and DMSO electrolytes. Although H_2_O_2_ can scavenge ^·^OH radicals, its reaction rate is 2 orders of magnitude slower than that of ^·^OH radicals with organic molecules.^158–160^

Overall, our results suggest that the suppression of O_2_ and H_2_O_2_ evolution—driven by the strong intermolecular interactions of DMF or DMSO with water—dictates the water oxidation selectivity, which in turn governs the selectivity of toluene electrooxidation, with ^·^OH radicals enabling selective toluene oxygenation to benzyl alcohol. This finding also explains the lack of benzylic product selectivity in wet acetonitrile electrolyte, as acetonitrile lacks oxygen atoms necessary to establish strong hydrogen bonding networks with water. Consistent with this, water oxidation by laser-synthesized [NiFe]-(OH)_2_ nanocatalysts in wet acetonitrile electrolyte has been shown to produce both H_2_O_2_ and O_2_.^41^

Our findings reveal that the availability of ^·^OH radicals governs the product selectivity of toluene oxidation. Complete selectivity for benzyl alcohol in wet DMF or DMSO electrolytes was only achieved when sufficient ^·^OH radicals were generated at the anode via water oxidation. This required either a high water content in the electrolyte (≥4.5 vol % in DMF or 7.0 vol % in DMSO) or higher applied potentials under low-water conditions. For example, in DMF electrolyte with only 2.0 vol % water, benzoic acid was detected alongside benzyl alcohol at applied potentials ≤1.1 V. However, at ≥1.3 V and ≥3 h reaction time, benzyl alcohol became the sole product (Figures S7 and S8). Starving the reaction network in DMF electrolyte of water likely promoted the direct oxidation of benzyl radicals with molecular O_2_ from ambient air as a competing pathway, leading to benzoic acid formation and explaining the absence of benzaldehyde. This aligns with previous reports of thermochemical C–H activation of toluene with molecular O_2_ yielding benzoic acid.^161^ The formation of benzaldehyde, alongside benzyl alcohol, requires H_2_O_2_,^162^ which was absent under our conditions.

In wet DMSO electrolyte, the presence of MSA, originating from the reaction of DMSO with ^·^OH radicals, is the basis for 100% selectivity for benzyl alcohol (Figure 2e, highlighted in orange, and Figure 3d). The ^·^OH radicals consumed in MSA formation are unavailable for toluene oxidation, necessitating a water concentration of 7.0 vol % in DMSO electrolyte to achieve complete benzyl alcohol selectivity. In contrast, in DMF electrolyte, a lower water concentration (≥4.5 vol %) was sufficient for 100% selective benzyl alcohol production (Figures S7–S10). At ≤4.5 vol % water in DMSO electrolyte, mixtures of all three benzylic toluene oxidation products were observed (Figures S7, S11, and S12).

Our findings indicate that water is not only a reactant in water oxidation and an O atom and proton donor in our system, but also stabilizes the ^·^OH radical (Figure S28) and transient methyl aryl oxidation radicals (Figure S30) within the wet [H^+^-DMF/DMF] solvent cluster. The reported free energy for proton transfer from water to DMF is +0.6 kcal mol^–1^,^163^ making protonation of DMF feasible. In contrast, proton transfer to acetonitrile is significantly less favorable (+14 kcal mol^–1^),^163^ helping to explain the lack of selectivity observed in acetonitrile (Figures S7 and S14–S16). Although proton transfer from water to DMSO is favorable (−7.4 kcal mol^–1^),^164^ DMSO reacts with ^·^OH to form MSA,^121^ which lowers the toluene oxygenation yield.

### Robustness of Approach across Catalyst Synthetic Methods

While laser-synthesized [Ni_0.76_Fe_0.24_]-(OH)_2_ nanocatalysts performed well in toluene oxidation in wet DMF (Figure 1), control experiments with a similar [Ni_0.75_Fe_0.25_]-(OH)_2_ material (Figure S34), prepared by coprecipitation following a modified published protocol,^165^ yielded the same 100% selectivity for benzyl alcohol in toluene electrooxidation in DMF with 7.0 vol % water (Figure S35), highlighting the universality of our approach. However, the coprecipitated catalyst, which consisted of larger particles (Figures S34 and 1), exhibited only 57% conversion after 48 h, compared to 87% for the laser-synthesized nanocatalyst (Figure S35).

Quantification of the decrease in electrochemical surface area (ECSA) between the coprecipitated and laser-synthesized materials helps explain the observed activity difference between these chemically similar but morphologically different catalysts. EIS data were used to derive double-layer capacitance values on flat highly ordered pyrolytic graphite (HOPG) electrodes (Figure S36) at a catalyst mass loading of 44 μg cm^–2^, which, in the context of EIS measurements on similar anodes, constitutes a thick catalyst film where multiple particles stack and overlap to form a continuous, multiparticle layer.^166^ HOPG as the electrode support and thick-film mass loading were chosen to isolate ECSA differences intrinsic to the catalyst material. In contrast, on hydrophilic carbon fiber paper, which has a real surface area of 468 cm^2^ per geometric cm^2^,^52^ the catalyst mass loading used in all electrocatalysis experiments was only 0.43 μg cm^–2^, making the impedance response dominated by features of particle-size-dependent catalyst–support attachment. This interfacial morphology becomes increasingly important at lower catalyst loading, where no continuous catalyst layer exists, thereby complicating the extraction of double-layer capacitance values^167^ and, as a result, obfuscating catalyst-to-catalyst comparisons.

We found that the ECSA of the coprecipitated [NiFe]-(OH)_2_ catalyst was lower by a factor of 9.9 ± 0.1 compared to the laser-synthesized [NiFe]-(OH)_2_ nanocatalyst (Figure S36). Since the reaction occurs within the catalyst microenvironment (see above), the reduced activity observed for the coprecipitated material is consistent with its lower ECSA. These findings suggest that while particle size, and consequently ECSA, affects reaction kinetics, selectivity is governed by the choice of electrolyte, underscoring the importance of systems-level design in electrosynthesis and the broad applicability of our approach. The synthetic scheme, which oxygenates toluene via ^·^OH radicals, is only possible in solvents that stabilize radicals, such as wet DMF and DMSO, with a crucial role of water, and only within the anode microenvironment that facilitates reactions between ^·^OH radicals and both toluene molecules and benzyl radical intermediates.

Taken together, our combined experimental and computational results on toluene electrooxidation in wet DMF or DMSO electrolytes establish the mechanistic underpinnings for the observed 100% selectivity for benzyl alcohol and remarkably high toluene conversion yield of 87% in wet (7.0 vol % water) DMF electrolyte. Next, we sought to determine whether a similar strategy—arresting oxygenation after the first step through wet solvent interactions with the benzylic oxygenate via hydrogen bonding networks—would also apply when using benzyl alcohol or benzaldehyde as reactants. Benzaldehyde is a key compound in the chemical industry, widely used in pharmaceuticals, plastic additives, personal care products, and as a flavoring agent in the food and beverage industry. It is also utilized in the production of dyes, coatings, and textiles.^168,169^

### Electrooxidation of Benzyl Alcohol

In addition to providing a molecular level understanding of the experimental observations on toluene electrooxidation to benzyl alcohol, our computations predicted that the electrooxidation of benzyl alcohol selectively produces benzaldehyde, following an analogous pathway that halts the first oxygenation product by wet solvent–solute interactions with the C–H activated benzylic group. Placing benzyl alcohol in wet DMF reorganizes the hydrogen-bonding network and leads to the formation of a benzyl alcohol radical stabilized by 12.3 kcal mol^–1^ in wet [H^+^-DMF/DMF] solvent, which readily converts to benzaldehyde (Figure 3b). Benzaldehyde forms a solvent cluster with wet [H^+^-DMF/DMF] solvent molecules, stabilized by a hydrogen bond at the protonation site of the DMF, with a clustering energy of 21.0 kcal mol^–1^. Further the formation of a benzoic acid radical is energetically uphill by 5.3 kcal mol^–1^, suggesting the selective formation of benzaldehyde.

Likewise, the electrooxidation of benzyl alcohol in DMSO follows an analogous pathway, starting with the generation of a benzaldehyde radical and formation of benzaldehyde (Figure 3e). Benzaldehyde is stabilized by the hydroxyl functional group of MSA in the wet [MSA/DMSO] solvent, with a clustering energy of 21.1 kcal mol^–1^. The formation of a benzoic acid radical in the wet [MSA/DMSO] solvent is energetically less favorable by 1.2 kcal mol^–1^. In addition, the aldehyde moiety of benzaldehyde is sterically hindered by the surrounding solvent cage (Figure S32f), which prevents further oxidation. Therefore, we predict that the electrooxidation of benzyl alcohol selectively produces benzaldehyde in wet [MSA/DMSO] solvent as well. A molecular representation of the single oxygenation of benzyl alcohol to benzaldehyde is shown in Figure 3h.

Experimental data on the electrooxidation of benzyl alcohol at 2.1 V, catalyzed by laser-synthesized [Ni_0.76_Fe_0.24_]-(OH)_2_ nanosheets on hydrophilic carbon fiber paper in LiClO_4_-supported DMSO or DMF electrolyte with 7.0 vol % water, showed complete selectivity for benzaldehyde (Figure S25), again halting the oxygenation after a single step. Benzyl alcohol conversion in wet DMF electrolyte reached 2.4%, which is 36 times lower than that of toluene.

Thermodynamic analysis of cluster formation predicts similar Gibbs free energy changes for the oxygenation of toluene to benzyl alcohol (−12.1 kcal mol^–1^) and for the oxidation of benzyl alcohol to benzaldehyde (−13.8 kcal mol^–1^) in wet DMF. Nevertheless, we experimentally observed a higher conversion yield for toluene oxygenation than for the benzyl alcohol to benzaldehyde transformation, indicating that cluster formation energies alone do not capture the full picture. We therefore also calculated the free energy change associated with protonating the solvent cluster around the respective reaction product (benzyl alcohol or benzaldehyde), to assess product stability in the wet [H^+^-DMF/DMF] solvent clusters, as greater product stability should lead to a higher conversion yield. These DFT calculations show that the thermodynamic driving force for protonating the solvent cluster around benzyl alcohol (Δ*G*_protonation_ = −17.3 kcal mol^–1^) is significantly more favorable than that for protonating the solvent cluster around benzaldehyde (Δ*G*_protonation_ = −8.7 kcal mol^–1^), consistent with our experimental observation of higher conversion of toluene to benzyl alcohol than of benzyl alcohol to benzaldehyde. The agreement between theoretical predictions and experimental results underscores the critical importance of holistically evaluating the thermodynamic driving forces for both the cluster formation of the product relative to the reactant and the solvent protonation around the product in governing reaction efficiency.

### Electrooxidation of Benzaldehyde

We investigated the oxygenation of benzaldehyde to benzoic acid in both solvents by DFT calculations. We placed benzaldehyde in wet DMF and calculated the clustering energy to be 28.7 kcal mol^–1^. Although benzoic acid forms a more stable cluster in wet [H^+^-DMF/DMF] solvent than benzaldehyde, making this oxygenation step energetically favorable by −8.5 kcal mol^–1^, as illustrated in Figure 3c, we predict that this reaction cannot proceed because the solvent molecules hinder the reactive site of benzaldehyde (Figure S33). Similarly, when benzaldehyde is placed in DMSO, the solvent molecules also block the active site, as displayed in Figure 3f. In wet [MSA/DMSO] solvent, DFT-optimized structures show that benzaldehyde oxygenation to benzoic acid cannot proceed because the solvent molecules hinder access to the reactive site of benzaldehyde (Figure S33). Figure 3i outlines the molecular representation of oxygenation of benzaldehyde to benzoic acid. Experimental electrooxidation of benzaldehyde at 2.1 V did not proceed (Figure S26), consistent with our DFT calculations.

## Conclusions

We developed a novel approach that integrates electrocatalytic water oxidation with toluene oxidation in a single reaction system, utilizing wet DMF or DMSO electrolytes. The organic solvent molecules formed strong hydrogen-bonding interactions with water, effectively preventing O–O bond formation and favoring ^·^OH radical production over hydrogen peroxide and dioxygen formation. The water oxidation was catalyzed by anodes composed of laser-synthesized [Ni_0.76_Fe_0.24_]-(OH)_2_ nanosheets supported on hydrophilic carbon fiber paper. This high-surface-area anode enhanced water oxidation activity, thereby promoting toluene oxidation product formation. Only benzylic C–H activation products—benzyl alcohol, benzaldehyde, and benzoic acid—were observed.

Electrooxidation of toluene achieved 100% selectivity for benzyl alcohol with an 87% conversion yield in DMF electrolyte containing 7.0 vol % water at an uncompensated applied potential of 2.1 V. Analogous experiments in wet DMSO electrolyte also demonstrated 100% selectivity for benzyl alcohol, albeit with a more than 4-fold lower toluene conversion yield. Integrated experimental and computational studies provided a molecular level understanding for the remarkable selectivity observed at this unprecedentedly high conversion yield.

We discovered a new mechanistic pathway in wet DMF electrolyte involving the formation of H^+^-DMF–^·^OH radical complexes, which originate from protons and ^·^OH radicals *in situ* generated by water oxidation. DFT calculations revealed that these radical complexes are stabilized by hydrogen bonding between the radical, protonated and neutral DMF and water, significantly enhancing the EPR-detected formation of organic radicals—likely benzyl radicals—and increasing benzyl alcohol production compared to the reaction in wet DMSO electrolyte. Unlike DMF, DMSO cannot be protonated under the conditions of this study and instead reacts with ^·^OH radicals to form MSA, observed in NMR data, thereby depleting ^·^OH radicals. Notably, selectivity for the singly oxidized product increased at higher applied potentials, suggesting that the greater availability of simultaneously generated protons and ^·^OH radicals directed toluene oxidation toward benzyl alcohol while also improving conversion yield.

Quenching experiments with allyl alcohol, which targeted surface-adsorbed ^·^OH radicals, revealed that the reaction of toluene to benzyl alcohol occurred within the anode microenvironment, where direct reactions between ^·^OH radicals and both toluene and benzyl radicals are facilitated. Thus, in this solid electrocatalyst system, toluene oxygenation operates through a fundamentally different mechanism than radical reactions in solution.

DFT calculations reveal that water not only serves as a reactant in water oxidation and as an O atom and proton donor in our system, but also stabilizes both the ^·^OH radical and transient methyl aryl oxidation radicals within the wet [H^+^-DMF/DMF] solvent cluster, facilitated by hydrogen bonding networks. Further, calculated energies and modeled structures show why further oxidation of toluene is halted after the first oxygenation, resulting in 100% selectivity for benzyl alcohol. Similarly, oxidation of benzyl alcohol (instead of toluene) in wet DMF or DMSO electrolytes was also fully selective for benzaldehyde as the first oxygenation product, albeit with a significantly lower conversion yield than toluene oxidation, explained by computationally determined free energy changes of protonating the product solvation cluster. Hydrogen-bonding-mediated interactions between water and organic solvent molecules stabilize reaction intermediates and products in wet [H^+^-DMF/DMF] and wet [MSA/DMSO] solvents such that the reaction was arrested after the first oxygenation. Meanwhile, benzaldehyde oxidation did not proceed in the wet organic electrolytes, as solvent molecules hindered the reactive site of benzaldehyde. Analogous solvent-mediated interactions are not possible in acetonitrile due to the absence of an oxygen atom, which prevents strong hydrogen bonding. This explains the lack of selectivity in toluene electrooxidation in wet acetonitrile electrolyte.

Overall, our investigations elucidate the mechanistic underpinnings of toluene electrooxidation in wet DMF and DMSO electrolytes, demonstrating how oxidation can be effectively arrested at the benzyl alcohol. The molecular-level insights gained into these complex reaction networks establish fundamental design principles for selective hydrocarbon oxidation by strategically leveraging solvent–mediated interactions in conjunction with water oxidation. Toluene oxygenation via water-oxidation-derived ^·^OH radicals is possible only in solvents that stabilize the radical, such as wet DMF and DMSO, with water playing a key role, and only within the anode microenvironment, which enables reactions between ^·^OH radicals and both toluene and benzyl radicals. Importantly, our finding that electrocatalytic toluene oxygenation in wet organic electrolyte proceeds via a mechanism distinct from bulk radical oxygenation underscores the necessity of systems-level design in electrosynthesis. Our approach and the associated new mechanism pave the way for achieving selective oxidation of hydrocarbons to alcohols without overoxidation, with broad implications for sustainable chemical synthesis, fine and agrochemical production, pharmaceutical applications, biomass valorization, polymer functionalization, conductive polymers, and polymer degradation.^170–175^

## Experimental Section

All chemicals were used as received. Water was deionized by a Thermo Scientific Barnstead Smart2Pure Pro UV/UF 15 LPH Water Purification System and had a resistivity of ≥17.5 MΩ cm. All experiments were performed at room temperature.

### Anode Preparation

The [NiFe]-(OH)_2_ nanosheets were prepared using pulsed laser in liquid synthesis. Details pertaining to this method are provided elsewhere.^38^ Briefly, suspensions of 0.5 g iron (Alfa, −200 mesh, 99+%) powder in a 10 mL solution of 3.0 M nickel nitrate (Alfa, 98%) in pH 10 water were irradiated by a 355 nm, 8 ns pulse laser beam provided by the third harmonic of a 10 Hz Q-switched Nd:YAG laser (Spectra-Physics Quanta-Ray LAB-190) with a pulse energy of 90 mJ. After 60 min of irradiation, the unreacted iron powder was separated from the nanoparticle suspension using a rare-earth magnet. To isolate the nanosheets centrifugation was used. The nanoparticles were washed with five water washes, followed by two acetone (VWR) washes, and then dried under vacuum.

The coprecipitated [NiFe]-(OH)_2_ catalyst was prepared following a modified previously reported method.^165^ A 1.0 M aqueous mixed-metal nitrate solution was made by dissolving 0.92 M Ni­(NO_3_)_2_ and 0.08 M Fe­(NO_3_)_3_ (Thermo Fisher, 98.0–101.0%) in water. Approximately 5 mL of this solution was added dropwise to 150 mL of 2.0 M aqueous NaOH under continuous stirring. The resulting precipitate was collected by centrifugation, washed ten times with water, and then twice with acetone. The final powder was dried under vacuum.

Hydrophilic carbon fiber paper was used as electrode support for the laser made [NiFe]-(OH)_2_ nanosheets. Preparation of hydrophilic carbon fiber paper is detailed elsewhere.^37^ Briefly, as purchased carbon fiber paper (FuelCellStore, AvCarb MGL190) was made hydrophilic by sonicating the carbon fiber paper in aqueous 1.0 M sodium dodecyl sulfate (SDS; AG Scientific, ≥99%) solution for 5 min, followed by a thorough rinse and soak in water to remove any remaining SDS at the surface. Then the carbon fiber paper was electrooxidized in a standard three-electrode cell in aqueous 0.1 M potassium bicarbonate (KHCO_3_, Alfa Aesar, 99.7–100.5%) at +1.63 V vs Ag/AgCl for 20 min.

Hydrophilic carbon fiber paper was cut to make electrodes with dimensions of 7 mm wide by 7 mm long with a 4 mm wide by 28 mm long tab for electrical contact. The active area of the electrode was 0.49 cm^2^. A 100 μL volume of an aqueous catalyst suspension (2 mg mL^–1^) was dispensed onto the hydrophilic carbon fiber paper electrode that was placed on a flat Teflon piece such that the aqueous catalyst suspension covered the same area each time. This was enabled by the hydrophobicity of the Teflon piece causing the aqueous catalyst suspension to form a droplet that wetted the hydrophilic carbon fiber paper above. The catalyst–support composite was dried in ambient air under an infrared heat lamp at 60 °C for 15 min.

### Physical Characterization

Scanning electron microscopy (SEM) images were obtained at UR-Nano, using a Zeiss Auriga scanning electron microscope, equipped with a Schottky field emission emitter, and operated at 20.00 kV with a working distance of 5.1 mm. The [NiFe]-(OH)_2_ on carbon fiber paper electrodes were mounted on aluminum SEM stubs (Ted Pella) with carbon tape (Electron Microscopy Sciences). Energy-dispersive X-ray (EDX) data were collected at UR-Nano with an SEM-integrated EDAX Octane elect plus with silicon drift detector (SDD) spectrometer. The Ni/Fe ratio was determined from EDX data by integration of the Ni and Fe peaks via peak fitting of the background-subtracted signals, with a fit error of ±2 at%.

X-ray diffraction (XRD) data were acquired on a Rigaku XtaLAB Synergy-S diffraction system using Cu Kα radiation (λ = 1.54184 Å) generated by a PhotonJet-S microfocus source at 50 kV, 1 mA. Two combination ω-φ “Gandolfi” scans were performed with a sample-to-detector distance of 34 mm, each for 300 s: (1) ω from −62.00 to 31.00 degrees and φ rotated through 720 degrees, at θ = −42.127 and κ = 70.000 degrees; (2) ω from −31.00 to 61.00 degrees and φ rotated through 720 degrees, at θ = 40.877 and κ = −70.00 degrees. Diffracted radiation was captured by a HyPix-6000HE HPC detector. Powder samples were affixed to a Nylon loop (0.1 mm ID) with a light coating of viscous oil.

### Electrochemical Methods

Electrocatalytic data were collected at room temperature and ambient pressure in a standard one-compartment cell. Two four-channel potentiostats (Admiral Instruments, Squidstat Prime) and an eight-position stir plate (BT Lab Systems, BT1016) were used to carry out electrocatalysis in eight parallel electrocatalysis cells. The electrolyte was 0.1 M LiClO_4_ (BeanTown Chemical) in dimethyl sulfoxide (DMSO; Fisher Chemical), *N*,*N*-dimethylformamide (DMF; Fisher Chemical), or acetonitrile (Fisher Chemical). The electrolyte was stirred at 900 rpm. Toluene (Fisher Chemical) was added to the electrolyte at concentrations of 20 or 2 vol % or 2 μM. Water was added to the electrolyte in concentrations of 2.0, 4.5, or 7.0 vol %. Vertically placed [NiFe]-(OH)_2_–hydrophilic carbon fiber paper working and nickel mesh counter electrodes were used. Chronoamperometry data were collected for 3, 4, 24, or 48 h at non-*iR*-compensated potentials of 0.7, 0.9, 1.1, 1.3, 1.5, 1.7, 1.9, or 2.1 V vs Fc/Fc^+^, using a silver wire pseudoreference electrode that exhibited a potential of 0.4 V vs Fc/Fc^+^ in all electrolytes. Aliquots were taken after specific reaction times as noted in the data and analyzed. Select chronoamperometry experiments at 2.1 V were conducted without substrate or with the substrates benzene, benzyl alcohol, or benzaldehyde (all Sigma-Aldrich).

Cyclic voltammetry (CV) data were obtained in electrolyte solutions containing 5 mM ferrocene, without stirring, by sweeping the potential from −0.2 to 2.5 V vs a silver wire pseudoreference electrode at 50 mV s^–1^ and reported versus the Fc/Fc^+^ couple. CV data were collected using the same electrochemical cell and electrodes as in the chronoamperometry experiments. Additionally, Fc/Fc^+^ CV data were measured in each electrolyte using platinum mesh as both working and counter electrodes.

Electrical impedance spectroscopy (EIS) experiments were performed using an 8-slot BioLogic VSP-3e potentiostat/galvanostat/EIS system. Measurements were conducted in 1.0 M aqueous NaOH at potentials of 0.05, 0.00, and −0.05 V vs the open-circuit potential (OCP), without stirring, in accordance with a reported protocol.^176^ Laser-synthesized or coprecipitated [NiFe]-(OH)_2_ catalysts were deposited on flat, highly ordered pyrolytic graphite (HOPG) electrodes with a geometric area of 0.09 cm^2^, which served as working electrodes, by drop-casting 20 μL of vortexed 2 mg mL^–1^ aqueous catalyst suspensions. The electrodes were then dried in ambient air under an infrared heat lamp at 60 °C for 15 min. A platinum mesh was used as the counter electrode, and a reversible hydrogen electrode (Gaskatel HydroFlex) served as the reference electrode. The sinusoidal perturbation amplitude was 10 mV, and the frequency range was 100 kHz to 100 Hz, with 10 points per decade and each point averaged over five acquisitions. EIS spectra were analyzed using the BioLogic EC-Lab software package.

Electrochemical surface area (ECSA) values were obtained from EIS data, in accordance with the literature.^176^ The double layer capacitance (*C*_DL_) was determined using the following equation:

$$C_{\mathrm{DL}}={\left(Q_{0}{\left(\frac{1}{R_{s}}+\frac{1}{R_{\mathrm{ct}}}\right)}^{a-1}\right)}^{1/a}$$

where *Q*_0_ and *a* are constants that describe the capacitive behavior of the constant phase element (CPE), *R*_s_ is the solution resistance, and *R*_ct_ is the charge transfer resistance arising from residual faradaic processes. These parameters were extracted by fitting the EIS data using the Randles circuit shown in Figure S36. The electrochemical surface area can be calculated from the double-layer capacitance using

$$\mathrm{ECSA}=\frac{C_{\mathrm{DL}}}{C_{s}}$$

where *C*_s_ is the specific capacitance of a smooth, planar catalyst. Since both the laser-synthesized and coprecipitated [NiFe]-(OH)_2_ catalysts are not planar, even though EIS data were collected using flat HOPG electrodes, and the only relevant *C*_s_ value we were able to find is for NiO*_*x*_* (*C*_s_ = 0.040 mF cm^–2^ in 1.0 M aqueous NaOH^176^), we did not derive absolute ECSA values. Moreover, our primary interest was in the ECSA ratio between the laser-synthesized and coprecipitated [NiFe]-(OH)_2_ catalysts, such that the exact value of *C*_s_ cancels and absolute ECSA values are not required for the comparison.

Faradaic efficiencies (FE) were calculated from the moles of product, quantified by NMR analysis, and the total charge passed during the 48-h electrocatalysis experiment, using the following equation:

$$\mathrm{FE}\;(\%)=\frac{\mathrm{moles}\;\mathrm{of}\;\mathrm{product}\cdot n_{e}\cdot F\;(C\;{\mathrm{mol}}^{-1})}{\left|I_{\mathrm{average}}|\;(A)\cdot t\;(s\right)}\times 100$$

where *n*_e_ is the number of electrons required to produce one molecule of product from one molecule of reactant. In our system, the oxidation of toluene in wet DMF or DMSO requires two electrons to form one molecule of benzyl alcohol, so *n*_e_ = 2. *F* is Faraday’s constant. The total charge passed was determined by multiplying the average current (*I*_average_) by the time (*t*) in seconds.

### Product Identification

Nuclear magnetic resonance (NMR) data were obtained with a Varian 400 nuclear magnetic resonance spectrometer. Samples for ^1^H NMR spectra were prepared using 700 μL electrolyte aliquots, each combined with 35 μL 50 mM tetramethyl silane (TMS; BeanTown Chemical) in deuterated acetonitrile (BeanTown Chemical). Using the equation below, concentrations of oxidation products were calculated, using TMS as an internal standard.

$$\left[\mathrm{product}]=\frac{S_{\mathrm{product}}}{S_{\mathrm{TMS}}}\times \frac{N_{\mathrm{TMS}}}{N_{\mathrm{product}}}\times [\mathrm{TMS}\right]$$

where *S*_product_ is the integration of the product signal, *S*_TMS_ is the integration of the internal standard TMS, *N*_TMS_ is the number of protons represented by the TMS peak, and *N*_product_ is the number of protons represent by the product peak. Error bars represent standard deviations from averaging triplicate measurements. From that we derived a relative error of ±7%. The overall selectivity for each product was calculated using the equation below.

$$\%\;\mathrm{Selectivity}=\frac{\left[\mathrm{product}\right]}{\left[\mathrm{total}\;\mathrm{product}\right]}\times 100\%$$

A JEOL ECZL400S 400 MHz spectrometer equipped with a Royal HFX probe was used to acquire ^1^H-DOSY NMR data without sample spinning at 298 K. Samples were prepared using 700 μL of 2 vol % toluene in DMF or DMSO, or 2 vol % toluene in DMF or DMSO with 0.1 M LiClO_4_ and 7 vol % water, each combined with 35 μL of 50 mM TMS in deuterated acetonitrile. The pulse sequence included 24,000 time-domain data points with 16 scans per gradient increment and a delay time of 0.1 s. The gradient strength was incremented linearly in 16 steps from 30 to 300 mT m^–1^ to achieve approximately 90% signal attenuation, balancing sensitivity and resolution to enable reliable extraction of diffusion coefficients. Data processing was performed using Delta 6.2.0.

Electron paramagnetic resonance (EPR) spectra were recorded at 298 or 10 K using a Bruker EMX spectrometer. Data collected at 10 K used the following instrument parameters: center field 3250 G, sweep width 1500 G, sweep time 30 s, time constant 0.01 ms, microwave power 1.00 mW, modulation frequency 100 kHz and modulation amplitude 8.0 G. Data collected at 298 K used the following instrument parameters: center field 3350 G, sweep width 200 G, sweep time 20 s, time constant 0.01 ms, microwave power 1.00 mW, modulation frequency 100 kHz and modulation amplitude 1.0 G. Samples consisted of 0.3 mL aliquots of electrolytes, placed in quartz EPR tubes (Wilmad-Labglass). For spectral measurements requiring a spin trap, 10 mg of 2-methyl-2-nitrosopropane (MNP; Santa Cruz Biotechnology) or N-*tert*-Butyl-α-phenylnitrone (PBN; Cayman Chemical), weighed using a Mettler-Toledo AT201 balance, was added immediately after sample extraction. For 10 K EPR data collection, samples without or with spin traps were immediately frozen in liquid nitrogen. Integrations and linear background subtractions were performed with the Bruker Xenon software. The EPR spectra were simulated using Easy-Spin version 6.0.2.^177^

Gas chromatography (GC) data were obtained using in-line headspace sample direct injection into an SRI, Multi-Gas #5 configuration gas chromatograph. Hydrogen was detected by a thermal conductivity detector, and a flame ionization detector equipped with a methanizer was used to detect all other gases. The electrochemical cell was sealed by O-rings and Teflon tape to ensure it was airtight, evident from the absence of ambient carbon dioxide after electrocatalysis (Figure S19). A certified standard calibration gas (Airgas) was used to calibrate the gas chromatograph.

### Computations

The computational methodology employed in this study began with the generation of initial molecular geometries using Avogadro molecule builder software,^44^ chosen for its robustness in constructing accurate molecular models which are crucial for reliable downstream quantum chemical calculations. Subsequent electronic structure calculations were performed using the ORCA quantum chemistry software package,^45^ well-known for its efficient and accurate handling of DFT computations. Geometry optimizations were conducted using the hybrid functional B3LYP augmented with the D3 version of Grimme’s dispersion with Becke-Johnson damping,^46–48^ and the def2-TZVP basis set^49^ was employed to provide a balance between computational efficiency and the accuracy required for our chemical system. This level of theory is particularly effective for predicting the geometric and electronic properties of molecules, ensuring that the optimized structures are close to their true ground states. Calculated structures were visualized using VESTA.^178^

The conductor-like polarizable continuum model (CPCM)^50^ was utilized to simulate bulk solvation effects, crucial for systems where solvent interactions influence the bulk behavior of the studied molecules. CPCM models the solvent as a polarizable continuum, interacting with the solute to affect its electronic structure and properties, which is essential in our context where solvent effects are non-negligible. We have also added explicit solvent molecules in our calculations to make sure we capture the explicit solvent–solute interactions with the solvent molecules and the reaction intermediates which may be crucial for the reaction pathway. Additionally, clustering energies were calculated using a specific formulation derived from the partition function of the system, providing insights into the energetic favorability of cluster formation under various conditions, which is central to understanding the thermodynamic aspects of our study. Clustering energies were calculated using the formulation:

$$E_{\mathrm{cluster}}=E_{\mathrm{molecule}+\mathrm{solvent}}-[3\times E_{\mathrm{solvent}}+E_{\mathrm{molecule}}+E_{\mathrm{water}}]$$

## Supplementary Material



## Notes

Catalyst characterization and stability data, electrooxidation product generation and radical intermediate data, and calculated structures and energies (PDF)

## Abbreviations

CPCM: conductor-like polarizable continuum solvation model
CV: cyclic voltammetry
DFT: density functional theory
DMF: *N*,*N*-dimethylformamide
DMSO: dimethyl sulfoxide
DOSY: diffusion ordered spectroscopy
ECSA: electrochemical surface area
EDX: energy-dispersive X-ray spectroscopy
EIS: electrochemical impedance spectroscopy
EPR: electron paramagnetic resonance
Fc: ferrocene
GC: gas chromatography
HOPG: highly ordered pyrolytic graphite
*iR*: current · resistance
MNP: 2-methyl-2-nitrosoporpane
MSA: methanesulfinic acid
NMR: nuclear magnetic resonance
OCP: open-circuit potential
PBN: N-*tert*-butyl α-phenyl nitrone
PCET: proton-coupled electron transfer
SEM: scanning electron microscopy
TFSI: bis­(trifluoromethanesulfonyl)­imide
TMS: tetramethyl silane
XRD: X-ray diffraction
ZIR: impedance *iR* determination and compensation technique
