# Supplementary material for: Selective Toluene Electrooxidation to Benzyl Alcohol
Source: J Am Chem Soc. 2025 Sep 24;147(40):36117–35. doi: 10.1021/jacs.5c05986 (PMC12512105; doi:10.1021/jacs.5c05986)
Supplement: Supplementary file 1 [file ja5c05986_si_001.pdf]

Supporting Information

Selective Toluene Electrooxidation to Benzyl Alcohol

Madeleine K. Wilsey[1], Nathalia Cajiao[2,3], Aleksa Radovic[2], Michael L. Neidig[3], Yasemin Basdogan*[1,2,4], Astrid M. Müller*[1,2,4]

[1]Materials Science Program, University of Rochester, Rochester, New York 14627, United States.

[2]Department of Chemistry, University of Rochester, Rochester, New York 14627, United States.

[3]Department of Chemistry, University of Oxford, Inorganic Chemistry Laboratory, South Parks Road, Oxford, OX1 3QR, United Kingdom.

[4]Department of Chemical Engineering, University of Rochester, Rochester, New York 14627, United States.

*Corresponding authors. Email: yaseminbasdogan@che.rochester.edu, astrid.mueller@rochester.edu.

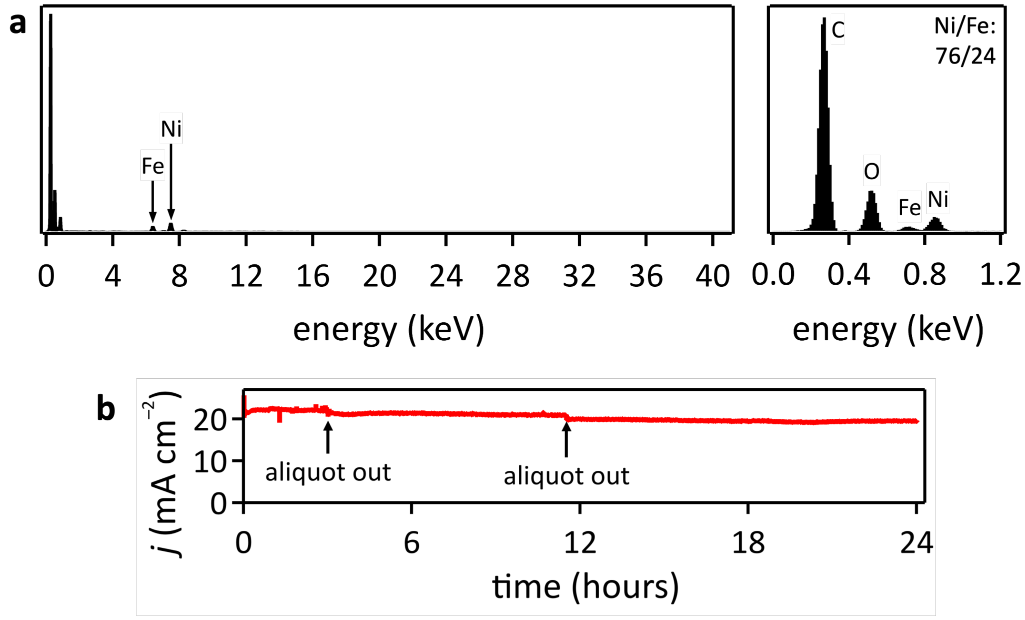

Figure S1. (a) EDX spectra of [NiFe]-$(OH)_2$ on hydrophilic carbon fiber paper, showing the full energy axis (left) and expanded energy axes (right). (b) Chronoamperometric data of electrooxidation at 2.1 V for 24 h of 2.0 vol% toluene in DMF electrolyte with 7.0 vol% water and 0.1 M $LiClO_4$.

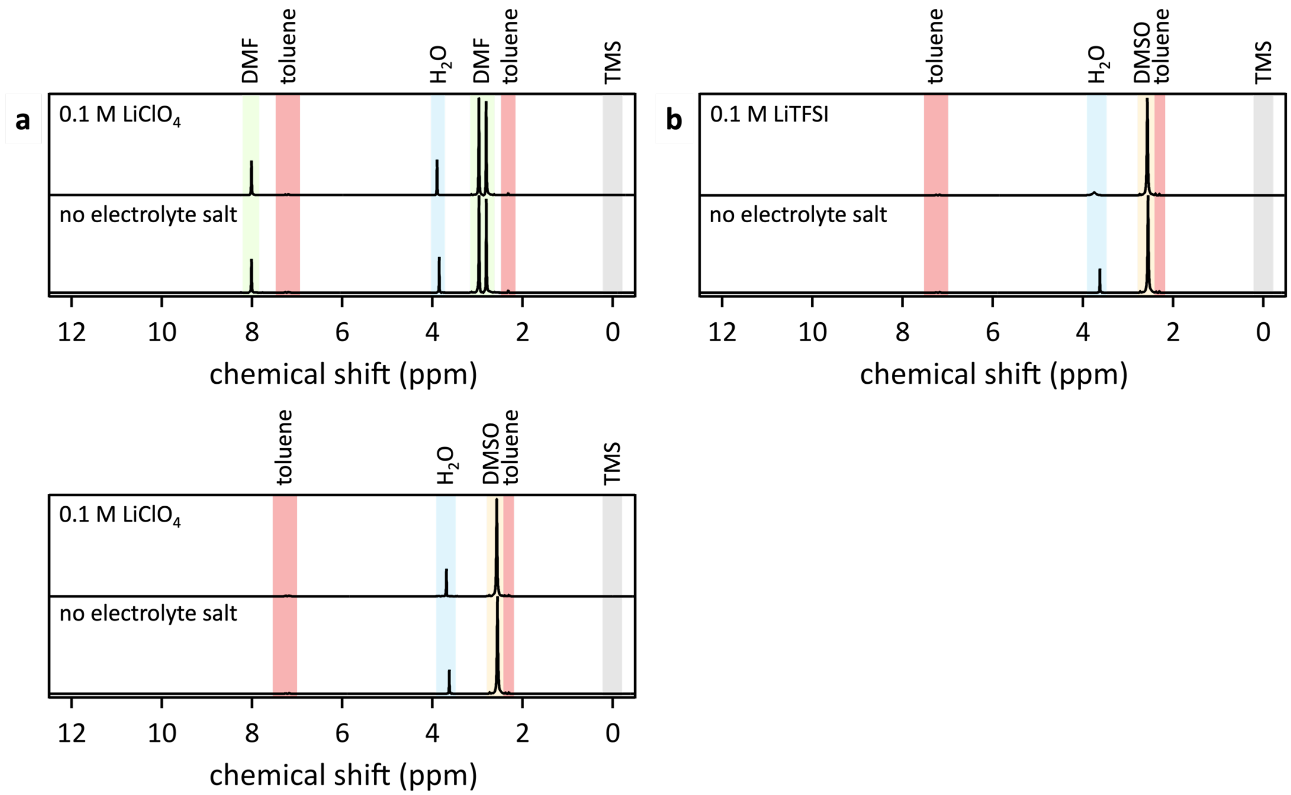

Figure S2. (a) NMR spectra of electrolyte aliquots collected after mixing 7 vol% H_2O, 2 vol% toluene, and DMF (top) or DMSO (bottom) with or without 0.1 M $LiClO_4$. (b) NMR spectra of electrolyte aliquots collected after mixing 7 vol% H_2O, 2 vol% toluene, and DMSO with or without 0.1 M LiTFSI.

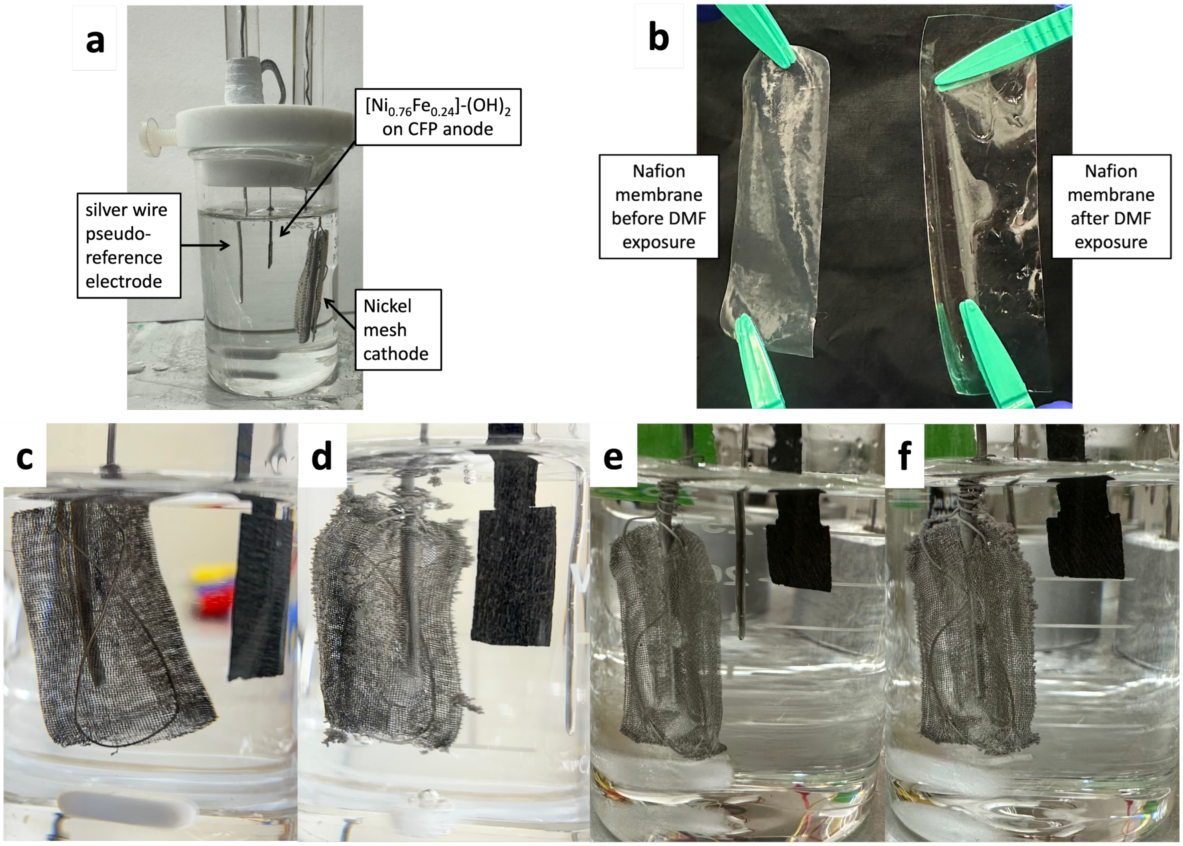

Figure S3. (a) Photograph of undivided cell in which all electrocatalytic experiments were conducted. (b) Photograph of Nafion membrane before (left) and after (right) contact with DMF electrolyte. Photographs of lithium dendrite buildup at the counter electrode in electrolyte with 2.0 vol% toluene and without added water (c) before and (d) after electrocatalysis in DMF electrolyte, (e) before and (f) after electrocatalysis in DMSO electrolyte. All electrolytes contained 0.1 M $LiClO_4$.

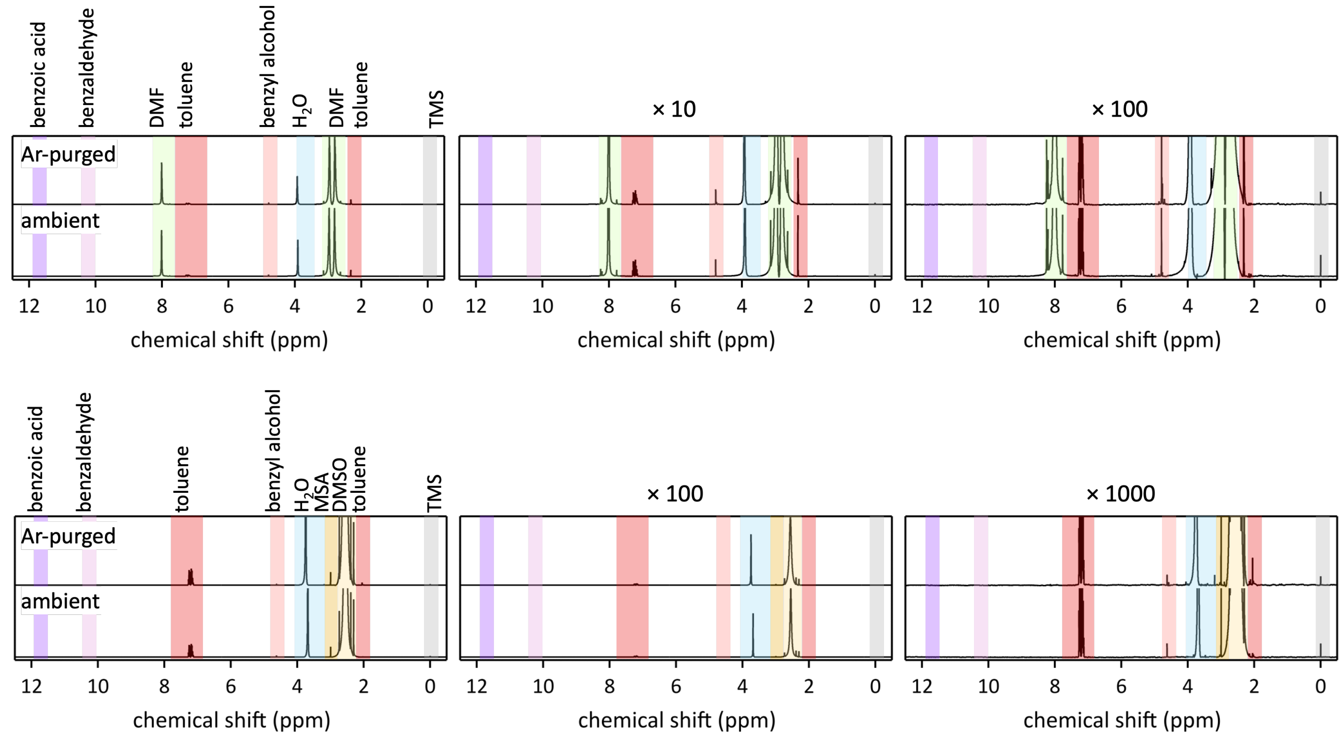

Figure S4. NMR data of electrolyte aliquots collected after 4 h of electrooxidation of 2 vol% toluene at 2.1 V in 0.1 M $LiClO_4$ DMF electrorlyte with 7 vol% water under continuous argon purging (top) or ambient conditions (bottom).

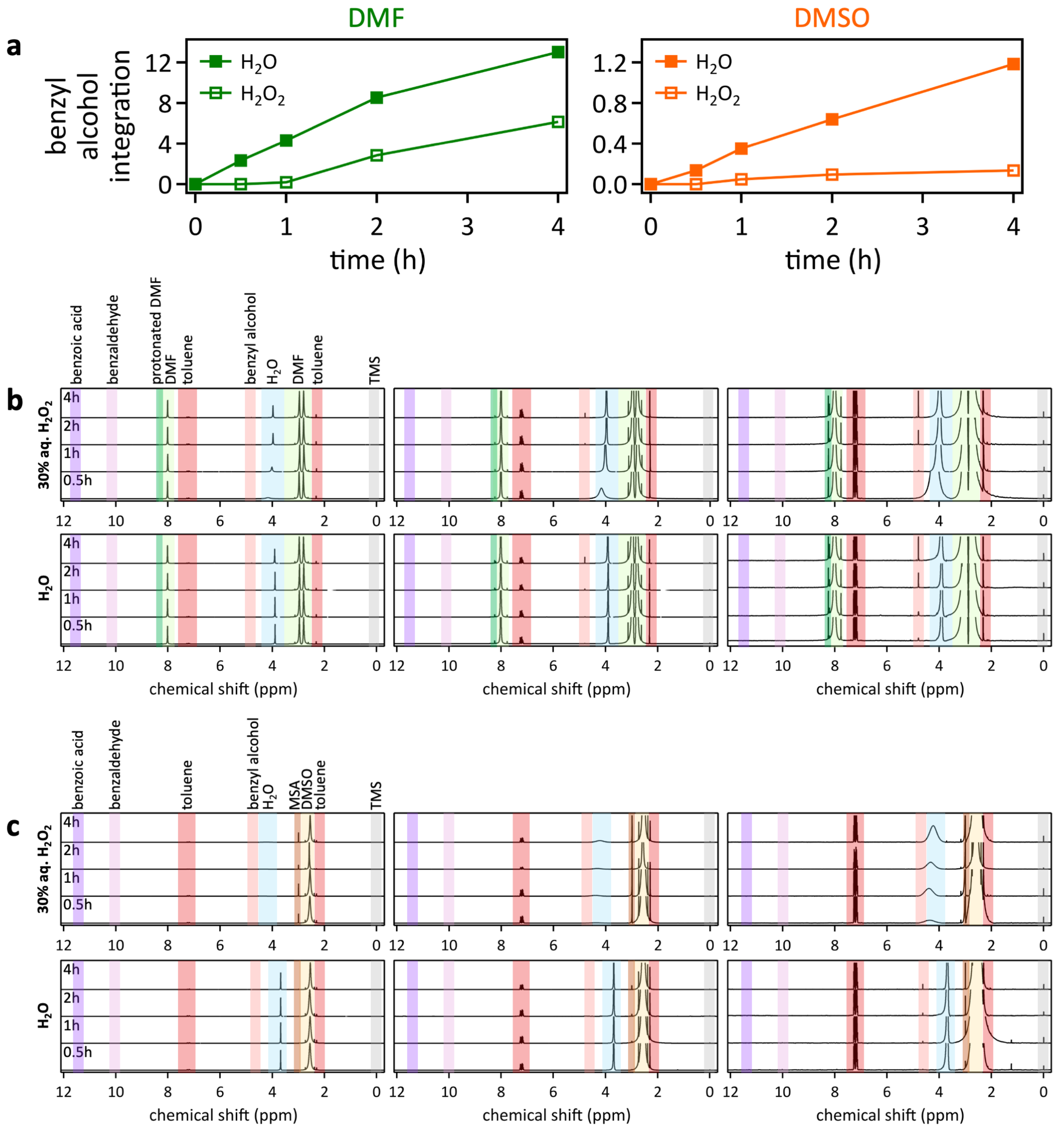

Figure S5. (a) Integrated benzyl alcohol NMR signal of the data shown in (b) and (c). NMR spectra at different reaction times during electrooxidation or 4 h of 2.0 vol% toluene in (b) DMF or (c) DMSO electrolyte with 7.0 vol% of either 30% aqueous H_2O_2 solution (top) or water (bottom). Electrooxidations were performed at 2.1 V in electrolytes with 0.1 M $LiClO_4$.

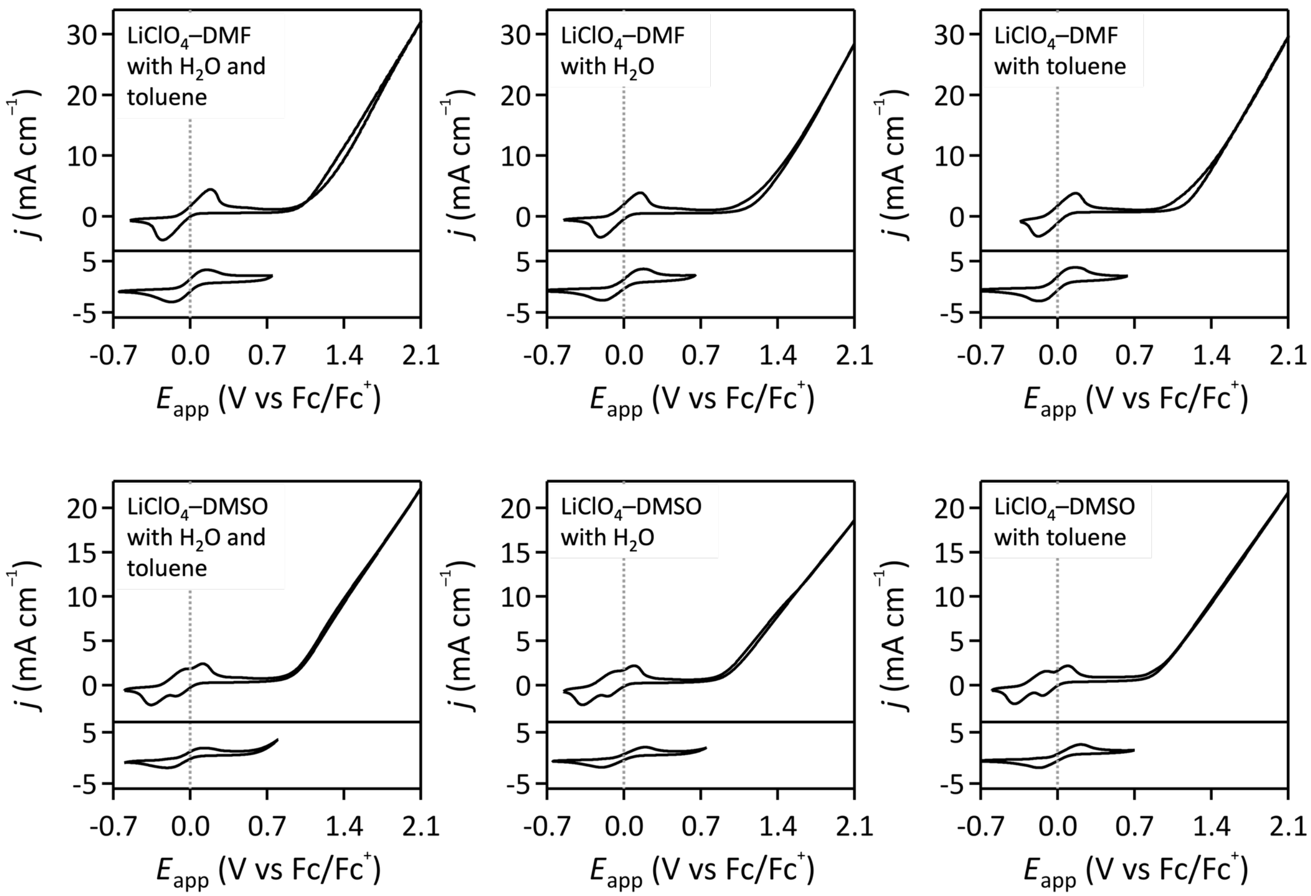

Figure S6. Cyclic voltammograms of toluene in wet DMF or DMSO with 7.0 vol% water and 2 vol% toluene, 7.0 vol% water, or 2 vol% toluene, collected with laser-synthesized [NiFe]-$(OH)_2$ on hydrophilic carbon fiber paper anodes and nickel mesh cathodes (top curves) or platinum mesh anodes and cathodes (bottom curves). The gray line indicates the half-wave potential of the Fc/Fc^+ redox couple, determined for silver wire pseudo-reference electrode in each solution using platinum mesh as both working and counter electrodes.

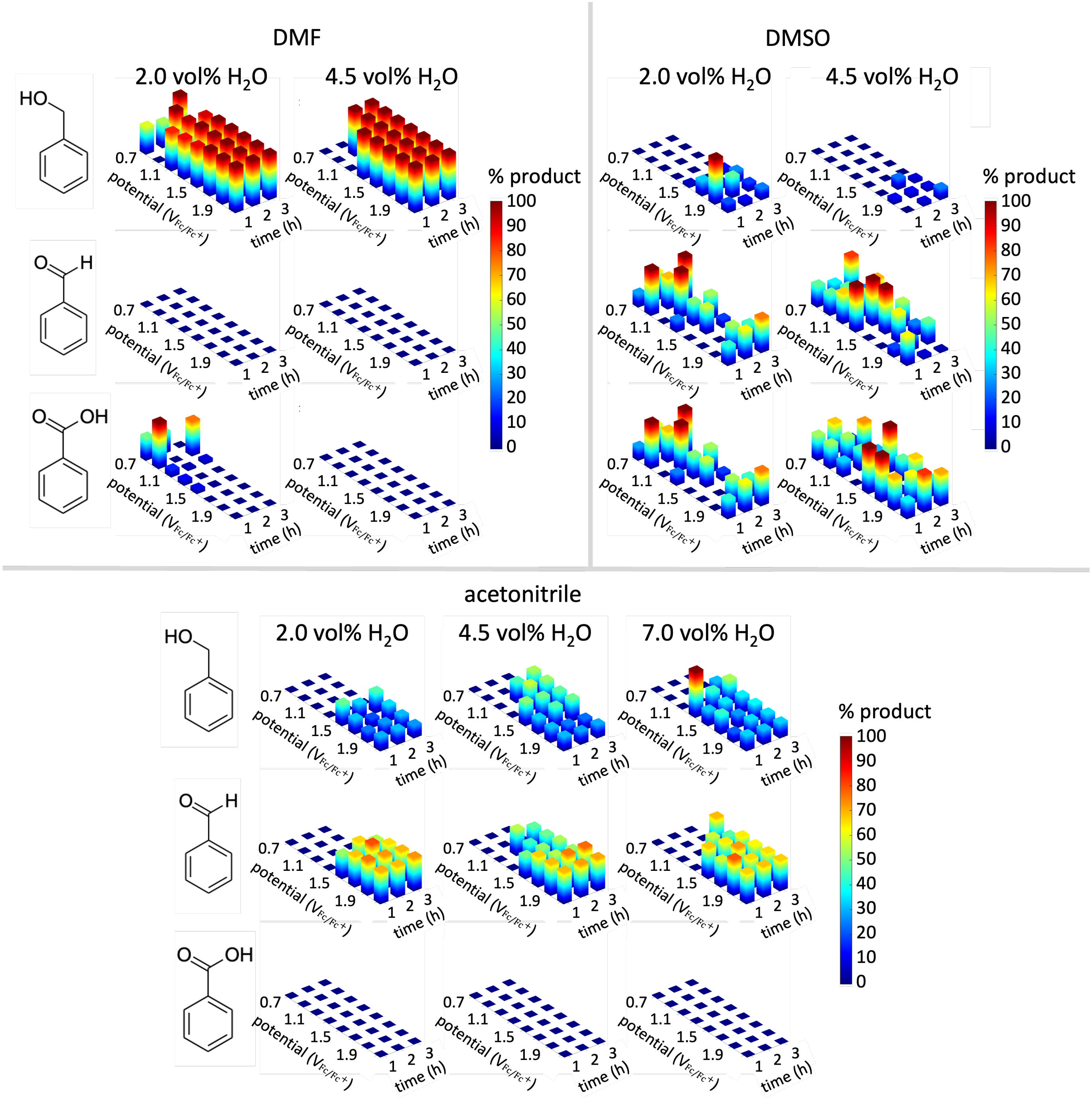

Figure S7. Product selectivity for benzyl alcohol (top), benzaldehyde (middle), and benzoic acid (bottom) derived from NMR data of toluene electrooxidations in DMF (top left), DMSO (top right), or acetonitrile (bottom) with 2.0 vol% or 4.5 vol% water or acetonitrile with 7.0 vol% water at 0.7, 0.9, 1.1, 1.3, 1.5, 1.7, 1.9 or 2.1 V after 1, 2 or 3 h reaction time. All electrolytes contained 20 vol% toluene and 0.1 M $LiClO_4$. Corresponding NMR data are in **Figures S8–S16**.

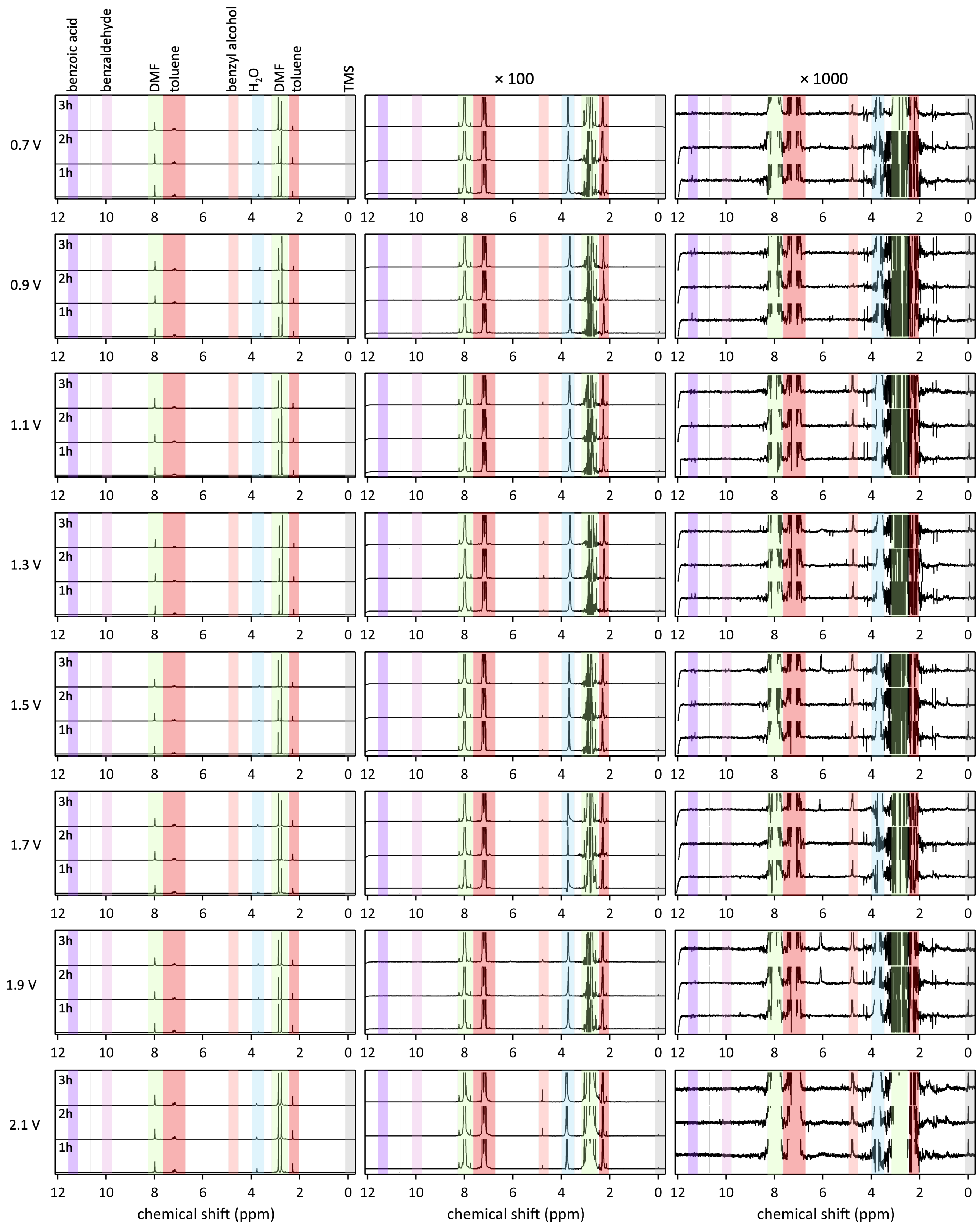

Figure S8. NMR spectra of electrolyte aliquots collected after reaction times of 1, 2 or 3 h of electrooxidation of 20 vol% toluene in DMF with 2.0 vol% water.

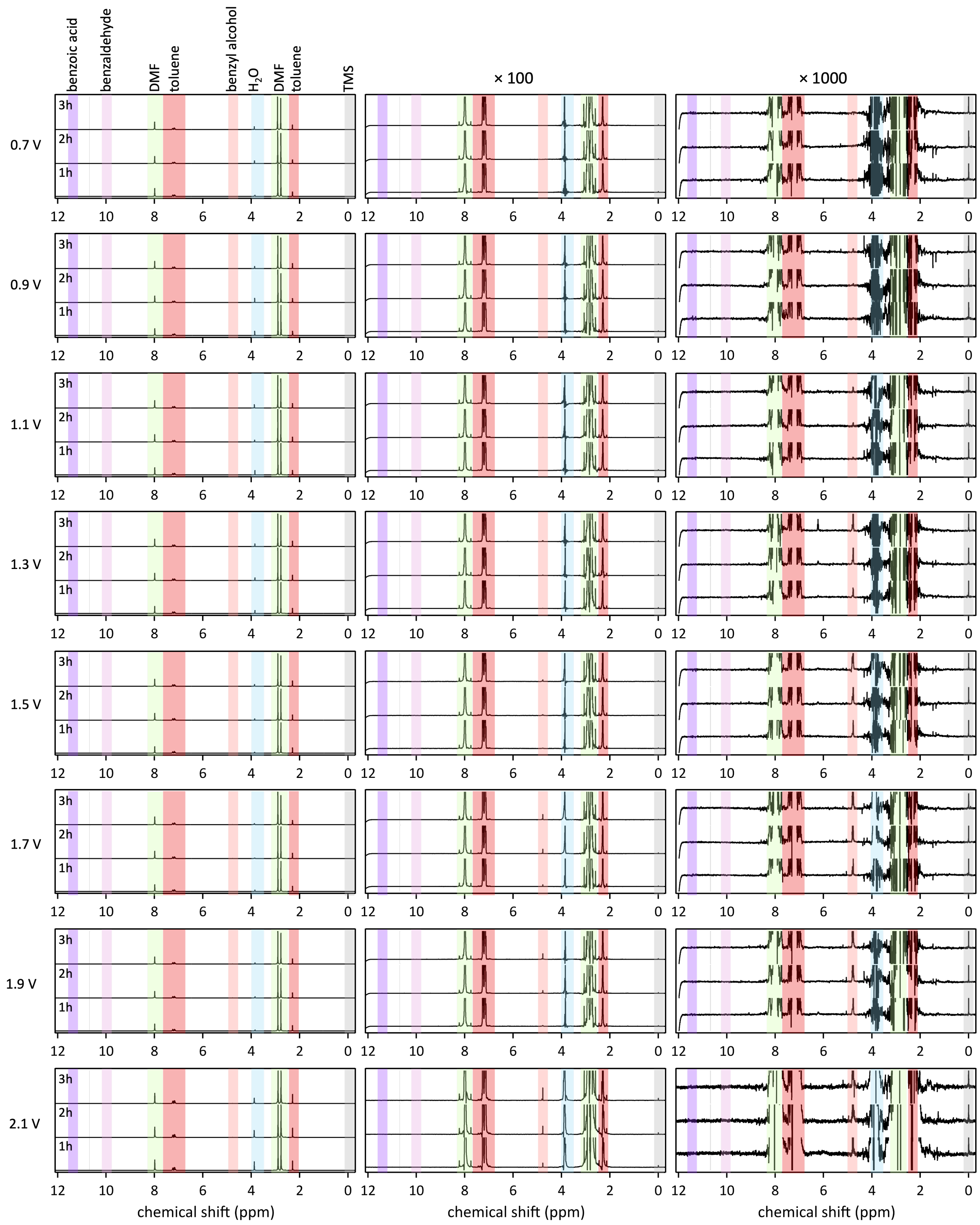

Figure S9. NMR spectra of electrolyte aliquots collected after reaction times of 1, 2 or 3 h of electrooxidation of 20 vol% toluene in DMF with 4.5 vol% water.

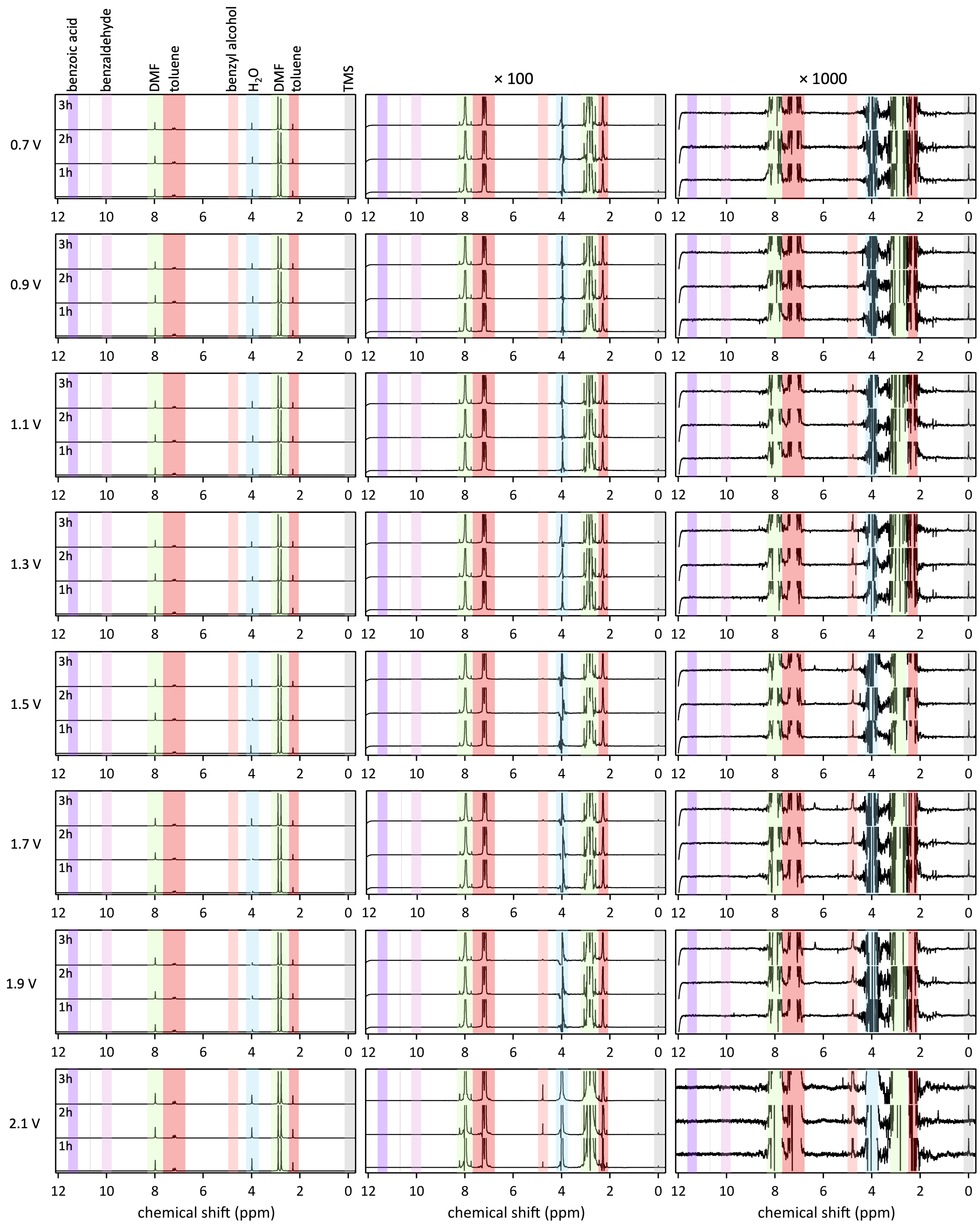

Figure S10. NMR spectra of electrolyte aliquots collected after reaction times of 1, 2 or 3 h of electrooxidation of 20 vol% toluene in DMF with 7.0 vol% water.

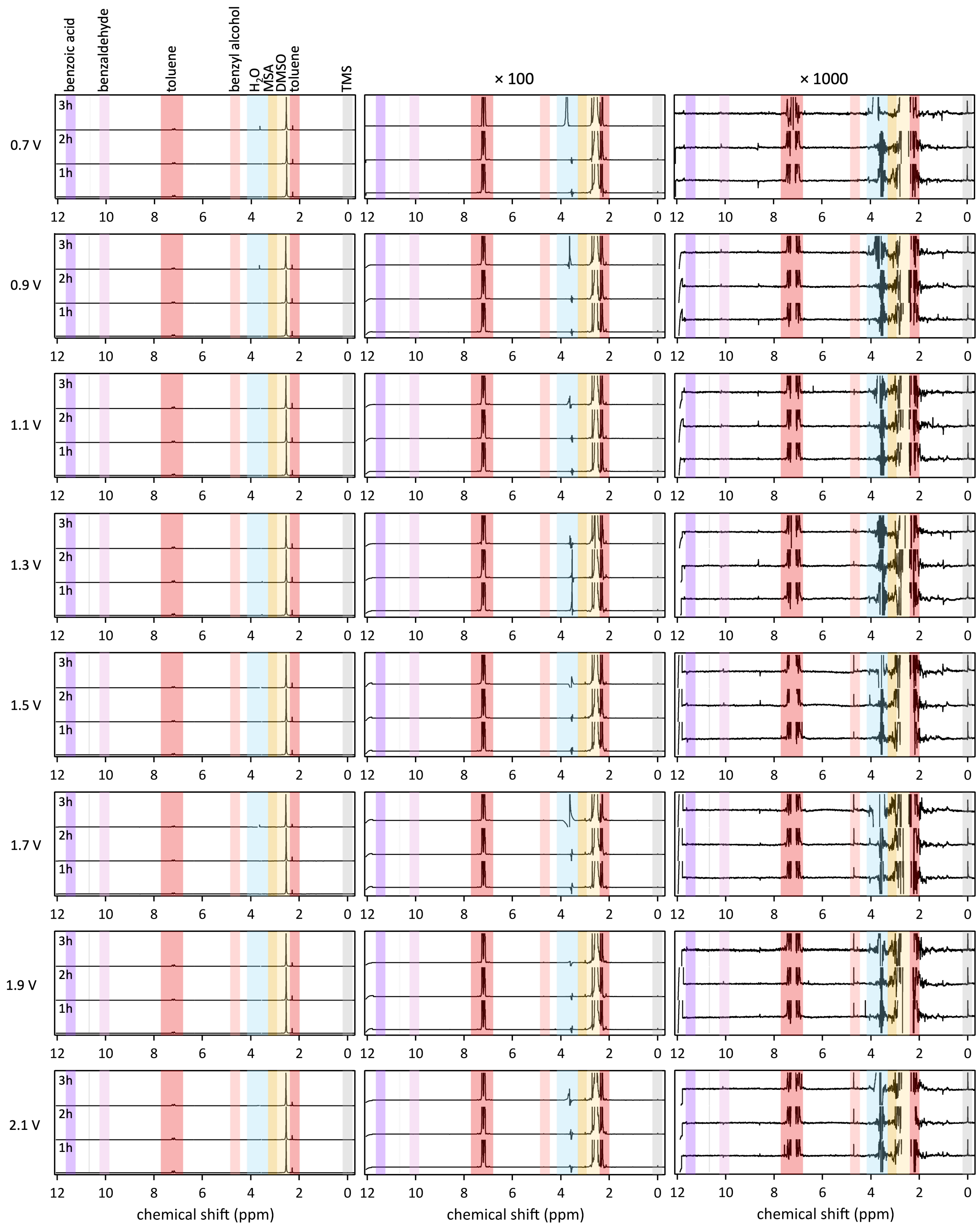

Figure S11. NMR spectra of electrolyte aliquots collected after reaction times of 1, 2 or 3 h of electrooxidation of 20 vol% toluene in DMSO with 2.0 vol% water.

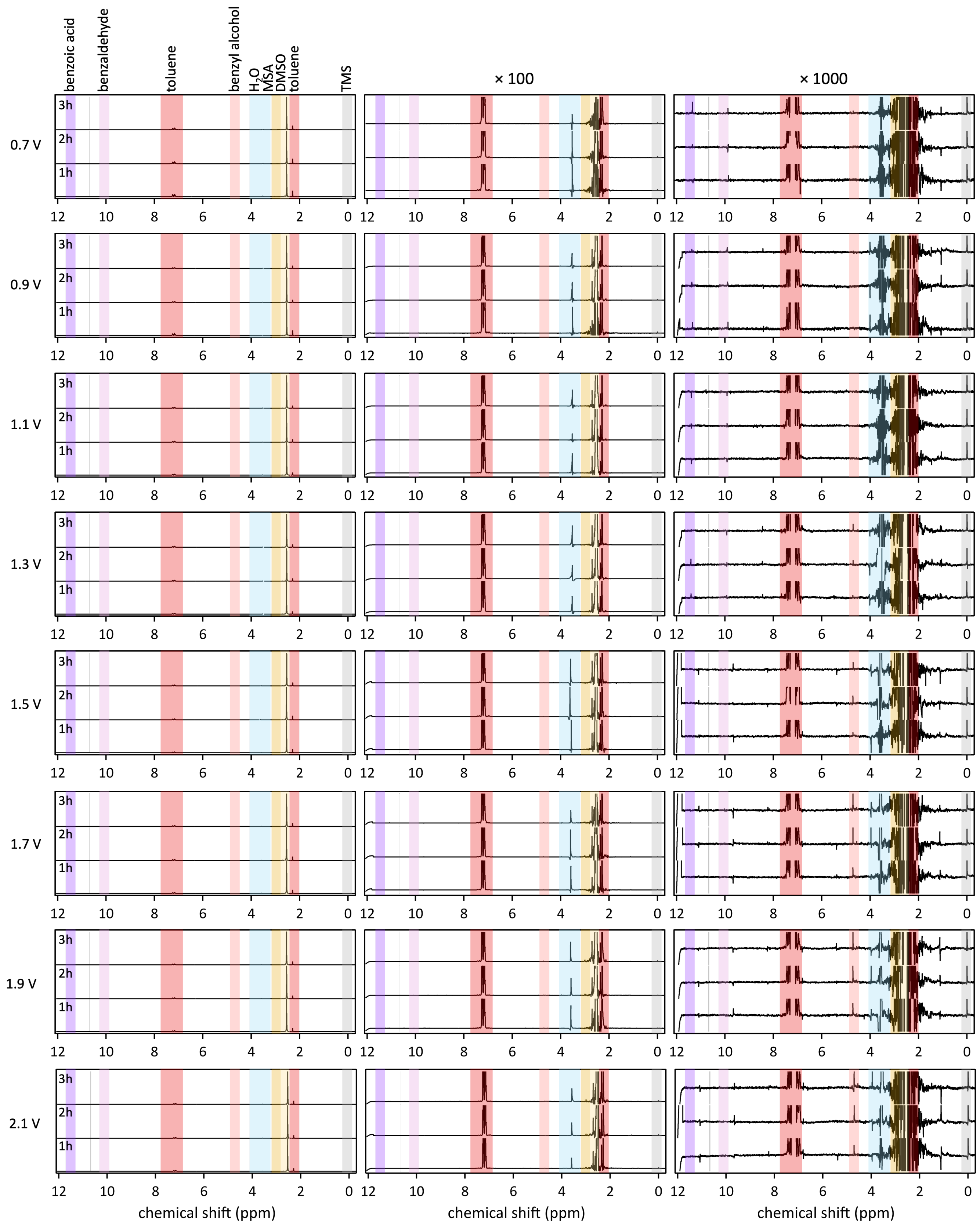

Figure S12. NMR spectra of electrolyte aliquots collected after reaction times of 1, 2 or 3 h of electrooxidation of 20 vol% toluene in DMSO with 4.5 vol% water.

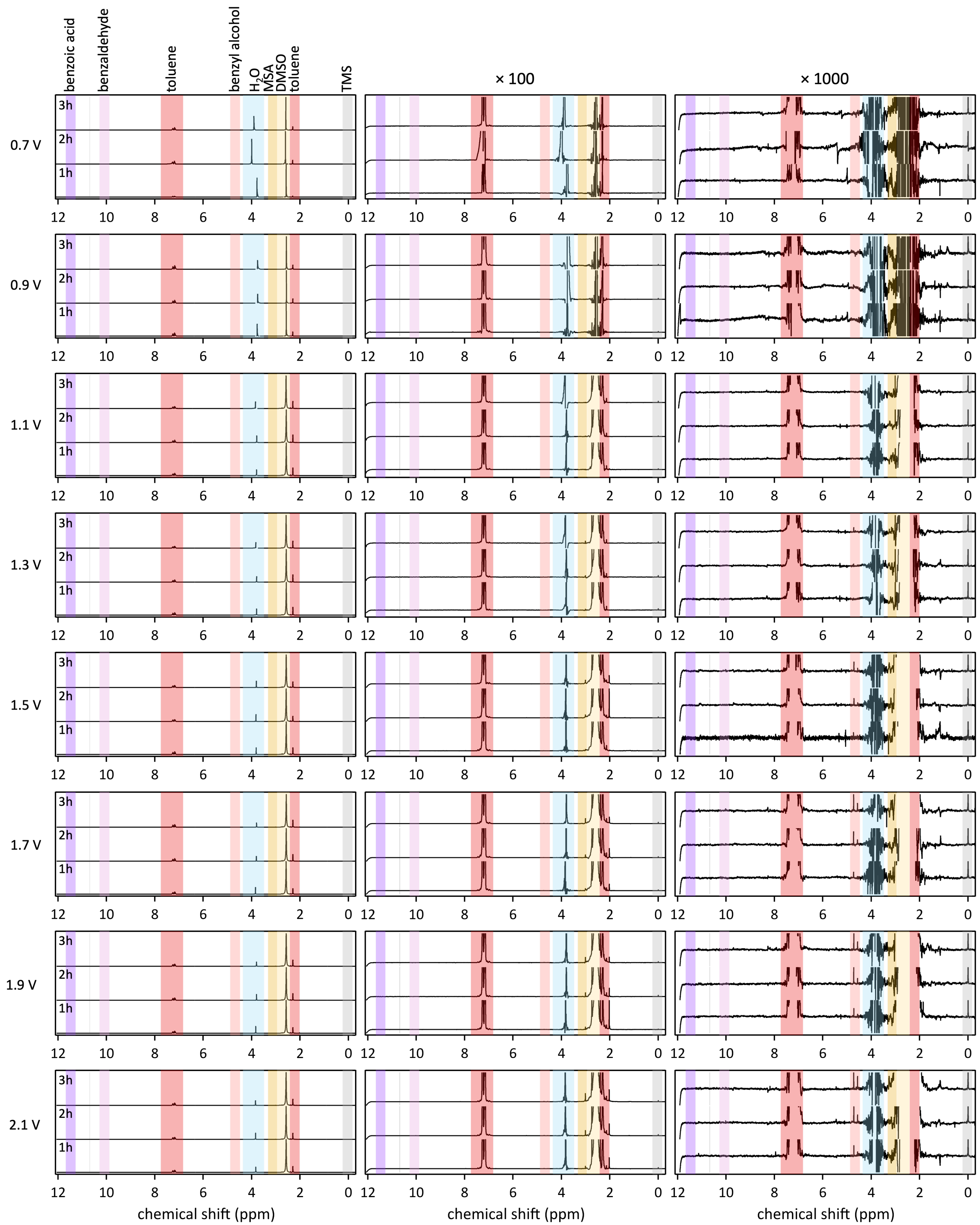

Figure S13. NMR spectra of electrolyte aliquots collected after reaction times of 1, 2 or 3 h of electrooxidation of 20 vol% toluene in DMSO with 7.0 vol% water.

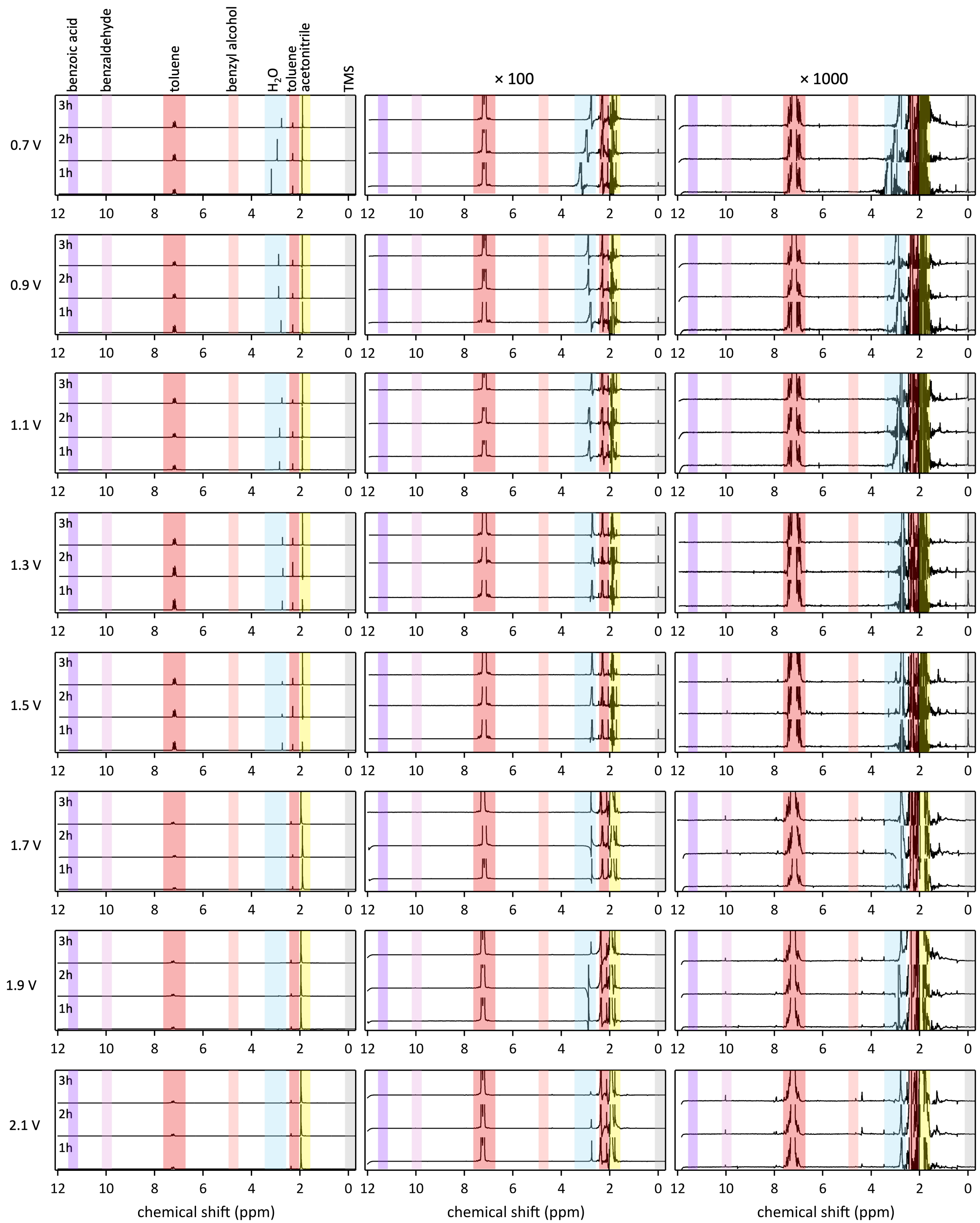

Figure S14. NMR spectra of electrolyte aliquots collected after reaction times of 1, 2 or 3 h of electrooxidation of 20 vol% toluene in acetonitrile with 2.0 vol% water.

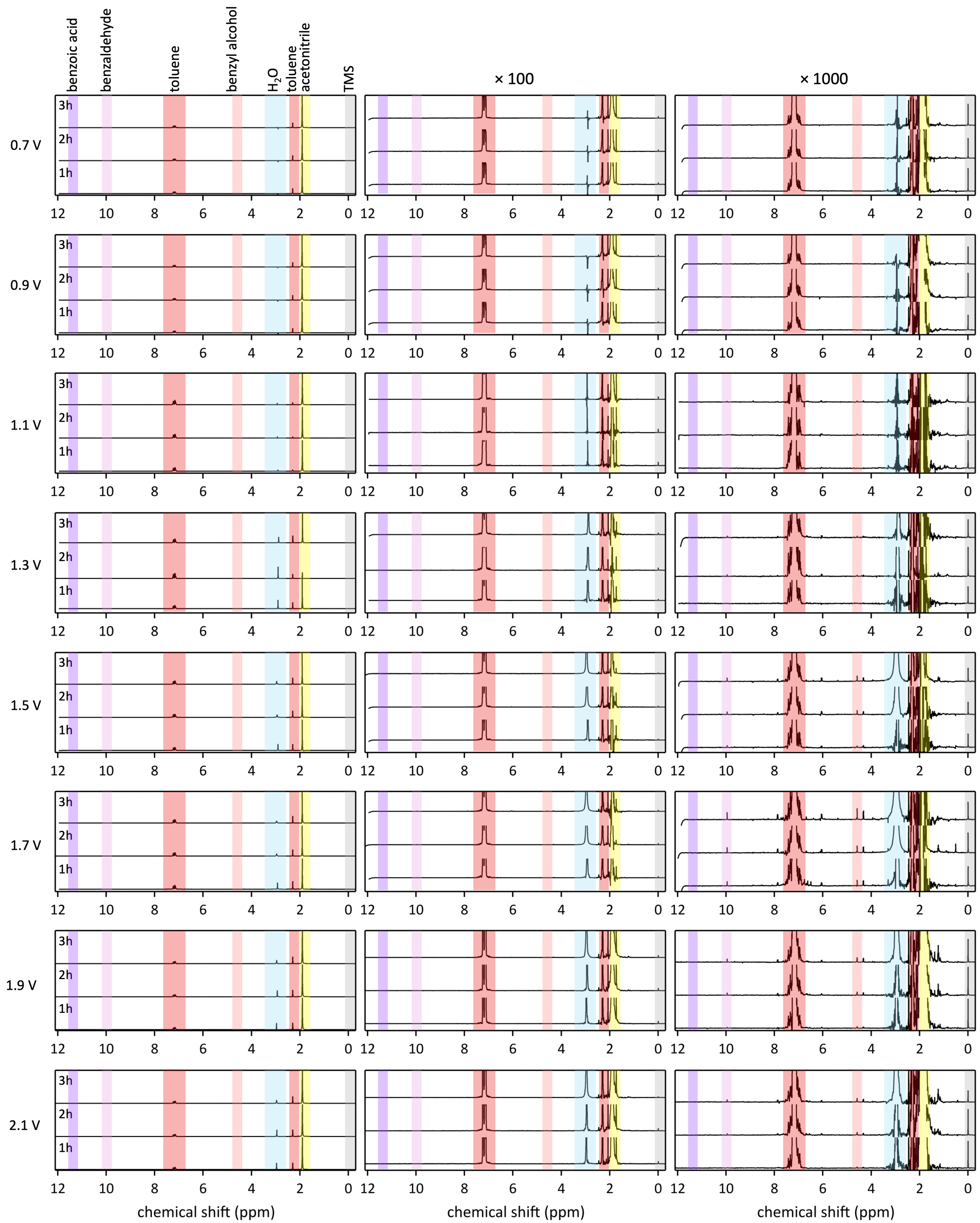

Figure S15. NMR spectra of electrolyte aliquots collected after reaction times of 1, 2 or 3 h of electrooxidation of 20 vol% toluene in acetonitrile with 4.5 vol% water.

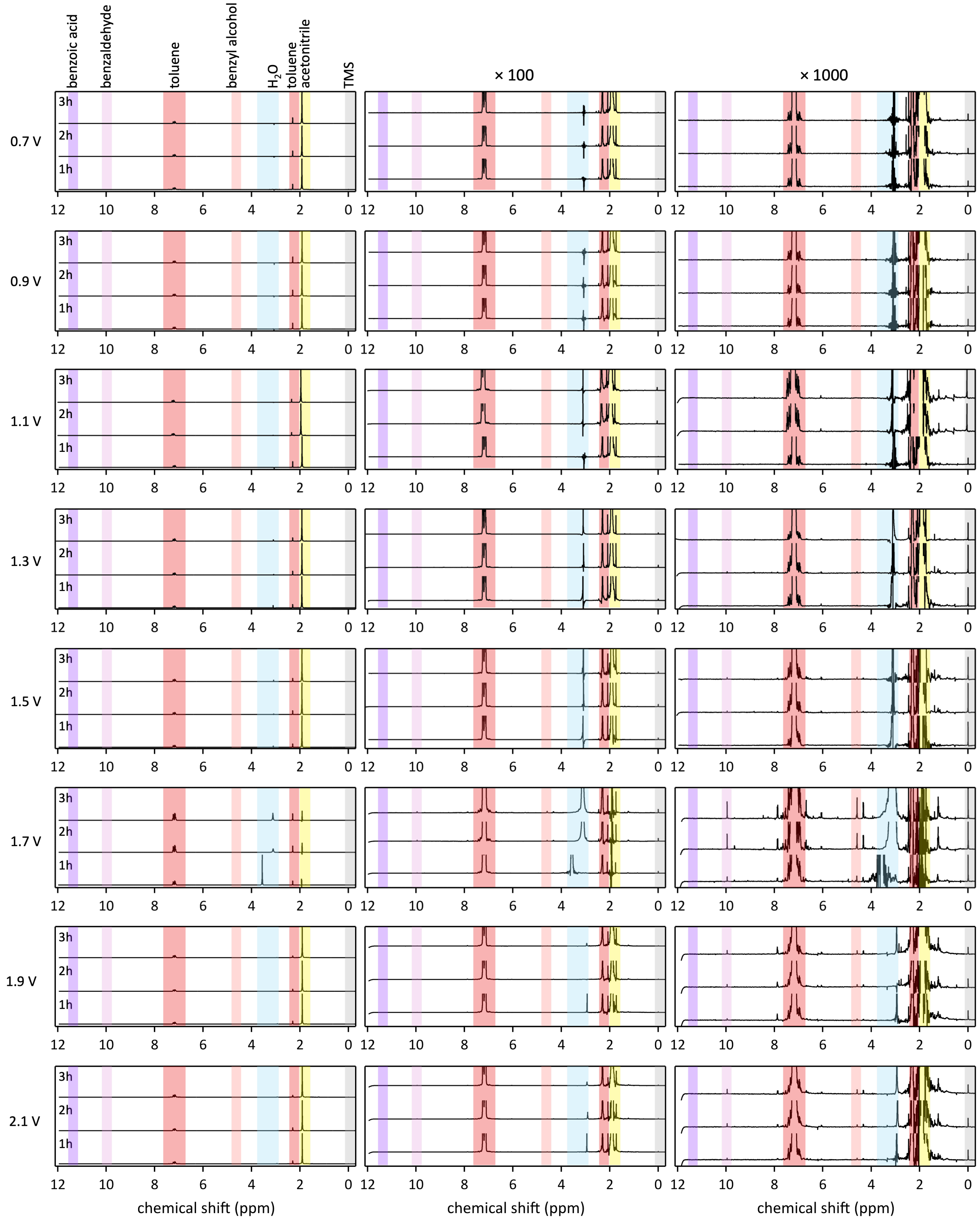

Figure S16. NMR spectra of electrolyte aliquots collected after reaction times of 1, 2 or 3 h of electrooxidation of 20 vol% toluene in acetonitrile with 7.0 vol% water.

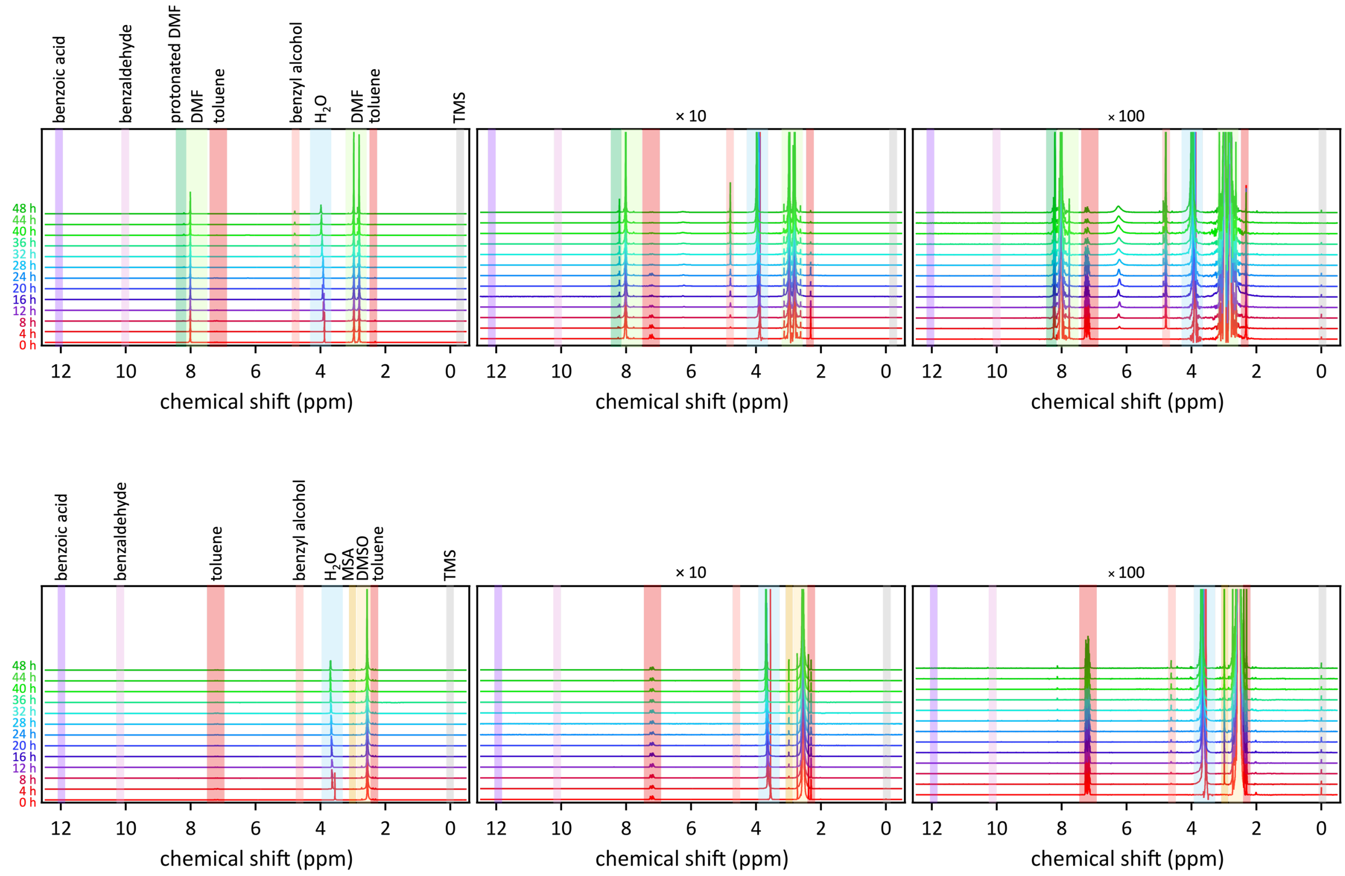

Figure S17. NMR spectra of electrolyte aliquots collected precatalysis and after reaction times of 4, 8, 12, 16, 20, 24, 28, 32, 36, 40, 44, and 48 h of electrooxidation of 2 vol% toluene in DMF (top) and DMSO (bottom) with 7.0 vol% water.

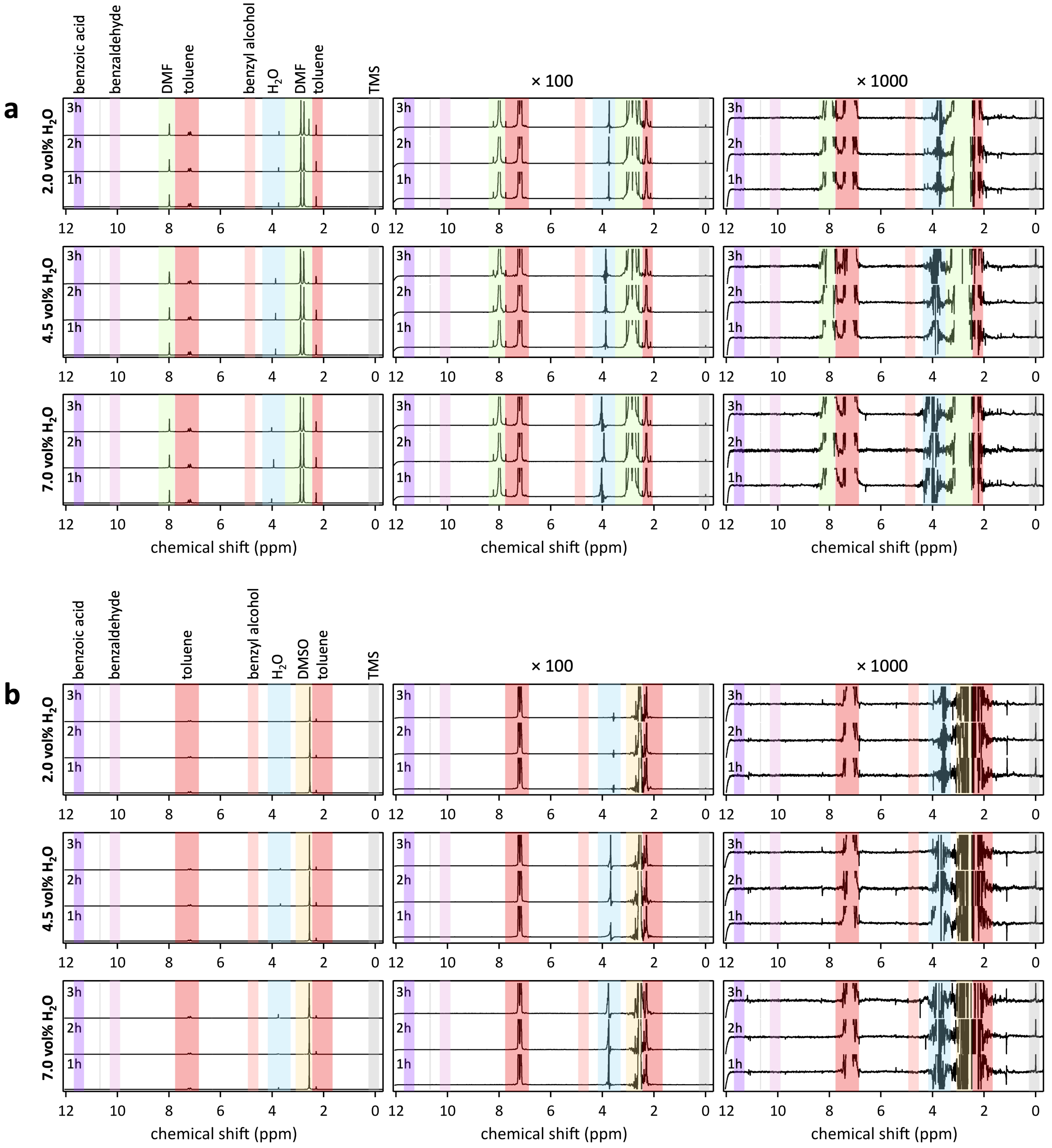

Figure S18. NMR data for toluene electrooxidation at open circuit potential in (a) DMF and (b) DMSO. All electrolytes contained 20 vol% toluene with 0.1 M $LiClO_4$.

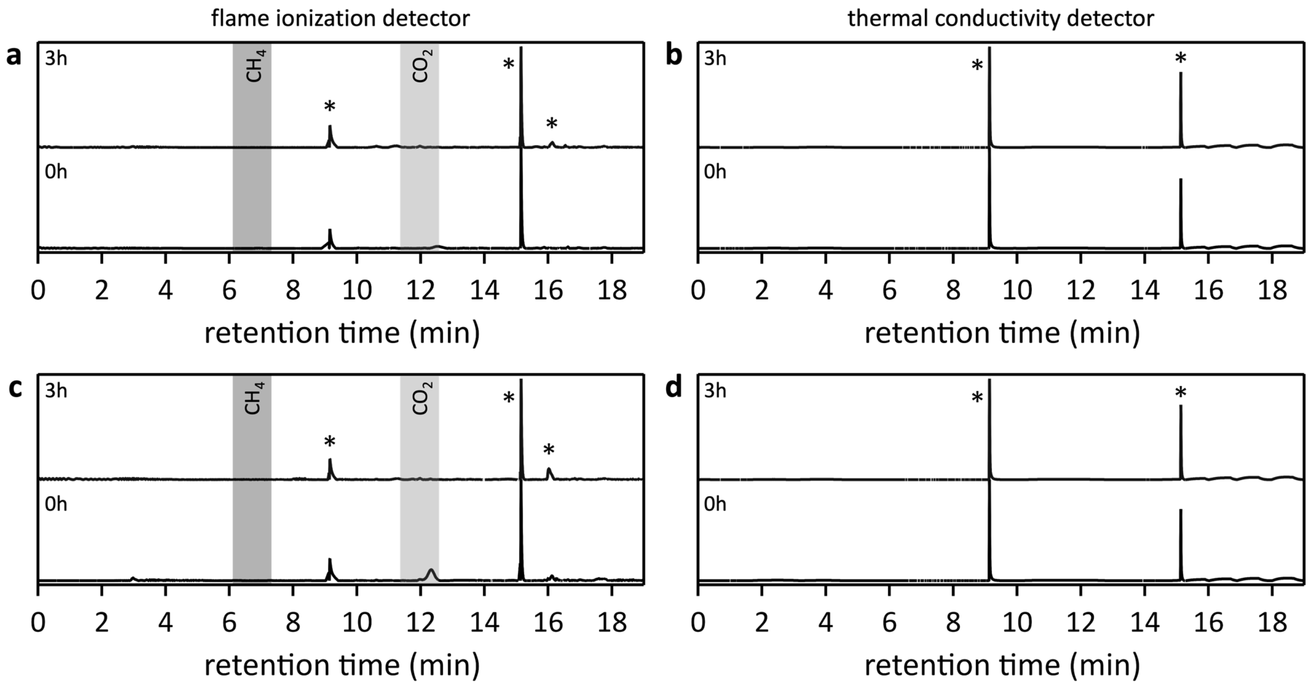

Figure S19. GC data before and after toluene electrocatalysis for 3 h at 2.1 V in (a, b) DMF and (c, d) DMSO. All electrolytes contained 20 vol% toluene, 7.0 vol% water, and 0.1 M $LiClO_4$. Gaseous product regions for methane and carbon dioxide are shaded in dark gray and light gray, respectively. (*) denote signals due to valve changes.

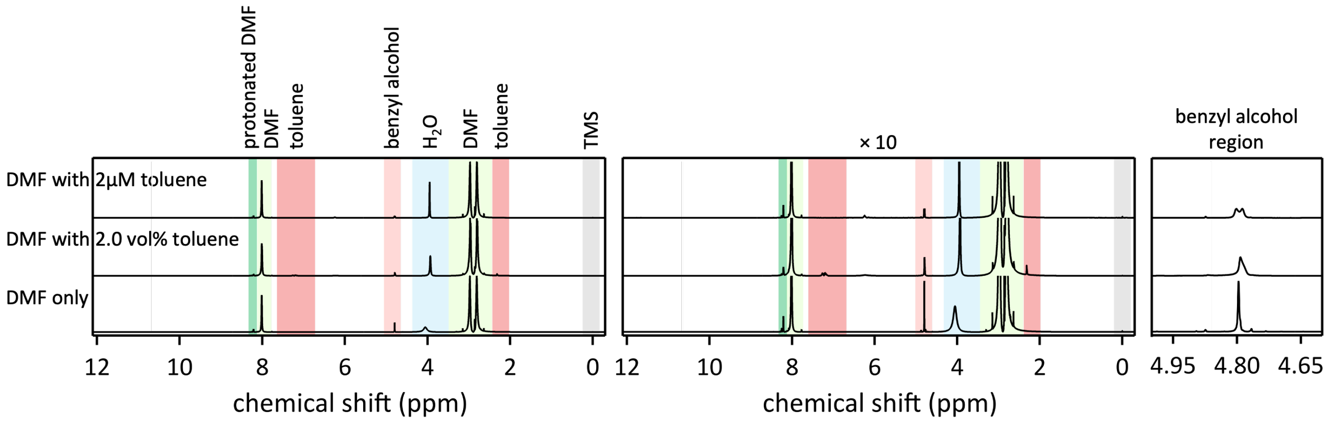

Figure S20. NMR data of electrooxidation for 24 h at 2.1 V of solutions with DMF only, 2.0 vol% toluene in DMF, or 2 μM toluene in DMF. All electrolytes contained 0.1 M $LiClO_4$ and 7.0 vol% water.

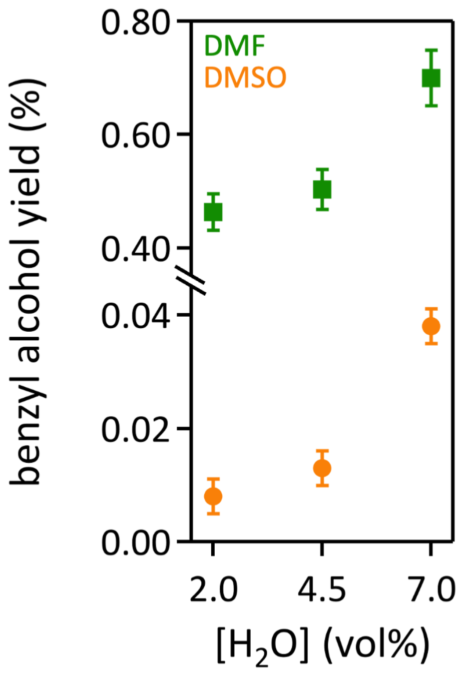

Figure S21. Benzyl alcohol yield derived from NMR data of toluene electrooxidation at 2.1 V for 3 h in wet DMF (green) or DMSO (orange) electrolyte with varying water concentration. All electrolytes contained 20 vol% toluene and 0.1 M $LiClO_4$. Corresponding NMR data are in **Figures S8–S13**.

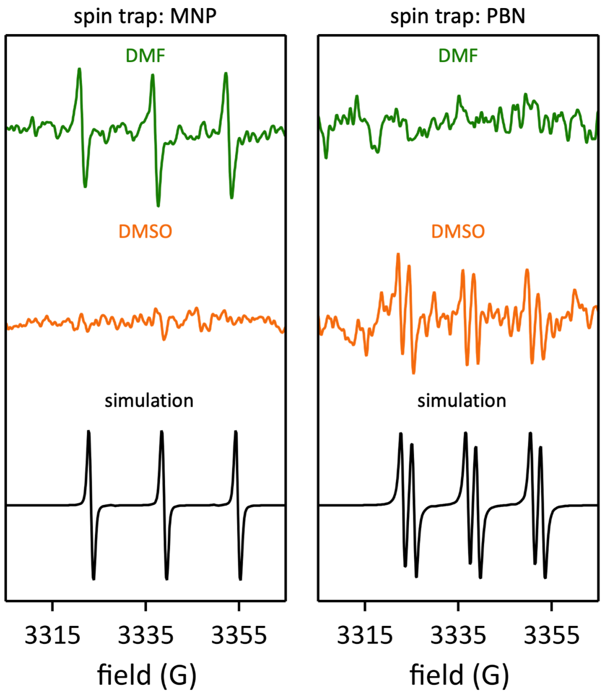

Figure S22. EPR data taken at 298 K after electrooxidation of toluene in wet DMF (green) or DMSO (orange) electrolyte, spin trapped by MNP (left) or PBN (right). Simulations are shown in black, with the corresponding parameters detailed in Tables S1 and S2. Electrooxidations were performed for 24 h at 2.1 V in electrolytes with 2.0 vol% toluene, 7.0 vol% water, and 0.1 M $LiClO_4$.

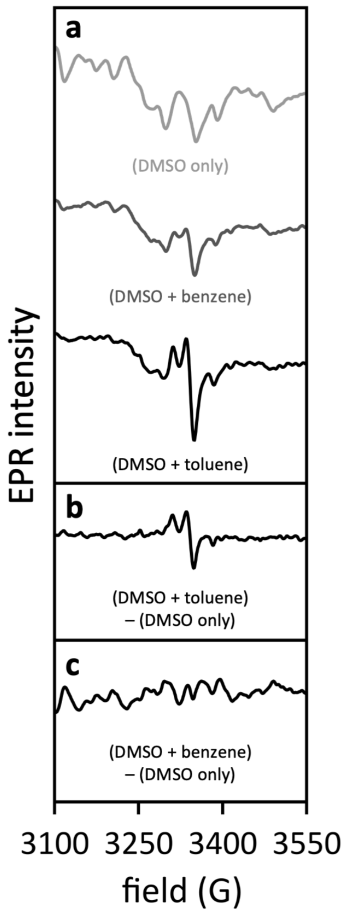

Figure S23. (a) Post electrooxidation EPR data of wet DMSO electrolyte without substrate (light gray), with 2.0 vol% benzene (dark gray), or with 2.0 vol% toluene (black). (b) Difference EPR signal after subtracting EPR data of DMSO electrolyte without substrate from EPR data of electrolyte with toluene. (c) Difference EPR signal after subtracting EPR data of DMSO electrolyte without substrate from EPR data of electrolyte with benzene. Electrooxidations were performed for 24 h at 2.1 V in electrolytes with 0.1 M $LiClO_4$ and 7.0 vol% water. All EPR spectra were spin trapped by MNP, collected at 10 K, and are plotted with identical *y*-axis scaling.

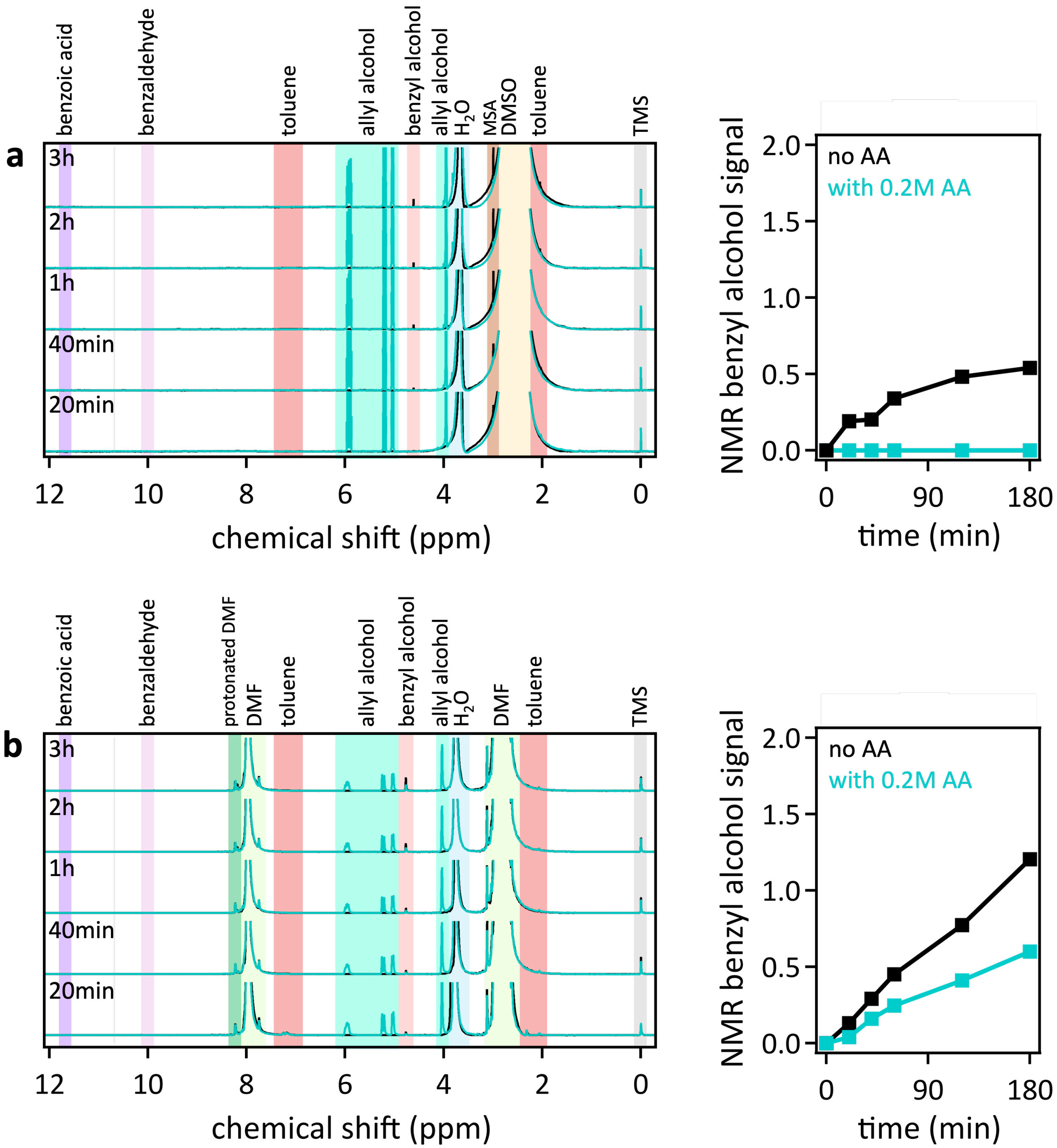

Figure S24. Electrooxidation of 2.0 μM toluene in the presence of 0.2 M allyl alcohol (AA) in (a) DMSO and (b) DMF electrolytes. Overlays of the NMR spectra (left) and benzyl alcohol NMR signal integrations (right) are shown for electrolyte samples with allyl alcohol (teal) and without allyl alcohol (black) at different reaction times. Electrooxidations were performed at 2.1 V for 3 h in electrolytes with 0.1 M $LiClO_4$ and 7.0 vol% water.

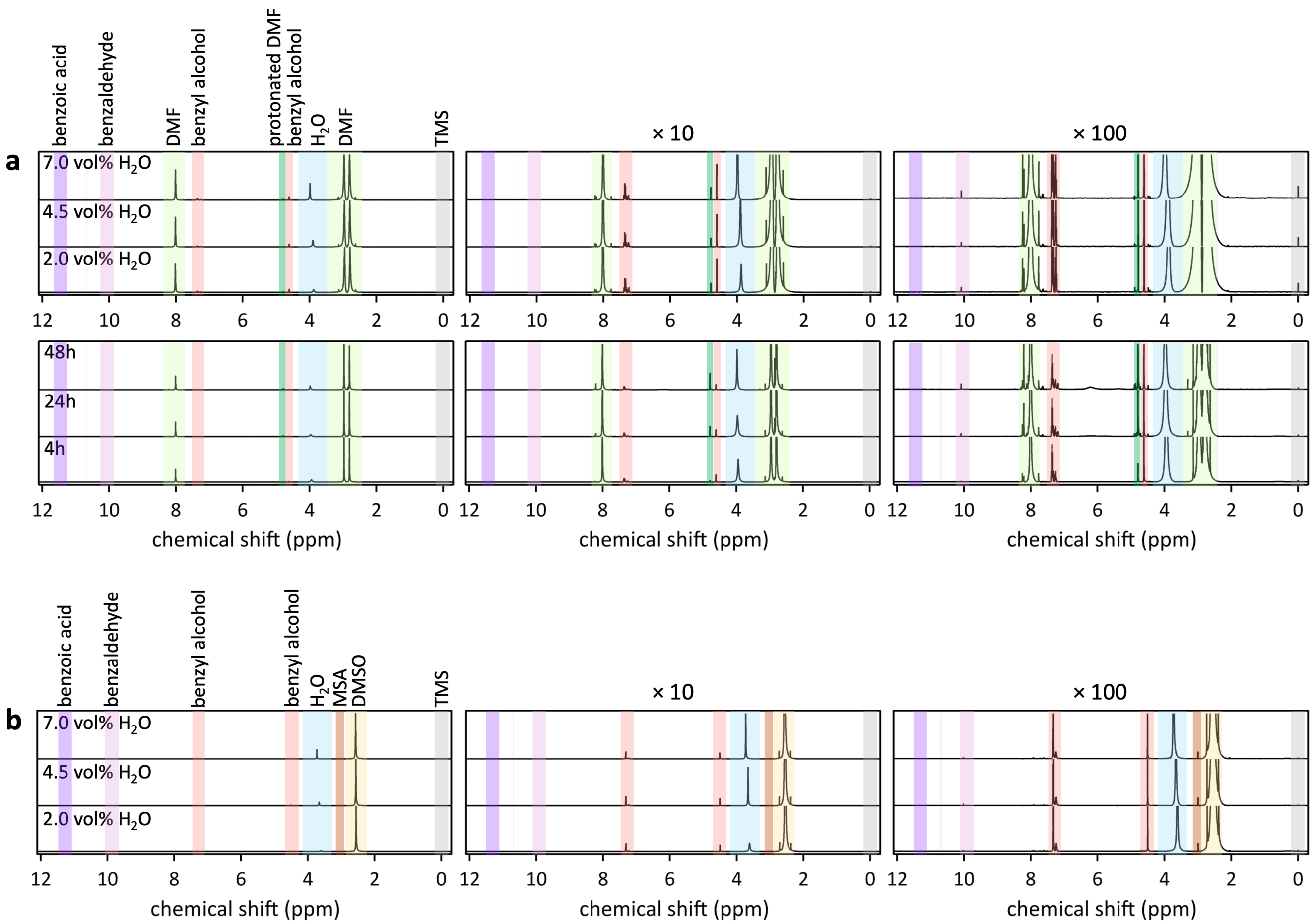

Figure S25. NMR data of electrooxidation of 2.0 vol% benzyl alcohol in (a) DMF electrolyte with varied water concentration for 3 h (top) or with 7.0 vol% water at different reaction times during electrocatalysis for 48 h (bottom), or (b) in DMSO electrolyte with varied water concentration for 3 h. Electrooxidations were performed at 2.1 V in electrolytes with 0.1 M $LiClO_4$.

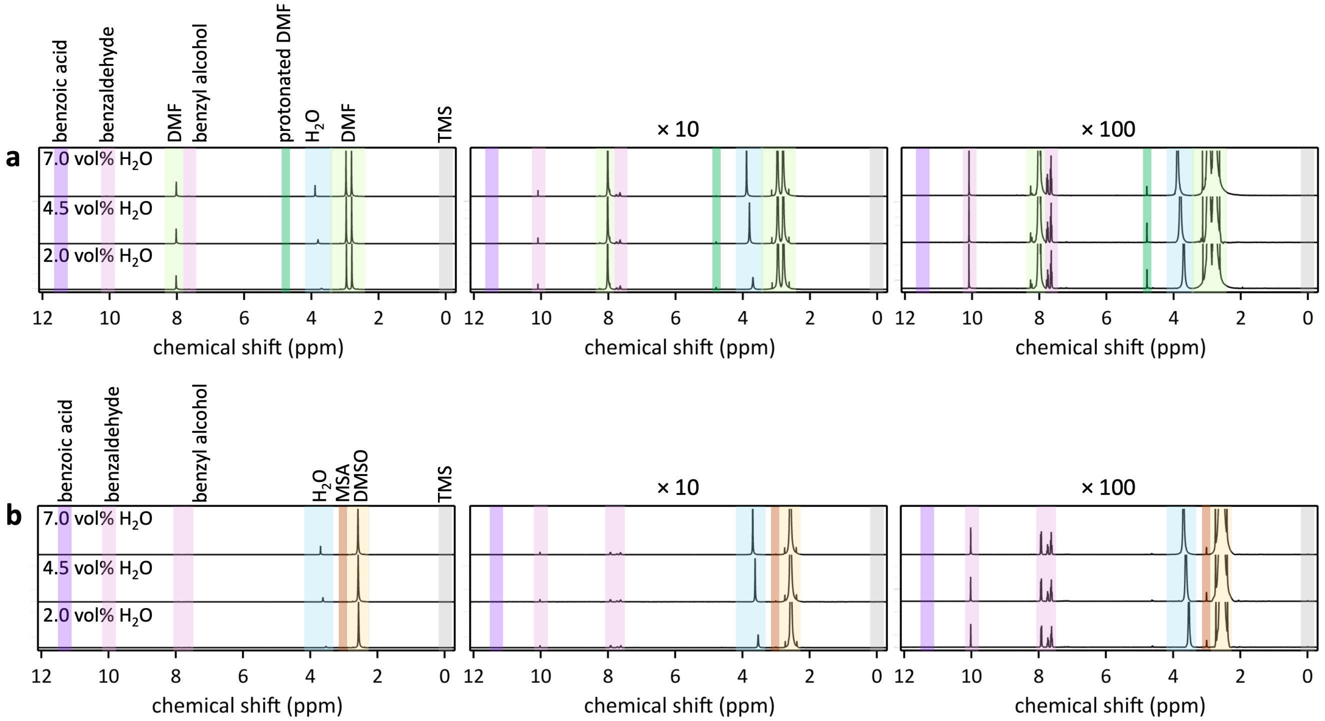

Figure S26. NMR data of electrooxidation for 3 h at 2.1 V of 2.0 vol% benzylaldehyde in (a) DMF or (b) DMSO electrolyte with varied water concentration. Electrolytes contained 0.1 M $LiClO_4$.

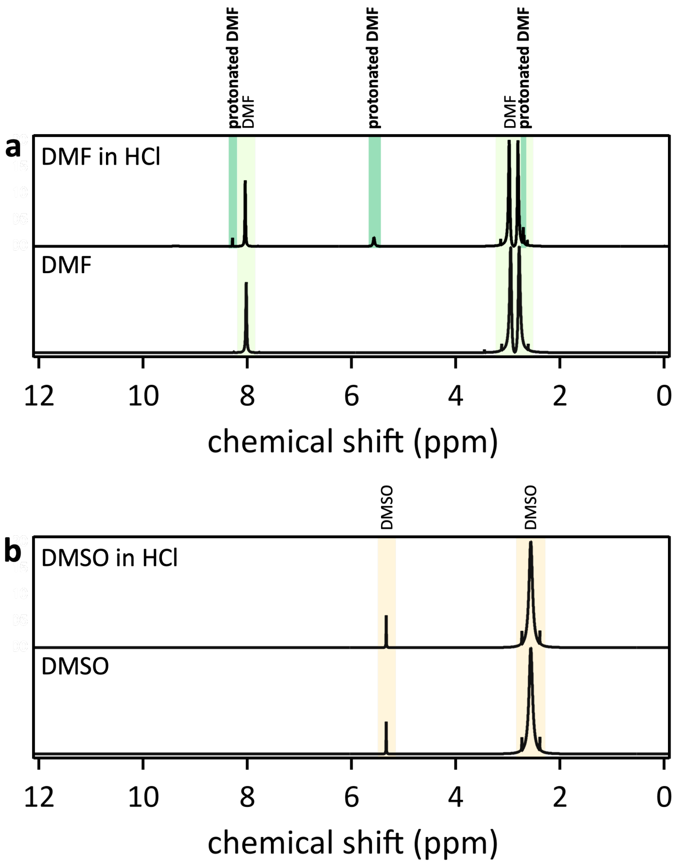

Figure S27. NMR data of (a) 2.0 vol% DMF in 12 N hydrochloric acid (top) compared to neat DMF (bottom) and (b) 2.0 vol% DMSO in 12 N hydrochloric acid (top) compared to neat DMSO (bottom).

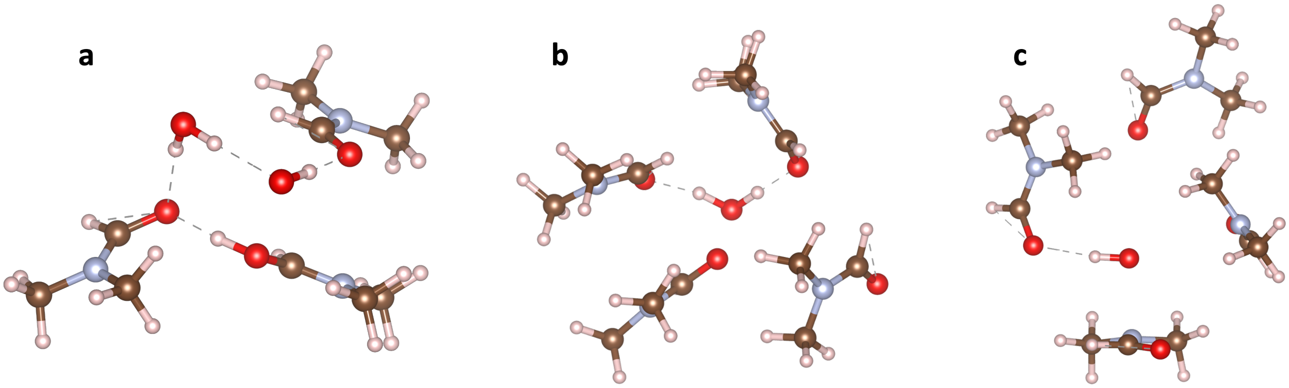

Figure S28. Optimized solvent clusters of the $^{\cdot}$OH radical in (a) two DMF molecules with one H^+-DMF molecule and one water molecule, (b) two DMF molecules with one H^+-DMF, (c) four DMF molecules. The $^{\cdot}$OH radical binding energies are calculated to be 22.4, 32.8, and 32.8 kcal mol^{-1} for (a), (b), and (c), respectively.

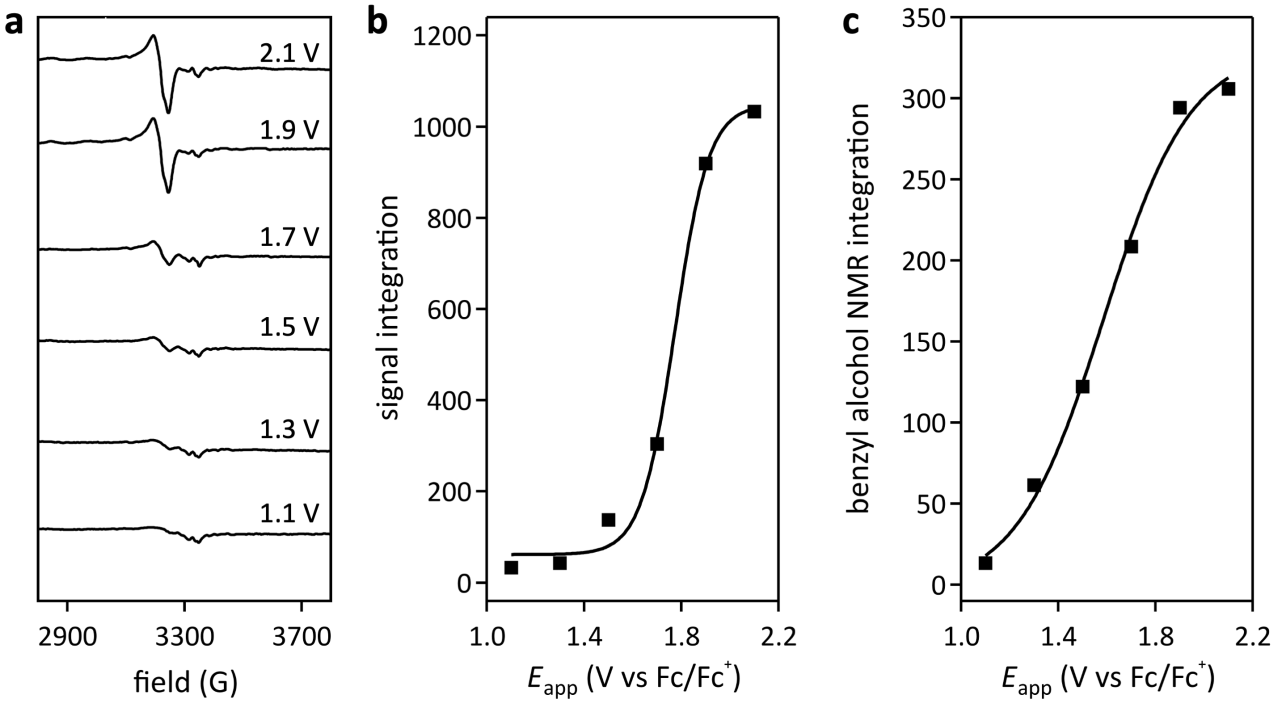

Figure S29. (a) EPR data taken at 10 K of freeze-trapped (non-spin-trapped) electrolyte samples after electrooxidation for 3 h at different applied potentials, E_{app}, of 20 vol% toluene in DMF electrolyte with 0.1 M $LiClO_4$ and 7.0 vol% water. Integration of (b) these EPR signals and (c) NMR data assigned to benzyl alcohol, shown in **Figure S10**.

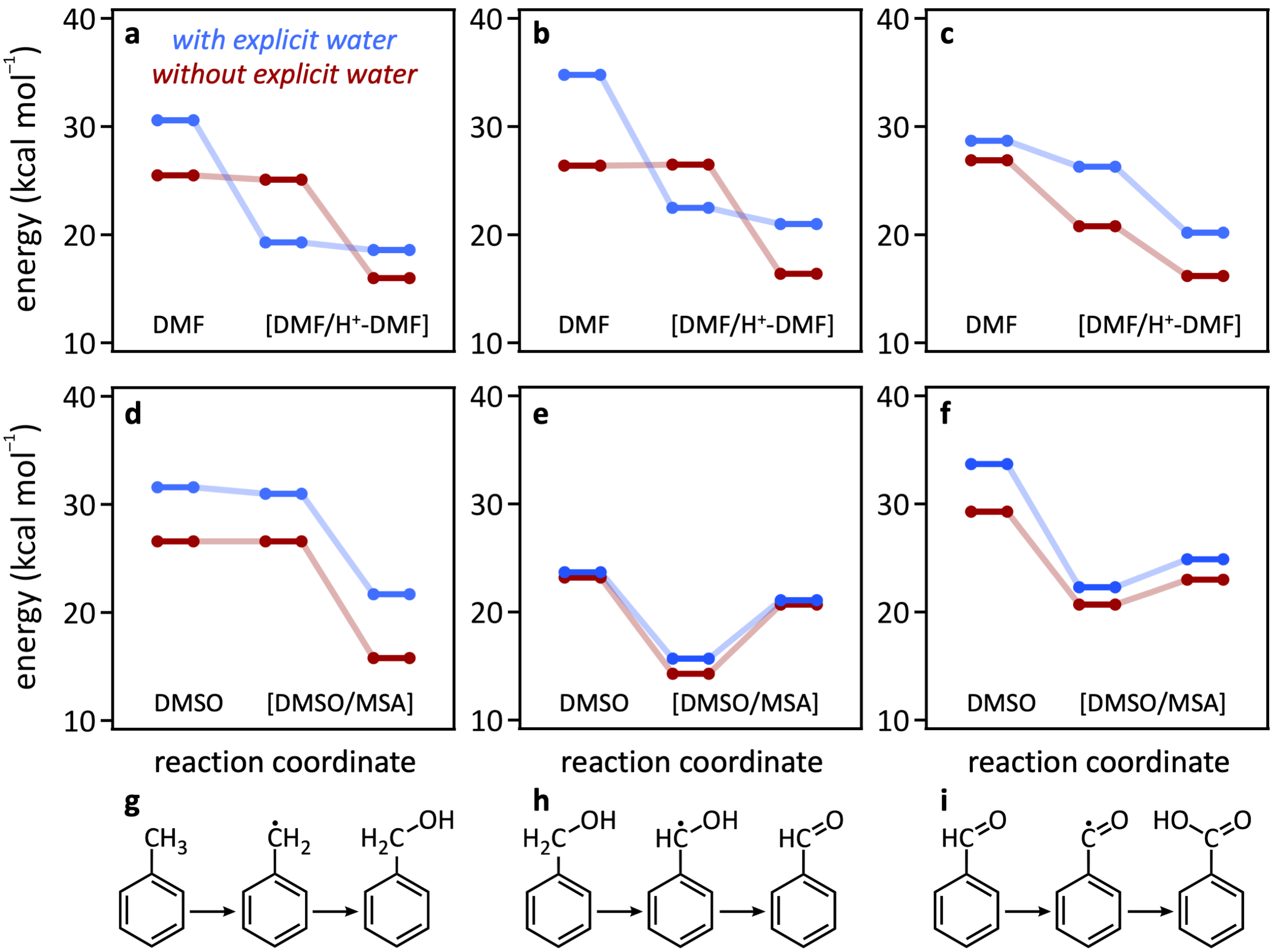

Figure S30. Calculated energies in each solvent, (a–c) DMF, (d–f) DMSO. Each reaction intermediate is modeled with three explicit solvent molecules (red) or three explicit solvent molecules and one explicit water molecule (blue) together with the conductor-like polarizable continuum model (CPCM). (g–i) Molecular representation of the respective one-step oxygenations.

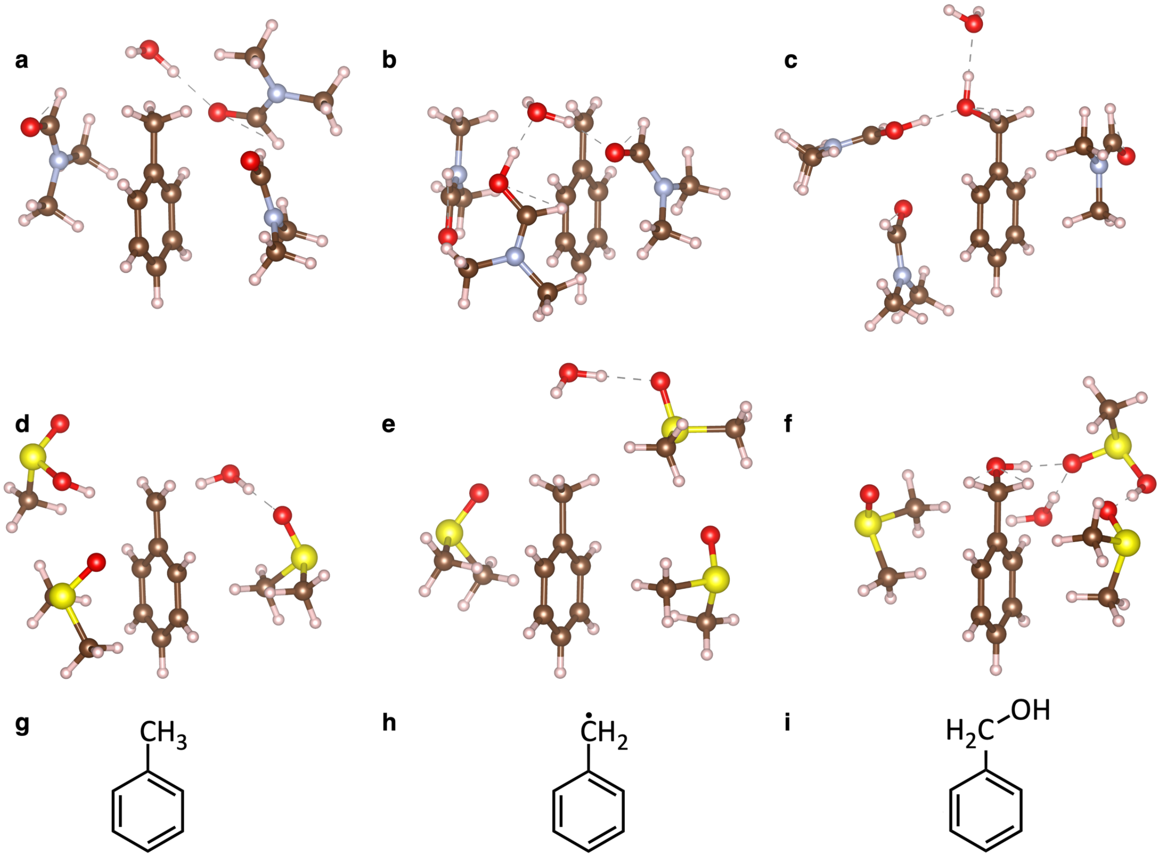

Figure S31. Full optimized geometries of (a) toluene in three DMF molecules and one water molecule, (b) followed by benzyl alcohol radical and (c) benzyl alcohol in two DMF molecules, one H^{+}-DMF molecule, and one water molecule. Full optimized geometries of (d) toluene in three DMSO molecules and one water molecule, (e) followed by benzyl alcohol radical and (f) benzyl alcohol in two DMSO molecules, one MSA molecule, and one water molecule. Cluster forming energies of all intermediates are reported in **Table S5**. (g-i) Corresponding molecular structures of the substrate.

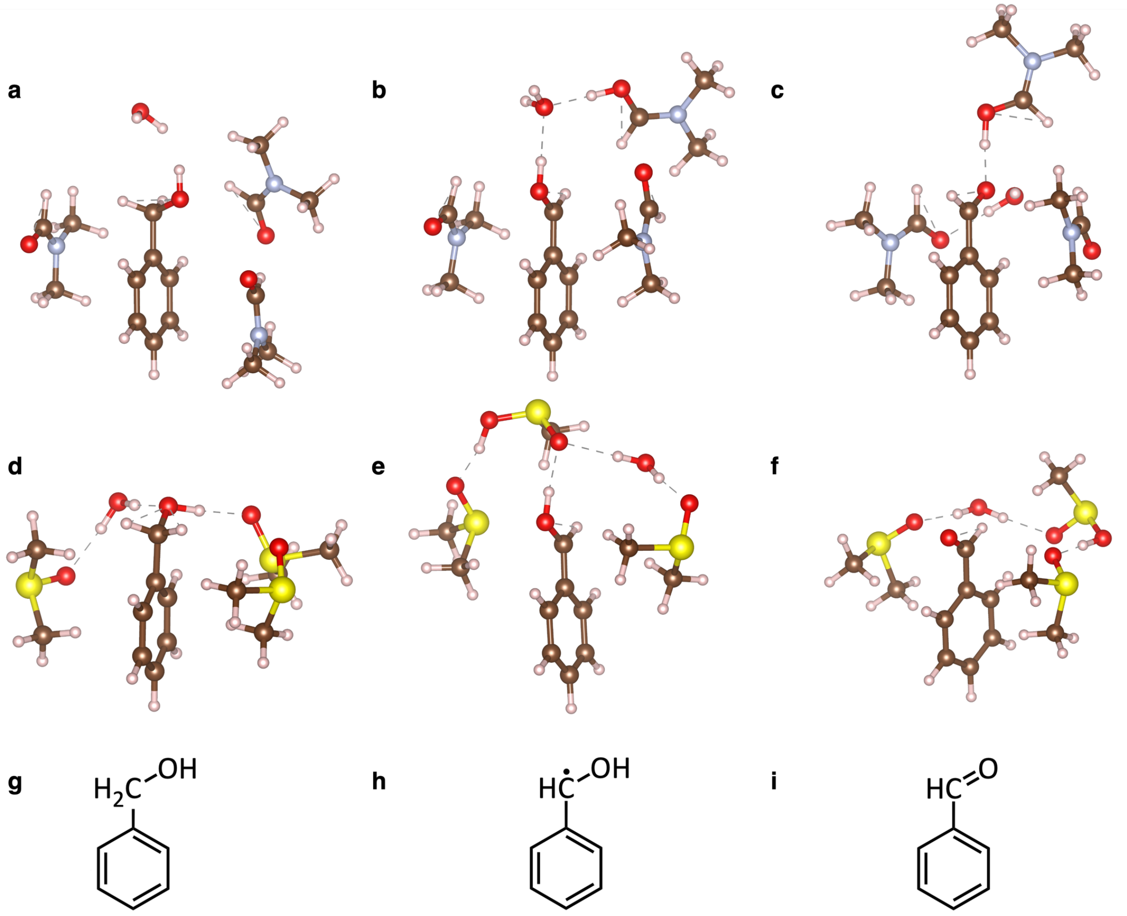

Figure S32. Full optimized geometries of (a) benzyl alcohol in three DMF molecules and one water molecule, (b) followed by benzaldehyde radical and (c) benzaldehyde in two DMF molecules, one H^+-DMF molecule, and one water molecule. Full optimized geometries of (d) benzyl alcohol in three DMSO molecules and one water molecule, (e) followed by benzaldehyde radical and (f) benzaldehyde in two DMSO molecules, one MSA molecule, and one water molecule. Cluster forming energies of all intermediates are reported in **Table S5**. (g-i) Corresponding molecular structures of the substrate.

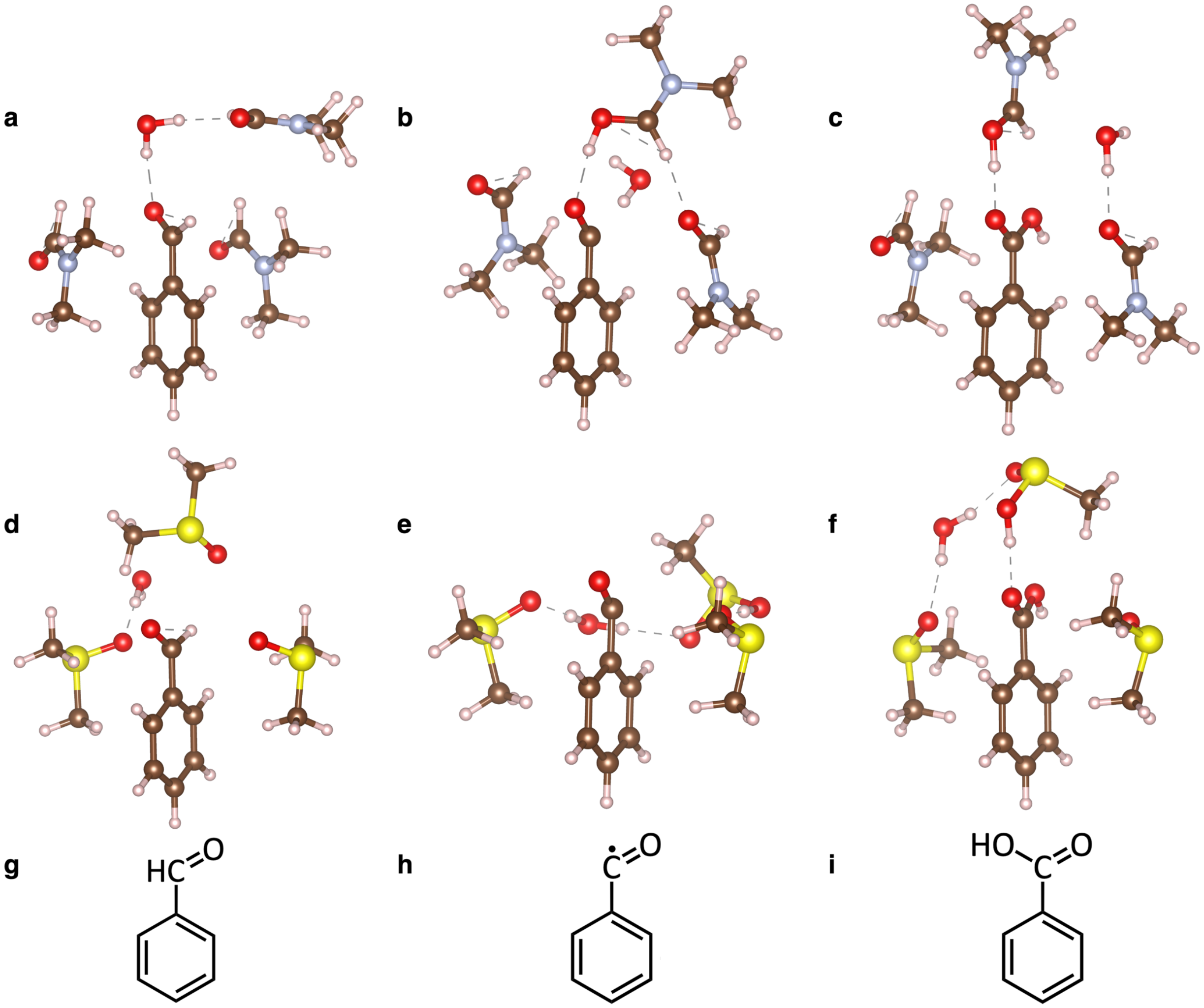

Figure S33. Full optimized geometries of (a) benzaldehyde in three DMF molecules and one water molecule, (b) followed by benzoic acid radical and (c) benzoic acid in two DMF molecules, one H^+-DMF molecule, and one water molecule. Full optimized geometries of (d) benzaldehyde in three DMSO molecules and one water molecule, (e) followed by benzoic acid radical and (f) benzoic acid in two DMSO molecules, one MSA molecule, and one water molecule. Cluster forming energies of all intermediates are reported in **Table S5**. (g-i) Corresponding molecular structures of the substrate.

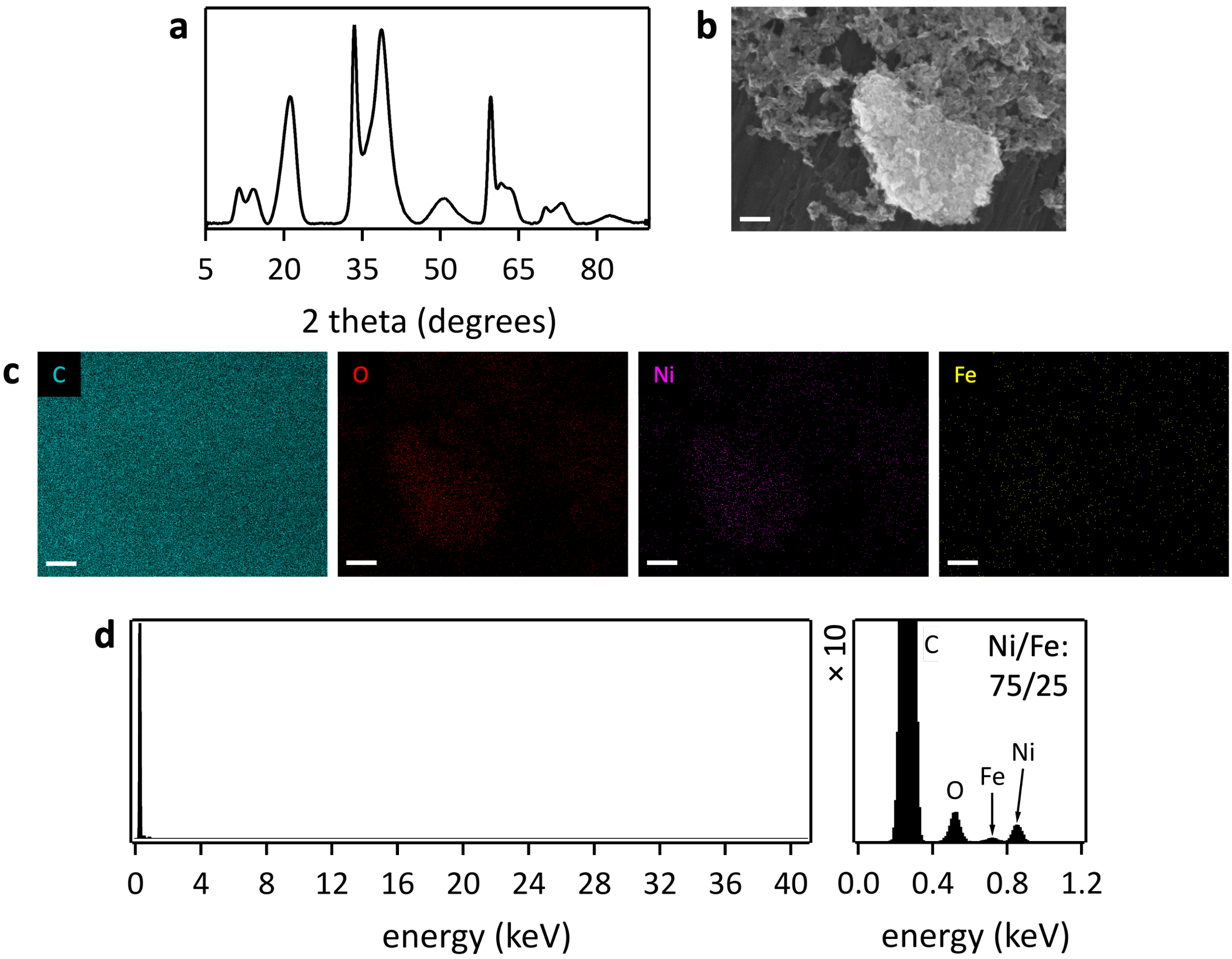

Figure S34. (a) XRD data of $[Ni_{0.75}Fe_{0.25}]$-$(OH)_2$ nanosheets prepared by precipitation. (b) SEM images with a scalebar of 100 nm of laser-synthesized $[Ni_{0.75}Fe_{0.25}]$-$(OH)_2$ nanosheets on hydrophilic carbon fiber paper (dark). (c) Corresponding SEM-EDX maps with scalebars of 100 nm. (d) EDX spectra of $[Ni_{0.75}Fe_{0.25}]$-$(OH)_2$ on hydrophilic carbon fiber paper, showing the full energy axis (left) and expanded axes (right).

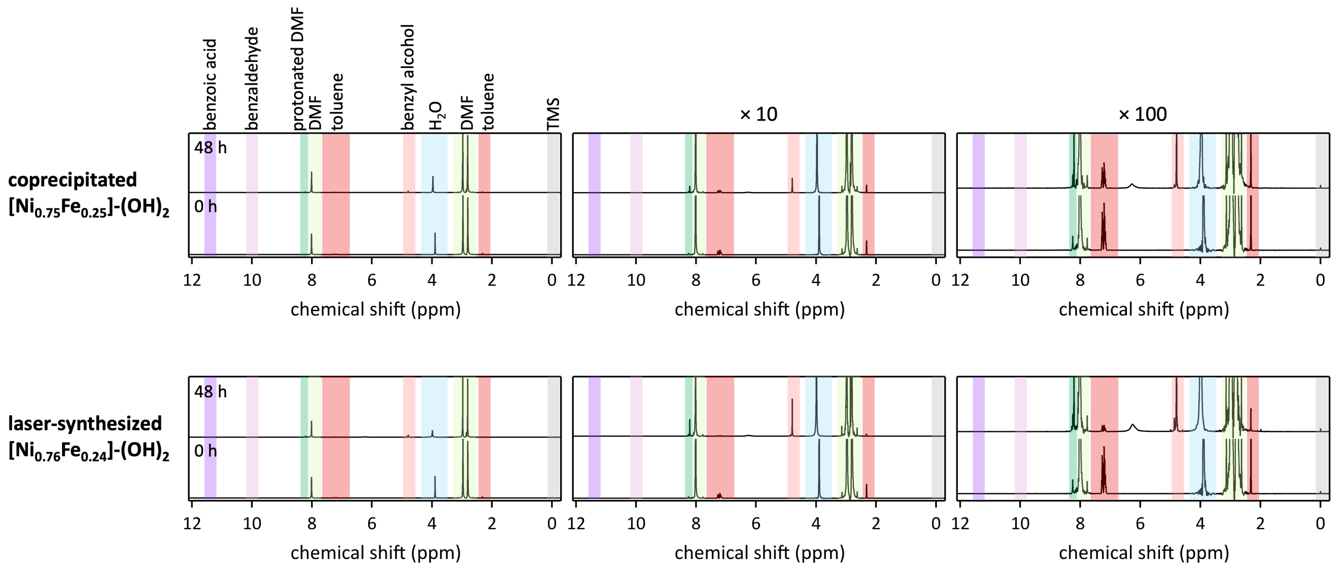

Figure S35. NMR data of electrolyte aliquots collected pre and post 48 h toluene electrooxidation at 2.1 V. Toluene electrooxidation was conducted in $LiClO_4$-supported DMF electrolyte with 7.0 vol% water, electrocatalyzed by coprecipitated [$Ni_{0.75}Fe_{0.25}$]-$(OH)_2$ nanosheets on hydrophilic carbon fiber paper (top) and laser-synthesized [$Ni_{0.76}Fe_{0.24}$]-$(OH)_2$ nanosheets on hydrophilic carbon fiber paper (bottom).

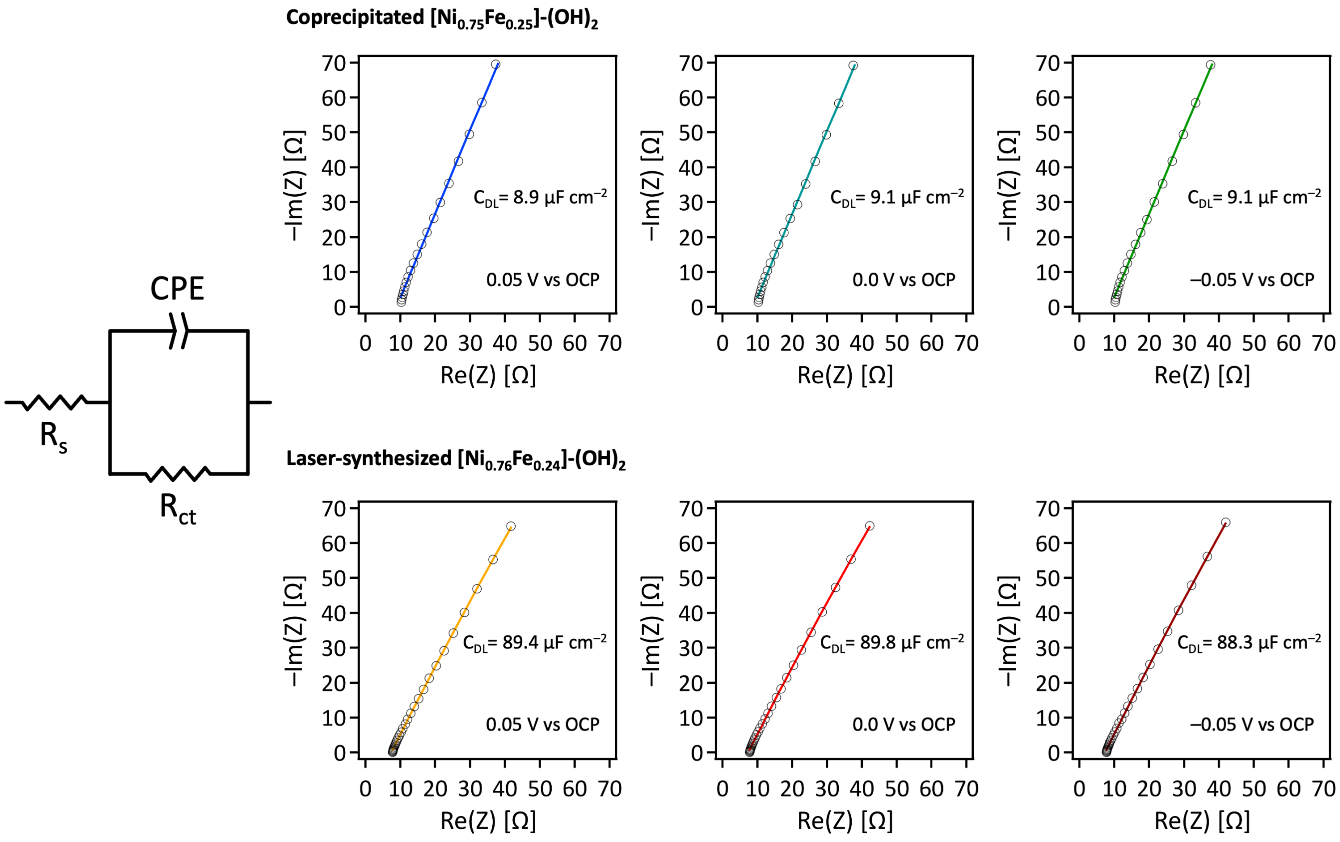

Figure S36. Electrical impedance spectroscopy (EIS) data shown as Nyquist plots for coprecipitated [$Ni_{0.75}Fe_{0.25}$]-$(OH)_2$ nanosheets on hydrophilic carbon fiber paper (top) and laser-synthesized [$Ni_{0.76}Fe_{0.24}$]-$(OH)_2$ nanosheets on hydrophilic carbon fiber paper (bottom) in 1.0 M aqueous NaOH, collected at different applied potentials vs open circuit potential (OCP). Data, circles; solid lines, fits of the data using the Randles circuit (shown left), with R_s, solvent resistance; R_{ct}, charge transfer resistance; CPE, constant-phase element.

Table S1. Simulation parameters of the 298 K EPR data shown in **Figure S22**, using DMF electrolyte and MNP as spin trap.

simulation parameters	
g value	2.0065
Nuclei	N
A (MHz)	44.31 (15.7 G)
Lw gaussian (mT)	0.067
Lw lorentzian (mT)	0.026

Table S2. Simulation parameters of the 298 K EPR data shown in **Figures S22**, using DMSO electrolyte and PBN as spin trap.

simulation parameters	
g value	2.0066
Nuclei	N, H
A – N (MHz)	38.91 (13.8 G)
A – H (MHz)	6.14 (2.2 G)
Lw gaussian (mT)	0.011
Lw lorentzian (mT)	0.067

Table S3. Simulation parameters of the simulated 10 K EPR data shown in **Figure 2b**, using DMF electrolyte and MNP as spin trap.

simulation parameters	
g1 value	2.0080
g2 value	2.0060
g3 value	2.0014
Nuclei	N
[A1 A2 A3] (MHz)	[0 0 91.4]
Lw gaussian (mT)	0.014
Lw lorentzian (mT)	0.011
gStrain	0.010

Table S4. Diffusion constants for toluene determined by DOSY ^{1}H-NMR.

solution	diffusion constant (m^2 s^{-1})
2 vol% toluene in DMSO	$(6.61 \pm 0.02) \times 10^{-10}$
2 vol% toluene in 0.1M $LiClO_4$ in DMSO with 7 vol% H_2O	$(6.00 \pm 0.04) \times 10^{-10}$
2 vol% toluene in DMF	$(7.87 \pm 0.30) \times 10^{-9}$
2 vol% toluene in 0.1M $LiClO_4$ in DMF with 7 vol% H_2O	$(1.11 \pm 0.02) \times 10^{-9}$

Table S5. Cluster forming energies of each intermediate from **Figures S31–S33** in kcal mol^{-1}.*

molecule	wet DMF	wet [H$^+$-DMF/DMF]	wet DMSO	wet [MSA/DMSO]
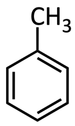	30.6	17.8	31.6	26.5
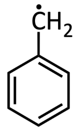	27.9	19.3	30.0	31.0
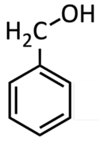	34.8	18.6	23.7	21.7
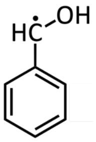	26.0	22.5	22.4	15.7
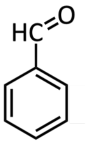	28.7	21.0	15.7	21.1
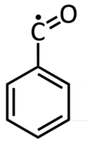	30.4	26.3	34.7	22.3
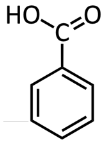	31.2	20.2	31.0	24.9

* Each reaction intermediate is modeled with three explicit solvent molecules together with the conductor-like polarizable continuum model (CPCM). Geometry optimizations are performed using B3LYP-D3BJ/def2-TZVP level of theory.

Table S6. Energy corresponding to the thermodynamic favorability of protonated versus neutral solvation environments for each species.

species	$\Delta G_{\text{protonation}}$ (kcal mol^{-1})	species	$\Delta G_{\text{protonation}}$ (kcal mol^{-1})
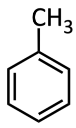	–13.8	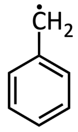	–9.6
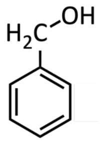	–17.3	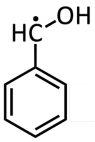	-4.6
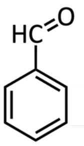	–8.7	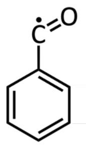	–5.0
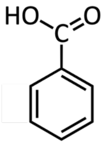	–12.0		

Table S7. C–H bond cleavage energies for toluene, benzyl alcohol, and benzaldehyde to form the respective radical.

reaction	$\Delta G_{\text{C–H bond cleavage}}$ (kcal mol^{-1})
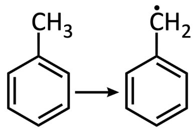	81.7
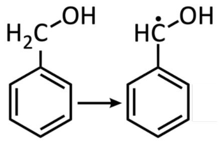	67.4
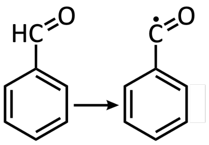	89.3

Cartesian Coordinates of Cluster Models Used in the DFT Calculations

Structures in 3 DMF and 1 H_2O

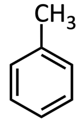

C	3.025	28.113	22.960
C	1.681	28.149	22.584
C	1.002	26.983	22.250
C	1.660	25.758	22.273
C	2.998	25.708	22.650
C	3.670	26.877	22.994
C	3.766	29.381	23.277
H	1.165	29.100	22.557
H	-0.042	27.033	21.966
H	1.133	24.849	22.008
H	3.520	24.760	22.680
H	4.711	26.829	23.292
H	4.606	29.193	23.947
H	3.111	30.118	23.741
H	1.073	28.096	17.869
H	3.095	26.494	17.456
H	0.754	27.226	19.384
C	1.334	28.030	18.928
H	2.743	25.684	18.995
C	3.261	26.528	18.535
N	2.753	27.773	19.083
C	3.560	28.656	19.689
O	4.777	28.544	19.830
H	3.014	29.531	20.067
H	1.076	28.967	19.417
H	4.325	26.457	18.741
H	0.838	30.164	25.945
H	2.487	26.358	26.306
H	-0.234	28.913	26.611
C	0.564	29.113	25.891
H	0.788	26.520	26.808
C	1.543	26.848	26.090
N	1.722	28.286	26.180
C	2.901	28.829	26.514
O	3.930	28.210	26.786
H	2.870	29.928	26.522
H	0.192	28.898	24.888
H	1.215	26.580	25.085
O	0.557	31.512	22.345
C	0.726	31.591	21.124
N	1.810	32.061	20.502
H	-0.052	31.250	20.428
C	1.865	32.145	19.051
H	1.955	33.188	18.738
H	0.960	31.720	18.623
H	2.729	31.593	18.678
C	2.966	32.562	21.228
H	2.819	32.420	22.293
H	3.106	33.625	21.018
H	3.859	32.021	20.909
H	4.166	29.829	22.364
O	1.503	32.548	24.748
H	2.254	32.023	25.050
H	1.247	32.157	23.888

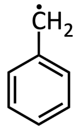

C	1.186	27.669	22.389
C	0.585	26.604	23.116
C	1.354	25.686	23.803
C	2.749	25.787	23.799
C	3.363	26.829	23.097
C	2.604	27.751	22.406
C	0.411	28.598	21.683
H	-0.496	26.522	23.120
H	0.874	24.883	24.347
H	3.349	25.066	24.338
H	4.442	26.914	23.096
H	3.085	28.554	21.863

H	-0.668	28.523	21.675
H	2.118	24.180	19.399
H	4.508	25.077	18.963
H	2.141	24.928	21.010
C	1.934	25.102	19.953
H	4.501	25.795	20.587
C	4.222	25.956	19.544
N	2.787	26.165	19.449
C	2.272	27.296	18.967
O	2.923	28.258	18.540
H	1.175	27.312	18.980
H	0.890	25.386	19.840
H	4.738	26.831	19.161
H	-0.041	30.942	24.151
H	3.254	28.988	25.780
H	-0.176	30.461	25.856
C	0.215	30.172	24.877
H	1.862	29.223	26.864
C	2.170	29.002	25.840
N	1.656	30.017	24.937
C	2.470	30.761	24.177
O	3.699	30.705	24.160
H	1.918	31.465	23.537
H	-0.253	29.233	24.575
H	1.780	28.023	25.557
O	0.736	31.810	20.100
C	1.450	32.550	20.786
N	2.753	32.387	21.027
H	1.031	33.438	21.280
C	3.502	33.359	21.810
H	2.830	34.138	22.167
H	4.280	33.817	21.195
H	3.967	32.865	22.663
C	3.504	31.265	20.488
H	2.821	30.516	20.101
H	4.108	30.827	21.283
H	4.165	31.598	19.684
H	0.868	29.416	21.143
O	1.173	30.283	17.763
H	1.819	29.588	18.007
H	1.049	30.801	18.580

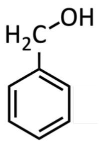

C	2.403	28.674	23.182
C	1.382	28.005	22.513
C	1.528	26.672	22.144
C	2.707	25.997	22.432
C	3.735	26.658	23.101
C	3.580	27.985	23.479
C	2.242	30.113	23.574
H	0.468	28.534	22.271
H	0.725	26.166	21.623
H	2.828	24.962	22.139
H	4.653	26.134	23.335
H	4.375	28.492	24.012
O	3.137	30.915	22.788
H	3.033	31.834	23.064
H	2.470	30.248	24.635
H	1.208	30.425	23.407
H	2.058	26.310	17.659
H	4.621	26.053	17.963
H	1.870	25.707	19.317
C	2.040	26.600	18.713
H	4.404	25.542	19.649
C	4.491	26.408	18.988
N	3.296	27.225	19.085
C	3.339	28.490	19.529
O	4.357	29.103	19.855
H	2.344	28.954	19.574
H	1.222	27.298	18.876
H	5.353	27.000	19.279
H	0.221	29.805	26.408
H	2.386	26.250	26.329
H	-0.704	28.328	26.752
C	0.111	28.751	26.159
H	0.646	26.092	26.665
C	1.400	26.638	26.093
N	1.353	28.050	26.430
C	2.405	28.680	26.972
O	3.493	28.173	27.240
H	2.202	29.745	27.164

H	-0.146	28.663	25.102
H	1.202	26.505	25.029
O	0.076	30.049	19.836
C	0.431	31.071	20.423
N	1.300	31.982	19.960
H	0.042	31.328	21.419
C	1.657	33.152	20.741
H	2.733	33.168	20.926
H	1.135	33.130	21.697
H	1.381	34.063	20.206
C	1.938	31.836	18.664
H	1.524	30.969	18.157
H	3.015	31.703	18.787
H	1.762	32.730	18.062
O	0.503	33.023	24.911
H	1.462	32.916	24.932
H	0.235	32.635	24.069

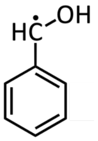

C	2.052	27.574	22.631
C	1.083	26.538	22.563
C	1.439	25.219	22.758
C	2.767	24.872	23.031
C	3.733	25.879	23.103
C	3.394	27.205	22.909
C	1.662	28.906	22.436
H	0.052	26.799	22.354
H	0.681	24.448	22.700
H	3.042	23.837	23.183
H	4.764	25.620	23.314
H	4.150	27.975	22.974
O	2.554	29.909	22.543
H	2.091	30.775	22.433
H	0.634	29.173	22.223
H	0.968	26.409	18.033
H	3.329	25.352	18.372
H	0.736	26.217	19.783
C	1.134	26.874	19.007
H	3.094	25.292	20.132
C	3.402	25.926	19.299
N	2.551	27.099	19.226
C	3.042	28.336	19.384
O	4.223	28.629	19.570
H	2.259	29.105	19.327
H	0.605	27.824	19.043
H	4.430	26.242	19.449
H	0.256	29.839	25.377
H	3.187	27.076	26.423
H	-0.219	28.607	26.565
C	0.373	28.797	25.667
H	1.617	26.896	27.243
C	2.111	27.154	26.302
N	1.774	28.512	25.919
C	2.713	29.457	25.774
O	3.920	29.311	25.966
H	2.294	30.421	25.454
H	0.000	28.161	24.862
H	1.782	26.458	25.529
O	1.270	32.246	22.421
C	1.544	33.271	21.766
N	1.992	33.285	20.520
H	1.414	34.263	22.211
C	2.363	34.532	19.864
H	3.422	34.511	19.601
H	2.179	35.368	20.535
H	1.775	34.659	18.954
C	2.184	32.053	19.766
H	1.532	31.278	20.159
H	3.222	31.718	19.828
H	1.934	32.240	18.722
O	-0.001	32.594	24.886
H	0.473	32.463	24.039
H	0.635	32.347	25.567

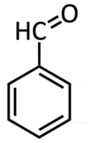

C	2.143	27.328	22.276
C	0.808	26.956	22.457
C	0.478	25.982	23.389
C	1.483	25.380	24.140
C	2.817	25.751	23.964
C	3.149	26.721	23.035
C	2.458	28.340	21.268
H	0.038	27.431	21.861
H	-0.555	25.691	23.529
H	1.229	24.619	24.867
H	3.591	25.276	24.553
H	4.178	27.016	22.882
O	3.586	28.718	20.992
H	1.589	28.759	20.734
H	1.981	25.296	17.985
H	2.620	23.445	19.768
H	1.357	26.293	19.311
C	2.258	26.017	18.758
H	1.939	24.520	21.006
C	2.837	24.286	20.431
N	3.243	25.447	19.658
C	4.489	25.939	19.732
O	5.397	25.496	20.433
H	4.634	26.815	19.084
H	2.666	26.908	18.283
H	3.639	24.014	21.111
H	1.282	31.124	22.783
H	2.886	28.790	25.826
H	0.429	31.221	24.336
C	0.981	30.561	23.662
H	1.443	29.732	26.273
C	1.932	29.169	25.473
N	2.156	30.024	24.322
C	3.394	30.335	23.906
O	4.443	29.952	24.421
H	3.390	30.985	23.021
H	0.320	29.749	23.353
H	1.290	28.333	25.191
O	1.573	31.942	19.486
C	2.183	32.523	20.391
N	1.636	33.334	21.301
H	3.266	32.401	20.520
C	2.445	33.978	22.323
H	3.486	33.684	22.204
H	2.102	33.678	23.316
H	2.365	35.063	22.237
C	0.210	33.620	21.310
H	-0.273	33.055	20.520
H	0.044	34.688	21.153
H	-0.215	33.338	22.276
O	3.410	30.091	18.479
H	3.584	29.614	19.310
H	2.725	30.744	18.729

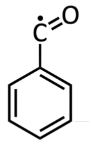

C	2.862	28.267	22.583
C	1.594	28.849	22.517
C	0.465	28.065	22.699
C	0.604	26.703	22.945
C	1.869	26.118	23.013
C	3.000	26.894	22.832
C	4.039	29.108	22.385
H	1.511	29.910	22.327
H	-0.519	28.511	22.648
H	-0.277	26.089	23.084
H	1.965	25.058	23.206
H	3.988	26.459	22.886
O	5.197	28.827	22.394
H	-0.225	27.221	18.264
H	0.829	24.808	18.590
H	-0.398	27.382	20.023
C	0.291	27.496	19.186
H	0.481	25.055	20.314
C	1.215	25.221	19.526
N	1.455	26.648	19.383
C	2.690	27.150	19.360

O	3.734	26.494	19.458
H	2.712	28.241	19.245
H	0.603	28.536	19.124
H	2.145	24.726	19.786
H	4.569	30.766	25.332
H	2.733	27.122	26.151
H	3.277	30.814	26.550
C	3.605	30.348	25.618
H	2.168	28.497	27.129
C	2.519	28.186	26.141
N	3.725	28.911	25.784
C	4.906	28.288	25.655
O	5.104	27.083	25.800
H	5.719	28.979	25.388
H	2.876	30.580	24.839
H	1.734	28.392	25.412
O	3.051	33.052	23.062
C	3.225	32.432	22.014
N	2.279	32.152	21.102
H	4.211	32.039	21.732
C	2.597	31.439	19.878
H	3.632	31.103	19.906
H	2.453	32.086	19.009
H	1.946	30.568	19.775
C	0.905	32.592	21.272
H	0.811	33.102	22.226
H	0.232	31.732	21.250
H	0.624	33.273	20.465
O	5.962	28.130	19.484
H	5.185	27.528	19.479
H	5.989	28.478	20.383

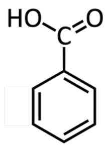

C	1.582	27.828	21.878
C	0.450	27.156	22.346
C	0.585	26.102	23.238
C	1.847	25.714	23.673
C	2.977	26.386	23.217
C	2.846	27.438	22.326
C	1.510	28.944	20.902
H	-0.548	27.449	22.043
H	-0.297	25.588	23.597
H	1.950	24.890	24.367
H	3.961	26.086	23.554
H	3.716	27.964	21.963
O	2.458	29.660	20.641
O	0.347	29.184	20.282
H	2.395	22.744	20.159
H	4.437	24.179	19.274
H	2.455	23.640	21.691
C	2.072	23.655	20.668
H	4.446	24.947	20.874
C	4.006	24.983	19.876
N	2.566	24.828	19.970
C	1.728	25.709	19.405
O	2.056	26.710	18.770
H	0.672	25.453	19.574
H	0.985	23.679	20.701
H	4.231	25.941	19.415
H	0.923	31.349	23.036
H	2.689	28.784	25.795
H	0.149	31.287	24.632
C	0.669	30.700	23.871
H	1.233	29.639	26.357
C	1.713	29.159	25.501
N	1.878	30.114	24.420
C	3.089	30.441	23.945
O	4.165	30.009	24.356
H	3.034	31.156	23.114
H	-0.003	29.915	23.519
H	1.089	28.329	25.168
O	5.118	32.711	21.655
C	4.049	32.726	21.045
N	2.919	33.325	21.453
H	3.930	32.220	20.078
C	1.718	33.306	20.637
H	1.893	32.719	19.738
H	1.436	34.324	20.354
H	0.891	32.859	21.193
C	2.858	34.065	22.702

H	3.794	33.937	23.237
H	2.034	33.691	23.313
H	2.692	35.127	22.505
H	-0.319	28.507	20.471
O	2.287	30.745	18.003
H	2.359	30.427	18.923
H	3.121	31.199	17.833

Structures in 2 DMF, 1 H^+-DMF, and 1 H_2O

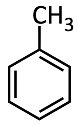

C	1.041	28.111	22.142
C	0.161	27.218	22.759
C	0.636	26.142	23.497
C	2.006	25.938	23.636
C	2.892	26.817	23.023
C	2.411	27.891	22.282
C	0.518	29.294	21.374
H	-0.908	27.371	22.660
H	-0.063	25.463	23.970
H	2.378	25.104	24.217
H	3.960	26.670	23.123
H	3.109	28.572	21.814
H	1.296	29.738	20.754
H	0.154	30.067	22.056
H	1.561	26.803	17.637
H	3.704	25.629	18.478
H	1.025	26.986	19.319
C	1.631	27.432	18.527
H	3.156	25.834	20.157
C	3.685	26.313	19.330
N	3.012	27.545	18.960
C	3.623	28.734	19.050
O	4.777	28.926	19.433
H	2.973	29.567	18.742
H	1.236	28.420	18.296
H	4.701	26.540	19.637
H	-0.169	28.838	25.693
H	3.929	28.348	26.298
H	0.342	28.553	27.374
C	0.537	28.308	26.328
H	2.917	28.290	27.761
C	2.973	28.033	26.702
N	1.896	28.694	25.977
C	2.129	29.604	25.052
O	3.275	30.009	24.738
H	1.239	30.008	24.565
H	0.414	27.235	26.182
H	2.864	26.953	26.592
O	3.648	31.174	22.617
C	2.674	31.875	22.171
H	3.431	30.705	23.567
N	2.699	32.418	20.990
H	1.782	32.022	22.780
C	1.583	33.239	20.526
H	1.946	34.241	20.297
H	0.821	33.294	21.299
H	1.163	32.794	19.625
C	3.831	32.258	20.078
H	4.414	31.387	20.357
H	4.453	33.154	20.107
H	3.439	32.127	19.071
H	-0.314	29.011	20.728
O	-0.032	31.902	23.935
H	-0.056	32.499	24.693
H	-0.784	32.163	23.388

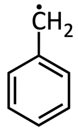

C	0.902	26.760	22.716
C	1.538	25.533	23.049
C	2.914	25.440	23.113
C	3.713	26.557	22.849
C	3.111	27.776	22.524
C	1.737	27.882	22.461
C	-0.493	26.859	22.636
H	0.923	24.664	23.250

H	3.378	24.495	23.367
H	4.791	26.479	22.897
H	3.728	28.644	22.331
H	1.273	28.827	22.218
H	-1.123	26.002	22.830
H	1.589	25.437	18.328
H	3.122	27.383	17.896
H	2.070	25.562	20.035
C	1.361	25.913	19.283
H	3.466	27.748	19.601
C	2.756	27.898	18.784
N	1.458	27.358	19.153
C	0.441	28.148	19.477
O	0.472	29.391	19.470
H	-0.464	27.604	19.769
H	0.354	25.639	19.592
H	2.661	28.960	18.579
H	-0.667	30.374	24.710
H	2.892	28.594	25.927
H	-0.712	29.529	26.272
C	-0.299	29.510	25.260
H	1.488	28.354	26.994
C	1.819	28.431	25.956
N	1.152	29.544	25.305
C	1.838	30.558	24.761
O	3.064	30.677	24.745
H	1.184	31.313	24.304
H	-0.637	28.601	24.759
H	1.581	27.500	25.439
O	1.481	32.037	21.815
C	2.427	31.442	21.179
H	0.555	31.588	21.619
N	3.668	31.769	21.353
H	2.187	30.643	20.480
C	4.730	31.115	20.588
H	4.297	30.381	19.915
H	5.415	30.625	21.279
H	5.270	31.868	20.014
C	4.052	32.821	22.299
H	3.461	32.725	23.206
H	3.895	33.801	21.847
H	5.104	32.693	22.534
H	-0.968	27.794	22.371
O	-0.692	30.899	21.288
H	-0.419	30.249	20.582
H	-1.001	30.375	22.040

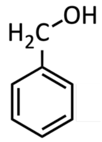

C	1.926	28.795	23.655
C	0.985	28.155	22.850
C	1.326	27.009	22.140
C	2.615	26.495	22.227
C	3.561	27.130	23.025
C	3.217	28.272	23.737
C	1.563	30.037	24.412
H	-0.020	28.554	22.785
H	0.588	26.520	21.517
H	2.883	25.605	21.673
H	4.565	26.730	23.098
H	3.949	28.756	24.371
O	2.046	31.200	23.694
H	1.893	32.008	24.248
H	2.028	30.041	25.398
H	0.480	30.110	24.534
H	2.661	26.618	17.603
H	5.125	27.062	18.287
H	2.370	25.901	19.200
C	2.401	26.839	18.641
H	4.801	26.353	19.881
C	4.750	27.271	19.291
N	3.378	27.740	19.222
C	3.016	28.926	19.725
O	3.767	29.752	20.246
H	1.937	29.114	19.625
H	1.415	27.299	18.670
H	5.366	28.034	19.758
H	-0.202	28.521	27.153
H	2.654	25.725	25.992
H	-0.758	26.838	27.044
C	-0.104	27.590	26.597

H	1.037	25.079	26.359
C	1.587	25.915	25.921
N	1.276	27.144	26.629
C	2.218	27.832	27.291
O	3.407	27.527	27.379
H	1.819	28.738	27.770
H	-0.422	27.758	25.565
H	1.306	26.013	24.872
O	1.161	31.466	21.386
C	2.057	31.937	20.597
H	1.524	31.358	22.370
N	1.824	32.156	19.340
H	3.040	32.183	20.981
C	2.880	32.663	18.467
H	3.086	31.924	17.693
H	3.780	32.844	19.047
H	2.545	33.590	18.002
C	0.537	31.867	18.710
H	-0.162	31.492	19.450
H	0.687	31.122	17.928
H	0.151	32.783	18.263
O	1.572	33.353	25.233
H	2.369	33.833	25.496
H	1.015	34.006	24.787

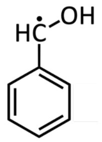

C	1.889	27.629	22.948
C	0.532	27.421	22.586
C	0.031	26.146	22.417
C	0.853	25.027	22.599
C	2.191	25.214	22.953
C	2.710	26.484	23.125
C	2.370	28.933	23.121
H	-0.111	28.281	22.443
H	-1.008	26.012	22.143
H	0.456	24.029	22.466
H	2.834	24.354	23.096
H	3.746	26.616	23.405
O	3.659	29.153	23.453
H	3.806	30.113	23.561
H	1.729	29.797	23.003
H	2.748	26.510	18.549
H	5.171	27.295	19.173
H	2.401	26.247	20.272
C	2.424	26.991	19.474
H	4.759	26.956	20.866
C	4.716	27.708	20.077
N	3.333	28.069	19.823
C	2.904	29.331	19.937
O	3.599	30.311	20.213
H	1.824	29.426	19.753
H	1.421	27.390	19.336
H	5.264	28.593	20.388
H	0.817	29.905	26.033
H	1.809	25.890	26.489
H	-0.393	28.891	26.847
C	0.361	28.917	26.056
H	0.221	26.378	27.128
C	0.947	26.529	26.325
N	1.381	27.915	26.300
C	2.667	28.255	26.462
O	3.599	27.479	26.673
H	2.827	29.340	26.389
H	-0.130	28.732	25.099
H	0.480	26.269	25.373
O	3.268	33.118	21.567
C	2.242	32.564	21.011
H	3.508	32.664	22.433
N	1.769	32.987	19.885
H	1.735	31.748	21.510
C	0.609	32.337	19.275
H	-0.170	33.081	19.114
H	0.242	31.551	19.929
H	0.907	31.911	18.317
C	2.385	34.073	19.122
H	3.225	34.485	19.670
H	1.636	34.845	18.949
H	2.723	33.677	18.164
O	3.926	31.921	23.800
H	4.840	32.138	24.039

H	3.383	32.195	24.555

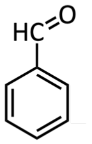

C	1.867	27.156	22.487
C	0.880	26.188	22.707
C	1.197	25.016	23.376
C	2.500	24.811	23.823
C	3.489	25.771	23.601
C	3.179	26.941	22.935
C	1.491	28.376	21.807
H	-0.129	26.366	22.354
H	0.438	24.266	23.550
H	2.750	23.897	24.346
H	4.498	25.598	23.950
H	3.935	27.691	22.758
O	2.260	29.315	21.589
H	0.446	28.447	21.476
H	1.631	25.750	17.862
H	3.437	24.310	19.089
H	0.903	26.174	19.424
C	1.687	26.435	18.710
H	2.605	24.716	20.603
C	3.364	25.046	19.892
N	2.989	26.341	19.348
C	3.805	27.395	19.421
O	4.929	27.400	19.935
H	3.374	28.304	18.981
H	1.523	27.452	18.358
H	4.320	25.134	20.397
H	1.022	30.942	23.973
H	2.914	27.954	26.171
H	0.266	30.519	25.525
C	0.802	30.124	24.658
H	1.441	28.642	26.896
C	1.923	28.343	25.962
N	2.037	29.486	25.072
C	3.231	29.958	24.680
O	4.327	29.503	24.998
H	3.138	30.826	24.011
H	0.153	29.403	24.154
H	1.319	27.564	25.491
O	1.512	31.451	20.465
C	1.962	32.448	21.143
H	1.780	30.557	20.899
N	1.738	33.674	20.799
H	2.552	32.267	22.037
C	2.264	34.783	21.597
H	2.818	34.393	22.448
H	1.431	35.393	21.945
H	2.920	35.388	20.973
C	0.958	34.035	19.614
H	0.615	33.140	19.107
H	1.588	34.624	18.949
H	0.106	34.637	19.929
O	5.204	29.866	21.232
H	5.225	29.022	20.735
H	4.311	29.877	21.601

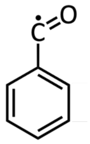

C	2.658	27.276	22.729
C	1.335	27.236	23.179
C	0.763	26.021	23.521
C	1.510	24.851	23.412
C	2.829	24.887	22.959
C	3.409	26.096	22.616
C	3.255	28.555	22.396
H	0.768	28.154	23.252
H	-0.260	25.983	23.868
H	1.063	23.902	23.681
H	3.400	23.972	22.878
H	4.432	26.145	22.268
O	4.343	28.834	21.988
H	-1.862	27.265	19.664
H	0.331	26.122	18.849
H	-1.394	27.004	21.356
C	-1.281	27.668	20.497

H	0.706	25.809	20.555
C	0.792	26.537	19.748
N	0.121	27.769	20.127
C	0.760	28.942	20.136
O	1.947	29.112	19.841
H	0.123	29.779	20.451
H	-1.665	28.653	20.754
H	1.840	26.746	19.558
H	1.934	30.401	25.718
H	4.863	27.450	25.549
H	2.008	29.332	27.136
C	2.131	29.384	26.051
H	3.981	27.530	27.092
C	3.899	27.649	26.008
N	3.480	29.000	25.674
C	4.353	29.904	25.197
O	5.540	29.699	24.955
H	3.887	30.885	25.021
H	1.408	28.713	25.587
H	3.162	26.938	25.633
O	4.600	31.423	21.278
C	3.569	31.663	20.524
H	4.615	30.468	21.545
N	3.322	32.855	20.097
H	2.909	30.843	20.235
C	2.155	33.106	19.249
H	1.642	32.170	19.047
H	2.488	33.556	18.314
H	1.486	33.794	19.764
C	4.139	34.018	20.444
H	4.959	33.721	21.088
H	3.508	34.745	20.954
H	4.526	34.459	19.525
O	1.570	31.272	22.739
H	2.029	31.770	23.427
H	1.968	30.386	22.778

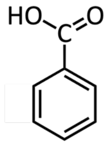

C	2.597	27.470	22.624
C	1.472	26.642	22.586
C	1.550	25.343	23.065
C	2.747	24.865	23.589
C	3.870	25.686	23.630
C	3.798	26.982	23.145
C	2.572	28.864	22.144
H	0.532	26.978	22.168
H	0.679	24.704	23.025
H	2.804	23.851	23.964
H	4.800	25.315	24.040
H	4.661	27.631	23.178
O	3.590	29.536	22.025
O	1.409	29.441	21.848
H	1.099	24.378	18.460
H	3.632	24.838	18.899
H	0.727	24.641	20.175
C	0.883	25.137	19.215
H	3.202	24.974	20.615
C	3.287	25.523	19.677
N	1.991	26.072	19.315
C	1.826	27.372	19.072
O	2.716	28.230	19.135
H	0.794	27.639	18.809
H	-0.024	25.670	18.937
H	4.000	26.333	19.792
H	0.573	30.731	24.587
H	3.129	27.847	26.160
H	0.001	29.932	26.067
C	0.554	29.791	25.134
H	1.636	28.125	27.087
C	2.072	28.080	26.085
N	1.912	29.356	25.409
C	2.964	30.153	25.157
O	4.137	29.902	25.427
H	2.674	31.087	24.654
H	0.035	29.039	24.536
H	1.565	27.299	25.517

O	3.546	31.969	21.276
C	2.369	32.399	20.980
H	3.525	30.989	21.573
N	2.169	33.606	20.557
H	1.515	31.747	21.105
C	0.820	34.062	20.227
H	0.105	33.269	20.424
H	0.786	34.336	19.172
H	0.581	34.935	20.833
C	3.254	34.562	20.340
H	4.203	34.117	20.618
H	3.066	35.448	20.946
H	3.268	34.844	19.287
H	0.645	28.873	22.026
O	2.026	30.844	18.626
H	2.243	29.902	18.809
H	1.076	30.858	18.461

Structures in 3 DMSO and 1 H_2O

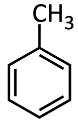

C	2.420	29.478	24.246
C	1.331	28.605	24.300
C	1.340	27.405	23.598
C	2.440	27.058	22.819
C	3.526	27.923	22.747
C	3.513	29.122	23.455
C	2.421	30.746	25.051
H	0.469	28.870	24.900
H	0.486	26.742	23.657
H	2.446	26.125	22.269
H	4.385	27.667	22.138
H	4.364	29.791	23.393
C	-1.155	28.740	20.929
S	-0.629	30.447	20.670
H	-0.845	28.145	20.069
H	-2.241	28.750	21.004
H	-0.705	28.366	21.847
C	1.151	30.150	20.689
H	1.405	29.522	19.835
H	1.424	29.668	21.626
H	1.637	31.120	20.598
O	-0.959	31.193	21.943
H	4.822	26.299	26.548
H	2.454	25.301	27.212
S	3.308	27.370	28.049
C	2.034	26.297	27.352
C	4.444	27.317	26.646
H	1.219	26.261	28.073
O	2.725	28.761	28.094
H	5.263	27.997	26.876
H	3.923	27.631	25.743
H	1.696	26.716	26.405
O	0.068	34.525	25.336
S	-0.210	33.484	24.262
C	-1.591	34.122	23.301
H	-1.749	33.441	22.467
H	-1.353	35.131	22.963
H	-2.459	34.129	23.959
C	1.078	33.691	23.021
H	2.018	33.408	23.491
H	1.103	34.732	22.700
H	0.853	33.021	22.193
H	1.432	31.205	25.050
H	3.141	31.466	24.661
H	2.681	30.533	26.091
O	2.043	33.717	27.114
H	2.752	33.372	26.560
H	1.345	33.998	26.482

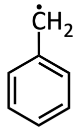

C	3.437	29.117	23.523
C	2.034	28.890	23.519
C	1.517	27.610	23.490
C	2.369	26.502	23.471
C	3.754	26.697	23.478

C	4.283	27.973	23.502
C	3.961	30.415	23.550
H	1.370	29.744	23.545
H	0.444	27.469	23.486
H	1.960	25.500	23.453
H	4.418	25.842	23.465
H	5.356	28.118	23.507
C	-0.536	29.752	20.742
S	0.797	30.631	19.899
H	-0.898	28.959	20.088
H	-1.328	30.476	20.927
H	-0.164	29.346	21.680
C	1.958	29.252	19.791
H	1.516	28.481	19.158
H	2.159	28.870	20.791
H	2.868	29.634	19.332
O	1.373	31.610	20.894
H	2.390	27.398	27.712
H	-0.147	28.012	27.331
S	1.574	29.641	27.650
C	0.096	28.965	26.861
C	2.709	28.308	27.203
H	-0.710	29.678	27.025
O	1.981	30.850	26.844
H	3.697	28.605	27.550
H	2.702	28.177	26.122
H	0.288	28.841	25.797
O	-1.929	31.082	24.192
S	-0.606	31.829	24.228
C	-0.758	33.165	23.031
H	0.099	33.828	23.150
H	-1.693	33.694	23.211
H	-0.734	32.715	22.042
C	-0.652	32.837	25.720
H	-0.599	32.158	26.567
H	-1.577	33.412	25.728
H	0.220	33.491	25.719
H	3.305	31.275	23.565
H	5.029	30.583	23.555
O	-2.146	28.369	23.789
H	-1.995	29.327	23.952
H	-1.733	27.918	24.535

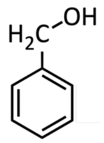

C	0.826	29.247	23.251
C	1.304	27.959	23.505
C	2.666	27.689	23.481
C	3.575	28.708	23.201
C	3.105	29.987	22.929
C	1.738	30.252	22.948
C	-0.662	29.510	23.347
H	0.602	27.165	23.734
H	3.020	26.686	23.684
H	4.637	28.501	23.185
H	3.801	30.784	22.699
H	1.383	31.247	22.721
O	-1.058	30.819	22.979
H	-0.749	31.463	23.664
H	-0.988	29.292	24.371
H	-1.197	28.819	22.691
C	-0.127	28.509	19.511
S	1.359	29.114	18.689
H	-0.238	27.451	19.275
H	-0.969	29.073	19.115
H	-0.026	28.662	20.584
C	2.552	28.146	19.632
H	2.392	27.092	19.405
H	2.417	28.344	20.693
H	3.543	28.453	19.303
O	1.541	30.556	19.121
H	2.813	28.561	27.315
H	0.826	26.858	27.565
S	0.594	29.218	27.864
C	0.087	27.596	27.255
C	2.142	29.337	26.945
H	-0.878	27.373	27.707
O	-0.367	30.216	27.263
H	2.563	30.320	27.147
H	1.949	29.209	25.882
H	0.005	27.637	26.169

O	-0.269	32.672	24.759
S	1.082	32.553	25.448
C	2.107	33.845	24.723
H	3.051	33.882	25.267
H	1.581	34.798	24.782
H	2.285	33.564	23.686
C	0.861	33.285	27.076
H	0.202	32.614	27.621
H	0.427	34.278	26.967
H	1.836	33.333	27.562
O	-0.535	31.919	20.438
H	-0.680	31.489	21.303
H	0.201	31.429	20.024

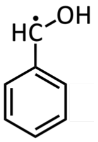

C	1.650	28.954	23.217
C	0.520	28.105	23.073
C	0.669	26.738	22.977
C	1.941	26.153	23.022
C	3.063	26.972	23.172
C	2.933	28.345	23.269
C	1.472	30.340	23.314
H	-0.468	28.549	23.041
H	-0.208	26.111	22.867
H	2.053	25.080	22.946
H	4.050	26.527	23.213
H	3.810	28.967	23.386
O	2.526	31.166	23.463
H	2.195	32.099	23.635
H	0.487	30.790	23.309
C	-0.034	29.285	19.559
S	1.538	29.472	18.699
H	-0.436	28.297	19.332
H	-0.699	30.057	19.176
H	0.129	29.405	20.628
C	2.440	28.159	19.543
H	1.980	27.207	19.279
H	2.408	28.325	20.618
H	3.465	28.192	19.177
O	2.126	30.786	19.178
H	3.317	30.062	27.508
H	2.531	27.533	27.357
S	1.032	29.386	27.458
C	1.643	27.823	26.793
C	2.434	30.416	26.978
H	0.853	27.088	26.938
O	-0.134	29.800	26.592
H	2.200	31.435	27.283
H	2.573	30.360	25.899
H	1.872	27.944	25.736
O	1.602	33.536	23.970
S	1.233	33.853	25.417
C	0.680	35.563	25.376
H	0.304	35.825	26.365
H	-0.097	35.663	24.619
H	1.547	36.173	25.129
C	-0.357	33.066	25.712
H	-0.190	31.990	25.745
H	-1.039	33.342	24.909
H	-0.727	33.411	26.678
O	4.157	31.056	21.060
H	3.696	31.092	21.914
H	3.441	30.930	20.405

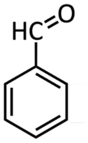

C	2.212	28.680	23.550
C	1.118	27.932	23.115
C	1.227	26.558	22.959
C	2.436	25.927	23.237
C	3.535	26.669	23.670
C	3.423	28.042	23.828
C	2.056	30.134	23.729
H	0.179	28.433	22.916
H	0.375	25.979	22.627
H	2.525	24.854	23.116
H	4.472	26.173	23.884

H	4.265	28.632	24.168
O	2.895	30.858	24.233
H	1.097	30.544	23.381
C	0.359	30.333	19.995
S	2.128	30.368	19.631
H	-0.160	29.883	19.149
H	0.043	31.367	20.122
H	0.189	29.767	20.909
C	2.405	28.584	19.583
H	1.824	28.162	18.763
H	2.114	28.148	20.537
H	3.468	28.436	19.402
O	2.817	30.877	20.872
H	2.303	29.188	28.363
H	1.111	26.886	27.692
S	0.305	29.038	27.084
C	0.765	27.326	26.756
C	1.953	29.669	27.450
H	-0.134	26.816	26.413
O	-0.099	29.639	25.750
H	1.856	30.742	27.605
H	2.617	29.459	26.614
H	1.543	27.294	25.996
O	-0.457	32.648	23.094
S	0.564	33.537	23.765
C	-0.188	35.175	23.878
H	0.469	35.822	24.460
H	-1.167	35.084	24.349
H	-0.283	35.554	22.862
C	0.517	33.136	25.523
H	0.866	32.111	25.620
H	-0.507	33.228	25.883
H	1.186	33.817	26.050
O	-2.440	31.101	25.838
H	-1.608	30.581	25.799
H	-3.130	30.450	26.013

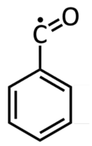

C	2.176	28.897	23.757
C	0.851	28.695	23.369
C	0.461	27.458	22.876
C	1.393	26.429	22.766
C	2.719	26.632	23.148
C	3.113	27.864	23.645
C	2.573	30.205	24.292
H	0.142	29.508	23.460
H	-0.565	27.293	22.576
H	1.087	25.464	22.380
H	3.438	25.828	23.059
H	4.137	28.037	23.950
O	3.604	30.545	24.778
C	0.910	31.027	20.174
S	2.687	30.739	20.034
H	0.434	30.686	19.254
H	0.770	32.100	20.291
H	0.522	30.493	21.039
C	2.623	28.936	19.929
H	2.128	28.664	18.997
H	2.085	28.539	20.787
H	3.653	28.584	19.920
O	3.296	31.079	21.371
H	3.148	27.735	28.397
H	1.190	26.215	27.382
S	1.042	28.577	27.661
C	0.872	27.035	26.737
C	2.844	28.582	27.782
H	-0.183	26.929	26.492
O	0.662	29.698	26.726
H	3.127	29.515	28.265
H	3.277	28.518	26.785
H	1.476	27.079	25.834
O	-0.987	31.620	23.366
S	0.211	32.533	23.495
C	-0.354	34.174	22.996
H	0.445	34.890	23.188
H	-1.254	34.431	23.556

H	-0.564	34.126	21.929
C	0.406	32.865	25.256
H	0.654	31.916	25.727
H	-0.528	33.266	25.650
H	1.223	33.576	25.385
O	5.764	33.189	26.250
H	6.711	33.343	26.138
H	5.462	33.932	26.788

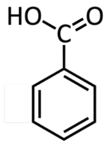

C	1.819	28.783	23.155
C	0.830	27.827	23.391
C	1.173	26.492	23.557
C	2.506	26.102	23.485
C	3.496	27.050	23.244
C	3.154	28.383	23.076
C	1.512	30.221	22.942
H	-0.218	28.098	23.415
H	0.398	25.757	23.731
H	2.772	25.060	23.613
H	4.534	26.749	23.187
H	3.915	29.127	22.888
O	2.303	30.993	22.437
C	1.474	27.110	19.780
S	0.874	28.728	19.245
H	2.089	26.695	18.981
H	0.597	26.484	19.938
H	2.045	27.213	20.700
C	2.470	29.573	19.226
H	3.100	29.099	18.474
H	2.920	29.515	20.215
H	2.276	30.610	18.954
O	0.083	29.312	20.388
H	2.567	27.614	27.669
H	0.194	28.785	27.914
S	2.145	29.892	27.104
C	0.392	29.455	27.077
C	2.795	28.234	26.802
H	-0.172	30.377	27.195
O	2.418	30.706	25.864
H	3.873	28.332	26.685
H	2.346	27.824	25.900
H	0.154	28.974	26.130
O	-0.303	34.317	23.397
S	0.310	33.850	24.704
C	0.407	35.312	25.754
H	0.741	35.004	26.745
H	-0.574	35.786	25.799
H	1.139	35.979	25.302
C	-1.007	33.003	25.596
H	-1.229	32.091	25.048
H	-1.880	33.655	25.640
H	-0.646	32.762	26.596
O	0.315	30.686	23.318
H	-0.210	30.017	23.779
O	1.203	33.360	21.246
H	1.603	32.564	21.639
H	0.643	33.719	21.964

Structures in 2 DMSO, 1 MSA, and 1 H_2O

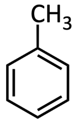

C	0.576	28.643	23.705
C	0.362	27.311	23.348
C	1.422	26.490	22.973
C	2.720	26.990	22.950
C	2.947	28.314	23.313
C	1.885	29.127	23.688
C	-0.570	29.544	24.072
H	-0.646	26.912	23.364
H	1.233	25.458	22.701
H	3.546	26.352	22.661
H	3.953	28.715	23.307
H	2.079	30.152	23.974
C	1.314	30.000	18.577
S	1.259	29.970	20.383

C	2.728	30.977	20.675
H	3.601	30.435	20.310
H	2.801	31.125	21.749
H	2.612	31.931	20.159
O	0.078	30.823	20.781
H	1.776	26.088	26.897
S	0.613	28.137	27.216
C	0.425	27.628	28.936
C	2.059	27.137	26.822
O	1.080	29.578	27.265
H	2.860	27.379	27.520
H	2.345	27.377	25.801
O	2.733	32.471	24.496
S	1.338	32.939	24.728
C	0.682	33.265	23.089
H	1.395	33.898	22.557
H	-0.283	33.757	23.200
H	0.565	32.308	22.581
O	1.396	34.471	25.292
H	-1.471	28.970	24.286
H	-0.789	30.227	23.247
H	-0.328	30.148	24.948
H	1.841	35.066	24.660
H	2.219	29.492	18.246
H	1.297	31.035	18.236
H	0.434	29.463	18.225
H	0.180	26.566	28.960
H	-0.396	28.211	29.351
H	1.354	27.830	29.469
O	3.562	30.677	26.603
H	3.328	31.282	25.878
H	2.719	30.245	26.846

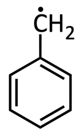

C	0.873	29.376	24.708
C	0.691	28.198	23.934
C	1.755	27.602	23.287
C	3.037	28.150	23.381
C	3.244	29.304	24.143
C	2.189	29.907	24.797
C	-0.207	29.992	25.358
H	-0.303	27.776	23.855
H	1.595	26.705	22.703
H	3.867	27.681	22.870
H	4.238	29.727	24.221
H	2.353	30.798	25.392
C	0.840	28.612	19.575
S	-0.143	30.035	20.091
C	1.129	30.839	21.090
H	1.947	31.129	20.430
H	1.472	30.149	21.859
H	0.673	31.720	21.535
O	-1.191	29.511	21.043
H	3.539	25.980	26.798
S	1.285	25.751	27.531
C	0.997	24.837	26.006
C	2.732	26.684	26.998
O	0.146	26.750	27.634
H	3.005	27.344	27.820
H	2.482	27.258	26.108
O	-1.187	33.764	25.512
S	-0.836	33.791	24.071
C	0.964	33.729	24.026
H	1.297	32.881	24.624
H	1.321	34.665	24.453
H	1.286	33.635	22.990
O	-1.159	32.325	23.421
H	-1.199	29.563	25.315
H	-0.058	30.876	25.966
H	-0.734	31.600	23.930
H	1.670	28.965	18.962
H	0.188	27.968	18.988
H	1.201	28.094	20.463
H	1.846	24.174	25.836
H	0.878	25.542	25.186
H	0.090	24.253	26.152
O	0.647	29.278	28.628
H	0.475	28.383	28.265
H	0.810	29.827	27.852

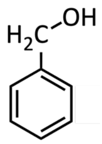

C	0.988	29.102	23.189
C	0.696	27.757	23.422
C	1.703	26.799	23.433
C	3.025	27.174	23.206
C	3.325	28.512	22.973
C	2.314	29.468	22.967
C	-0.127	30.122	23.213
H	-0.333	27.457	23.587
H	1.457	25.760	23.611
H	3.811	26.430	23.209
H	4.349	28.816	22.795
H	2.561	30.503	22.780
O	0.126	31.287	22.444
H	0.732	31.880	22.930
H	-0.328	30.391	24.255
C	1.513	27.769	19.654
S	0.550	28.997	18.746
H	2.536	27.779	19.277
H	1.053	26.801	19.461
H	1.485	28.007	20.715
C	1.439	30.463	19.314
O	-0.814	29.043	19.393
H	0.117	28.034	27.954
S	1.454	29.883	27.300
C	-0.013	28.847	27.240
C	2.647	28.692	26.676
H	-0.856	29.472	27.528
O	1.250	30.900	26.180
H	-0.135	28.466	26.227
O	1.920	33.113	23.484
S	1.899	33.938	24.741
O	2.538	33.083	25.930
C	0.165	33.917	25.225
H	-0.385	34.461	24.458
H	0.073	34.417	26.188
H	-0.166	32.881	25.282
H	2.044	32.195	26.086
H	-1.033	29.666	22.808
H	0.973	31.323	18.836
H	2.481	30.381	19.002
H	1.348	30.530	20.397
H	2.748	27.897	27.416
H	3.592	29.220	26.564
H	2.303	28.302	25.721
O	4.028	32.635	21.691
H	3.310	32.843	22.320
H	3.623	32.052	21.039

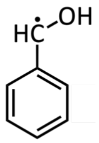

C	1.655	28.469	23.055
C	0.456	27.707	23.051
C	0.483	26.343	22.845
C	1.698	25.677	22.636
C	2.888	26.410	22.640
C	2.879	27.778	22.846
C	1.599	29.855	23.248
H	-0.488	28.216	23.207
H	-0.444	25.784	22.844
H	1.714	24.607	22.474
H	3.831	25.903	22.478
H	3.806	28.335	22.841
O	2.726	30.591	23.297
H	2.494	31.552	23.382
H	0.657	30.375	23.366
C	0.619	29.066	19.621
S	1.717	30.172	18.715
H	-0.323	29.037	19.076
H	0.477	29.453	20.628
H	1.070	28.075	19.647
C	3.136	30.085	19.821
O	1.141	31.568	18.867
H	1.555	29.662	28.946
S	1.544	30.243	26.631
C	0.779	29.797	28.194
C	2.644	28.837	26.448

H	0.110	30.613	28.463
O	2.455	31.426	26.960
H	0.210	28.880	28.044
O	2.148	33.178	23.340
S	1.679	34.027	24.494
O	2.544	33.668	25.780
C	0.086	33.317	24.939
H	-0.597	33.548	24.123
H	-0.253	33.780	25.864
H	0.195	32.238	25.047
H	2.433	32.715	26.157
H	3.898	30.744	19.408
H	3.498	29.057	19.837
H	2.840	30.408	20.817
H	3.255	29.028	25.570
H	2.035	27.947	26.292
H	3.258	28.748	27.344
O	0.151	32.643	21.233
H	0.559	32.231	20.442
H	0.877	32.810	21.853

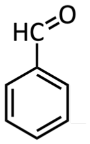

C	1.888	29.978	23.815
C	0.504	29.986	23.642
C	-0.154	28.827	23.255
C	0.570	27.656	23.047
C	1.954	27.644	23.220
C	2.613	28.804	23.600
C	2.559	31.226	24.215
H	-0.047	30.907	23.789
H	-1.227	28.833	23.113
H	0.058	26.750	22.748
H	2.513	26.731	23.058
H	3.687	28.812	23.736
O	3.766	31.370	24.291
H	1.554	30.250	20.128
C	2.156	31.938	20.294
S	1.990	29.831	19.221
H	0.472	30.310	20.030
H	1.834	29.670	21.005
H	3.884	31.575	20.629
C	1.603	32.468	21.616
O	0.565	27.683	27.642
H	1.714	29.755	27.856
S	0.544	28.619	27.085
C	3.230	28.876	27.436
C	-0.440	29.079	27.165
H	1.716	31.019	27.018
O	0.813	28.464	26.043
H	-1.488	32.772	23.803
O	-1.273	34.022	23.012
S	-0.688	33.636	21.568
O	0.194	34.774	23.744
C	0.219	33.166	21.598
H	4.304	31.099	19.743
H	3.953	30.923	21.497
H	4.379	32.526	20.815
H	4.059	29.483	27.794
H	3.282	28.743	26.357
H	3.219	27.915	27.951
H	0.455	35.659	23.166
H	1.001	34.043	23.733
H	-0.065	35.046	24.766
H	-0.707	32.339	26.506
O	0.141	31.865	26.604
H	-0.857	32.450	25.551
H	1.878	32.058	24.444

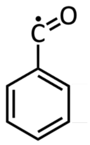

C	1.866	30.066	23.790
C	0.488	30.059	23.583
C	-0.148	28.877	23.228
C	0.592	27.707	23.081
C	1.972	27.714	23.285
C	2.612	28.891	23.638
C	2.528	31.322	24.171

H	-0.073	30.978	23.684
H	-1.217	28.867	23.063
H	0.095	26.786	22.805
H	2.541	26.801	23.169
H	3.682	28.913	23.800
O	3.685	31.548	24.332
C	1.425	30.278	20.045
S	2.120	31.931	20.200
H	1.811	29.842	19.123
H	0.345	30.394	19.982
H	1.702	29.679	20.910
C	3.837	31.471	20.473
O	1.643	32.477	21.543
H	0.666	27.699	27.610
S	1.575	29.882	27.872
C	0.565	28.631	27.055
C	3.190	29.161	27.524
H	-0.465	28.981	27.094
O	1.486	31.140	27.030
H	0.892	28.512	26.024
O	-1.586	32.714	23.690
S	-1.120	34.034	23.161
O	-0.538	33.832	21.678
C	0.413	34.357	24.053
H	0.315	33.273	21.629
H	4.203	30.986	19.568
H	3.896	30.802	21.330
H	4.389	32.391	20.656
H	3.937	29.853	27.910
H	3.301	29.028	26.450
H	3.257	28.207	28.047
H	0.883	35.244	23.629
H	1.057	33.481	23.965
H	0.142	34.529	25.094
O	-1.135	31.986	26.427
H	-0.208	31.711	26.566
H	-1.216	32.202	25.482

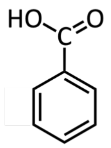

C	1.703	28.919	23.605
C	0.839	27.875	23.273
C	1.348	26.624	22.956
C	2.723	26.407	22.970
C	3.589	27.447	23.296
C	3.082	28.698	23.609
C	1.221	30.279	23.939
H	-0.232	28.025	23.226
H	0.673	25.821	22.693
H	3.118	25.429	22.725
H	4.658	27.280	23.309
H	3.744	29.513	23.864
O	1.967	31.246	23.947
C	2.083	29.010	19.910
S	1.086	30.517	19.930
C	2.377	31.644	20.498
H	3.166	31.673	19.747
H	2.756	31.301	21.459
H	1.920	32.628	20.596
O	0.098	30.382	21.062
H	1.965	26.150	27.412
S	1.307	28.425	27.606
C	-0.339	27.894	27.093
C	2.238	27.062	26.882
O	1.641	29.656	26.787
H	3.293	27.282	27.032
H	2.005	26.984	25.822
O	-0.818	33.763	25.330
S	0.042	34.399	24.290
C	-0.474	33.645	22.735
H	0.198	33.985	21.949
H	-1.489	33.991	22.542
H	-0.450	32.561	22.835
O	1.534	33.798	24.404
O	-0.062	30.465	24.233
H	-0.564	29.638	24.238
H	1.580	32.805	24.254
H	2.839	29.113	19.132

H	1.409	28.189	19.670
H	2.542	28.860	20.885
H	-0.586	26.978	27.630
H	-0.350	27.729	26.016
H	-1.029	28.688	27.371
O	-0.173	31.783	27.289
H	0.455	31.091	27.011
H	-0.326	32.348	26.512
